# Human Mesenchymal Stem Cell‐Derived Miniature Joint System for Disease Modeling and Drug Testing

**DOI:** 10.1002/advs.202105909

**Published:** 2022-04-18

**Authors:** Zhong Li, Zixuan Lin, Silvia Liu, Haruyo Yagi, Xiurui Zhang, Lauren Yocum, Monica Romero‐Lopez, Claire Rhee, Meagan J. Makarcyzk, Ilhan Yu, Eileen N. Li, Madalyn R. Fritch, Qi Gao, Kek Boon Goh, Benjamen O'Donnell, Tingjun Hao, Peter G. Alexander, Bhushan Mahadik, John P. Fisher, Stuart B. Goodman, Bruce A. Bunnell, Rocky S. Tuan, Hang Lin

**Affiliations:** ^1^ Center for Cellular and Molecular Engineering Department of Orthopaedic Surgery University of Pittsburgh School of Medicine Pittsburgh PA 15219 USA; ^2^ Department of Pathology University of Pittsburgh School of Medicine Pittsburgh PA 15261 USA; ^3^ Department of Orthopaedic Surgery Stanford University Stanford CA 94305 USA; ^4^ Department of Bioengineering University of Pittsburgh Swanson School of Engineering Pittsburgh PA 15260 USA; ^5^ Institute of Physics University of Freiburg Freiburg 79104 Germany; ^6^ School of Engineering Monash University Malaysia Selangor 47500 Malaysia; ^7^ Center for Stem Cell Research and Regenerative Medicine Tulane University School of Medicine Orleans LA 70112 USA; ^8^ Fischell Department of Bioengineering University of Maryland College Park MD 20742 USA; ^9^ McGowan Institute for Regenerative Medicine University of Pittsburgh School of Medicine Pittsburgh PA 15219 USA; ^10^ Present address: Department of Microbiology, Immunology, and Genetics University of North Texas Health Science Center Fort Worth TX 76107 USA; ^11^ Present address: The Chinese University of Hong Kong Shatin Hong Kong SAR 999077 China

**Keywords:** arthritis, human mesenchymal stem cells, inflammation in joint, joint diseases, microphysiological system

## Abstract

Diseases of the knee joint such as osteoarthritis (OA) affect all joint elements. An in vitro human cell‐derived microphysiological system capable of simulating intraarticular tissue crosstalk is desirable for studying etiologies/pathogenesis of joint diseases and testing potential therapeutics. Herein, a human mesenchymal stem cell‐derived miniature joint system (miniJoint) is generated, in which engineered osteochondral complex, synovial‐like fibrous tissue, and adipose tissue are integrated into a microfluidics‐enabled bioreactor. This novel design facilitates different tissues communicating while still maintaining their respective phenotypes. The miniJoint exhibits physiologically relevant changes when exposed to interleukin‐1*β* mediated inflammation, which are similar to observations in joint diseases in humans. The potential of the miniJoint in predicting in vivo efficacy of drug treatment is confirmed by testing the “therapeutic effect” of the nonsteroidal anti‐inflammatory drug, naproxen, as well as four other potential disease‐modifying OA drugs. The data demonstrate that the miniJoint recapitulates complex tissue interactions, thus providing a robust organ chip model for the study of joint pathology and the development of novel therapeutic interventions.

## Graphical abstract

A miniature synovial joint‐mimicking system (miniJoint) is established in a microfluidics‐enabled bioreactor, in which human mesenchymal stem cell‐derived bone‐cartilage complex, synovial‐like fibrous tissue, and adipose tissue are integrated and show active crosstalk. The data demonstrate the potential of miniJoint as a new model for the study of joint pathology and the development of novel therapeutic interventions.

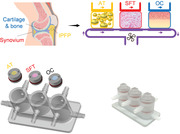

## 1 Introduction

Diseases of the knee joint such as osteoarthritis (OA) are highly prevalent, debilitating disorders that severely compromise the quality of life, creating a significant socioeconomic burden for affected individuals.^[^ ^1^ ^]^ Although cartilage degeneration is a common feature in many joint diseases, the inseparable tissue interconnections inevitably result in pathogenic changes in other joint elements, such as synovitis, remodeling of the subchondral bone and meniscal degeneration.^[^ ^2^ ^]^ The articular cartilage is primarily anatomically bound to the subchondral bone, forming an osteochondral unit. The biophysical and biochemical communication between cartilage and bone plays a crucial role in maintaining joint homeostasis and mediating pathological changes.^[^ ^3^ ^]^ Cartilage, synovium, and other intraarticular tissues are bathed in synovial fluid, the lubricant found in the joint cavity, which serves as the vehicle for mediating the molecular and cellular crosstalk among these tissues.^[^ ^4^ ^]^ While conventional in vitro tissue culture platforms and different animal models have significantly increased our understanding of disease etiologies and pathogenesis, some critical challenges remain. For example, multicomponent, real‐time, and bi‐directional crosstalk information has rarely been observed in current in vitro models of the joint diseases. Moreover, there are significant differences in pathology and drug responses between animal models and humans.^[^ ^5^ ^]^ Therefore, a human cell‐derived model of the joint is urgently needed to enhance our understanding of the pathogenesis of joint diseases in humans and facilitate the discovery of novel drug treatments.^[^ ^5^ ^]^

Tissue‐on‐a‐chip technology offers a 3D engineered system that mimics the physiological microenvironment.^[^ ^6^ ^]^ Models generated using this technology aim to emulate the in vivo microenvironment characterized by diverse cellular composition, tissue‐specific extracellular matrix, and interactive biochemical and physical signals. Using human cells and integrating multiple tissues, the limitations of current animal models and in vitro culture assays can be partially overcome.^[^ ^7^ ^]^ As the first step toward whole joint construction in vitro, we have previously created a biphasic osteochondral model, which demonstrates active crosstalk between bone and cartilage throughout tissue maturation and disease progression.^[^ ^8^ ^]^ Recently, a chondrocyte‐derived cartilage chip was developed by Occhetta et al. to simulate the influence of mechanical load on cartilage.^[^ ^9^ ^]^

**Figure 1:**
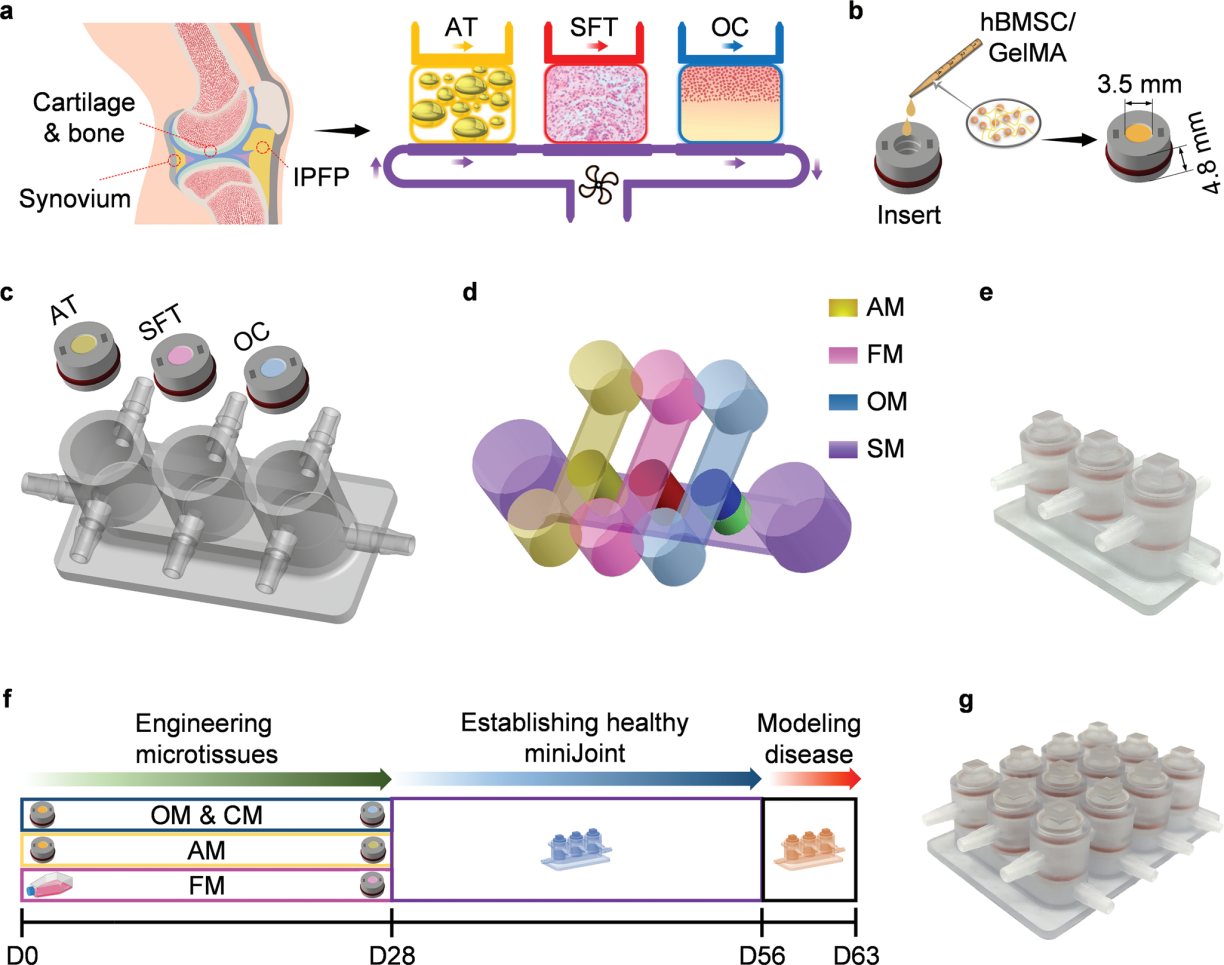
The miniJoint—a miniature system mimicking the knee joint in the human body. a) The miniJoint chip comprises engineered adipose tissues (AT), synovial‐like fibrous tissues (SFT), osteochondral tissues (OC) to simulate fat tissue, synovium, and cartilage‐bone complex in the native knee joint. Crosstalk between tissues was realized via either diffusion (within OC) or fluidic flow. b) The microtissue modules were generated by photo‐crosslinking hBMSC‐laden GelMA placed in 3D printed inserts. c,d) The miniJoint chip was established by integrating differentiated microtissues in a 3D printed chamber c), and then perfusing tissue‐specific medium (AM, FM, and OM; yellow, pink, and blue) streams on the top and a commonly shared medium (SM; purple) stream simulating the function of synovial fluid at the bottom d). A photograph of an assembled miniJoint chip is shown in e). f) Timeline (up to day 63) of generating miniJoint culture and modeling joint disease. g) A high‐yield miniJoint chip capable of producing four replicates of each microtissue. hBMSC, human bone marrow‐derived mesenchymal stem cell; GelMA, methacrylated gelatin; AM, adipogenic medium; FM, fibrogenic medium; OM, osteogenic medium.

In general, the utility of current articular organ chip models has been limited by the lack of inclusion of the primary joint tissues or elements, and thus have limited relevance in the context of clinical settings.^[^ ^5^ ^]^ Here, we report the development and characterization of a 3D, multicomponent, and human cell‐based miniature synovial joint system (miniJoint) (**Figure** **1**a), which contains bone (osteoblasts), cartilage (chondrocytes), synovial‐like fibrous tissue (fibroblasts), and adipose tissues (adipocytes). This versatile miniJoint chip employs a modular design concept, which allows convenient integration and interconnection of multiple tissue modules, enabling their crosstalk and simulating in vivo physiology. In this manner, the effects of secreted products from one tissue to another can be examined in a physiologically relevant setting to more precisely predict the efficacy and toxicity of experimental treatments.^[^ ^10^ ^]^

Because mesenchymal stem cells (hMSCs) can be isolated from different human tissue sources and expanded to a relatively high number without significantly losing their original stem cell capabilities, they are an ideal cell source for creating the miniJoint. Additionally, hMSCs have demonstrated robust osteogenic, chondrogenic, and adipogenic differentiation capacity.^[^ ^11^ ^]^ In this study, the individual joint elements were initially derived from human bone marrow‐derived MSCs (hBMSCs) separately and then integrated into a custom‐designed bioreactor to generate the complete miniJoint (Figure 1b–e,g). The ability of the engineered tissues to maintain their respective phenotypes was examined through molecular and biochemical analyses. To assess the ability of the miniJoint to respond to external signals, interleukin‐1*β* (IL‐1*β*), a classic cytokine widely used to induce OA‐like features in vitro, was introduced to simulate the inflammatory features observed in most joint diseases (Figure 1f). Transcriptome analysis of inflamed and control miniJoint tissues was conducted using RNA sequencing (RNA‐Seq). To test the utility of the miniJoint in drug screening, a representative anti‐inflammatory agent and several potential disease‐modifying OA drugs (DMOADs) were introduced via “systemic administration” or “intraarticular injection” in the disease‐modeling miniJoint.

## 2 Results

### 2.1 Engineering the Individual miniJoint Components

**Figure 2:**
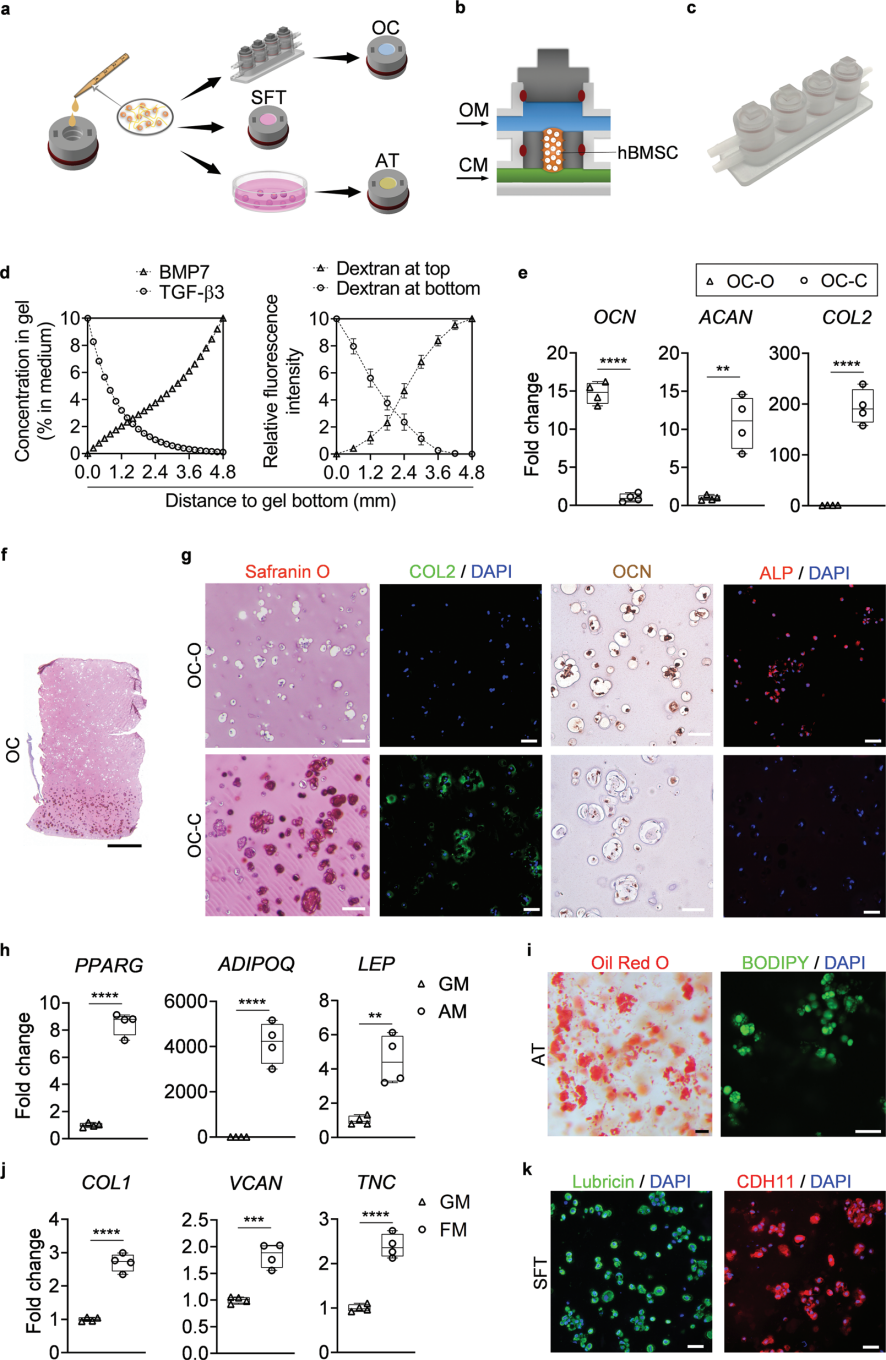
Engineering and characterization of individual miniJoint tissue components. a) Experimental schematic showing the fabrication of different miniJoint tissue components. OC, osteochondral tissue; SFT, synovial‐like fibrous tissue; AT, adipose tissue. b,c) A dual‐flow chip, allowing perfusion of osteogenic medium (OM) above the MSC‐laden gel insert and chondrogenic medium (CM) below the gel insert b), was 3D printed c) and used to generate the biphasic OC microtissues. d) Simulated and measured biomolecule diffusion within the osteochondral tissue. Assuming a partitioning coefficient of 0.1, equilibrated distribution profile of BMP7, an osteogenic growth factor in OM, and TGF*β*3, a chondrogenic growth factor in CM, in the OC microtissue was calculated (left). For validation purposes, dextran (molecular weight, 20 kDa) labeled with fluorescein isothiocyanate was added to either the top or bottom stream of a dual‐flow bioreactor to simulate the diffusion of BMP7 and TGF*β*3, respectively (right). The relative fluorescence intensity was normalized and set in the range of 0–10. *N* = 4 for the dextran diffusion test. e) Box plots showing the expression of *OCN*, *COL2*, and *ACAN* in the bone (OC‐O) and cartilage (OC‐C) components of the OC microtissues. OCN data were normalized to values in OC‐C, and COL2 and ACAN data to values in OC‐O. Data were analyzed by the Student's *t*‐test (*N* = 4 biological replicates). **, *p* < 0.01; ****, *p* < 0.0001. f) Safranin O staining of the entire OC unit. Scale bar = 1 mm. g) Safranin O staining and immunostaining images showing prominent GAGs (Safranin O) and COL2 presence (immunostaining) in OC‐C, and OCN and ALP (immunostaining) in OC‐O. Scale bar = 50 µm. h) Box plots showing the expression of peroxisome proliferator‐activated receptor gamma (*PPARG*), adiponectin (*ADPOQ*), and leptin (*LEP*) in AT microtissues. Data were analyzed by the Student's *t*‐test (*N* = 4 biological replicates). ***, *p* < 0.001; ****, *p* < 0.0001. GM, growth medium; AM, adipogenic medium. i) Oil Red O and BODIPY staining images showing deposition of lipid droplets in the AT microtissue. Scale bar = 50 µm. j) Box plots showing the expression of *COL1*, *VCAN*, and *TNC* in SFT microtissues. Data were analyzed by the Student's *t*‐test (*N* = 4 biological replicates). ***, *p* < 0.001; ****, *p* < 0.0001. FM, fibrogenic medium. k) Immunostaining of lubricin and CDH11 for the FT. Scale bar = 50 µm.

In the miniJoint (Figure 1a), engineered tissues that partially recapitulate the phenotypes and functions of native bone, cartilage, synovium, and fat pad were included. These tissues were created using the same pooled population of hBMSCs. The biologic properties of hBMSCs were first verified using conventional MSC characterization protocols, including colony formation‐unit (CFU) assay, cell surface marker profiling, and tri‐lineage differentiation (Figure S1, Supporting Information). A hydrogel scaffold prepared with methacrylated gelatin (GelMA) was used to create 3D constructs and support hBMSC differentiation, as described in our previous studies.^[^ ^8^ ^]^ The scaffold also acted as a barrier to minimize free medium exchange between the top and bottom flows (Figure 1d). Our goal was to fabricate osteochondral (OC), adipose (AT), and synovial‐like fibrous (SFT, to mimic synovium) tissues as modules to generate a “plug and play” miniature joint‐mimicking system (**Figure** **2**a).

The biphasic nature of native OC tissue, consisting of osseous and chondral components in direct contact, presented technical challenges, versus a uniform construct incorporating a single cell type. Previously, we had successfully generated this heterogeneous tissue by employing a dual‐flow OC bioreactor, in which osteogenic and chondrogenic media were perfused separately through the top and bottom streams.^[^ ^8a^ ^]^ Our recent study further demonstrated that the presence of osseous tissue promoted cartilage formation in the chondral component, signifying active crosstalk between the bone and cartilage components.^[^ ^8b^ ^]^ A similar OC fabrication strategy was used here (Figure 2b,c). To further promote the quality of the engineered OC tissues, we optimized the protocol of osteogenic induction by testing the effect of 1,25‐dihydroxyvitamin D_3_ (VitD3, 1 × 10^−8^ m) supplementation and varying dexamethasone and bone morphogenetic protein 7 (BMP7) treatment times. Based on the results shown in Figure S2 (Supporting Information), an optimal osteoinduction method was developed (Table S1, Supporting Information). Since VitD3 is a strong osteoinductive agent, a potential concern was whether the diffused VitD3 from osteogenic medium would result in bone formation in the cartilage layer in the dual‐flow bioreactor culture. As demonstrated in Figure S3 (Supporting Information), the addition of VitD3 in the chondrogenic medium did not significantly inhibit the chondrogenesis of hBMSCs.

The key bioactive factors driving MSC differentiation in the osteochondral tissue are transforming growth factor‐beta 3 (TGF*β*3) in the chondrogenic medium (CM) and BMP7 in the osteogenic medium (OM), respectively. Quantitative modeling of growth factor diffusion in the GelMA scaffold showed that the concentrations of TGF*β*3 and BMP7 decreased by 98.95% and 100%, respectively, over a distance of 4.8 mm (Figure 2d). We also used fluorescently labeled dextran to simulate the diffusion of BMP7 (by adding to the top stream) and TGF*β*3 (by introducing to the bottom stream). From one end of the scaffold to the other, the corresponding dye concentration, indicated by fluorescence intensity, was found to decrease by 99.84% and 99.94%, respectively (Figure 2d; and Figure S4, Supporting Information), which generally agreed well with the simulation results. Taken together, simulation and experimental data both indicated minimal diffusion of growth factors from one medium stream into the other.

After a 28‐day differentiation, RT‐qPCR results showed that in the matured OC microtissue, the osseous component (OC‐O) had significantly higher osteocalcin (*OCN*) expression levels, and the chondral phase (OC‐C) showed much higher collagen type II (*COL2*) and aggrecan (*ACAN*) expression (Figure 2e; and Table S2, Supporting Information). Histological and immunostaining results (Figure 2f,g; and Figure S5, Supporting Information) also confirmed the results of the RT‐qPCR assay. For instance, OCN and alkaline phosphatase (ALP) proteins were identified mostly in OC‐O, while widespread glycosaminoglycans (GAG) and COL2 staining were observed only in OC‐C. Lubricin, a glycoprotein secreted by native chondrocytes, was also observed in OC‐C (Figure S6, Supporting Information). These results indicated the successful generation of a biphasic OC microtissue.

The AT microtissues were created by culturing inserts that housed hBMSCs‐encapsulated GelMA in adipogenic medium for 28 days (Figure 2a). RT‐qPCR results indicated that the expression levels of primary adipogenic marker genes increased significantly upon induction (Figure 2h; and Table S2, Supporting Information). Also, intracellular lipid droplets, a key marker of adipocytes, were positively stained by both Oil Red O and BODIPY fluorophore in the engineered AT microtissues (Figure 2i).

As fibrogenic differentiation of hBMSCs has not been widely reported in the literature, we carried out a direct comparison of several protocols using 2D hBMSC cultures and determined an optimal procedure (Figure S7a, Supporting Information). Since direct 3D fibrogenic induction via differentiating hBMSC in GelMA was noticeably less efficient than 2D differentiation (Figure S7b, Supporting Information), a two‐step procedure was developed and adopted in this study. hBMSCs were first induced in 2D culture for 3 weeks in the fibrogenic medium (Table S1, Supporting Information); the predifferentiated cells were then encapsulated in GelMA within the inserts to generate the SFT component. Compared to naïve hBMSCs [i.e., cells expanded in growth medium (GM)], the SFT microtissues containing pre‐differentiated cells showed higher expression levels of fibrogenic markers, including collage type 1 (*COL1)*, tenascin C (*TNC)* and versican (*VCAN)* (Figure 2j; and Table S2, Supporting Information). Immunofluorescence (IF) showed the ubiquitous presence of key characteristic synovium‐associated proteins, including lubricin and cadherin 11 (CDH11) in the engineered SFT microtissue (Figure 2k). In addition, the SFT microtissues were also positive for collagen type 1 (COL1), a fibroblast marker, and CD44 and *β*1 integrins, two adhesion molecules expressed by synovial fibroblasts (Figure S8, Supporting Information).

### 2.2 Generating the Multitissue miniJoint Chip

After confirmation of their respective phenotypes, OC, AT, and SFT tissue modules were integrated into the miniJoint chamber (Figure 1e,g), and their interconnection was established via directional fluidic flow of the culture medium or diffusion (between OC‐O and OC‐C) (Figure 1c,d). Given the current lack of “universal medium” capable of maintaining the phenotypes of all tissues, the top compartment of each tissue chamber was filled with the respective tissue‐specific medium. To enable crosstalk among OC‐C, AT, and SFT, as in a native joint, a commonly shared medium (SM) exposed to these three components of the miniJoint was used in the bottom stream to simulate the “synovial fluid” (Video S1, Supporting Information; SM composition provided in Table S1, Supporting Information). The fluid velocity and shear stress within the miniJoint were simulated by computational fluid dynamics (CFD) analysis using Fluent 2020 R1 software (Fluent‐Ansys Inc, Canonsburg, PA). To validate the CFD data, the fluid velocity in the bottom chamber of the miniJoint chip was also measured by tracking flowing fluorescent particles and compared with the simulation data (Figure S9, Supporting Information). The simulated shear stress on the microtissue surfaces varied between 16.5 and 4959 μPa (Figure S9g, Supporting Information). Since the presence of TGF*β*3 was required to maintain the cartilaginous phenotype of the engineered cartilage tissue component (Figure S10, Supporting Information), 0.5 ng mL^−1^ TGF*β*3 was added to the SM to emulate the natural chondrosupportive capacity of the synovial fluid.

**Figure 3:**
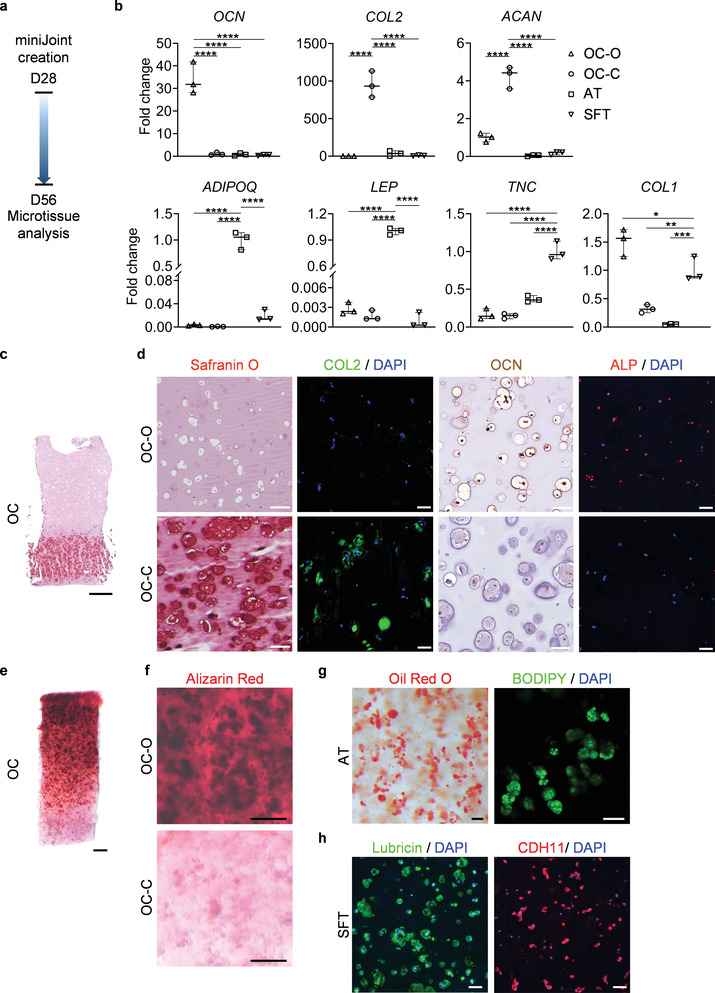
The microtissues maintained their individual tissue phenotypes after four weeks of co‐culture in the miniJoint chip. a) Timeline of generating a normal miniJoint chip with tissue‐specific phenotypes maintained. b) Box plots showing the expression of key marker genes in all four tissue components. *OCN* data were normalized to those in OC‐C, *COL2*, and *ACAN* data to values in OC‐O, *ADIPOQ* and *LEP* data to values in AT, and *TNC* and *COL1* data to values in SFT. Data were analyzed by one‐way ANOVA (*N* = 3 biological replicates). *, *p* < 0.05; **, *p* < 0.01; ***, *p* < 0.001; ****, *p* < 0.0001. c) Safranin O staining of the biphasic OC unit. Scale bar = 1 mm. d) Histological staining and immunostaining images confirming the presence of bone‐ and cartilage‐specific markers in the corresponding component of the OC unit. Scale bar = 50 µm. e) Alizarin Red staining of the OC unit showing the presence of calcium deposits primarily in the OC‐O. Scale bar = 500 µm. f) Magnified views of the Alizarin Red staining image in e). Scale bar = 200 µm. g) Oil Red O and BODIPY staining images showing the retention of lipid droplets in AT. Scale bar = 50 µm. h) Immunostaining images showing the expression of lubricin and CDH11 by the synovial‐like fibrous tissue (SFT). Scale bar = 50 µm.

A general challenge of a multicomponent coculture is how to maintain the phenotypes of the individual tissue components. After 4 weeks, individual tissue modules in the miniJoint were collected and their phenotypes analyzed (**Figure** **3**a). RT‐qPCR, histological staining, and immunoassays all confirmed that the individual microtissues displayed well‐maintained tissue‐specific phenotypes (Figure 3b–h; and Table S3, Supporting Information). For example, *OCN* was highly expressed only in OC‐O, but not in the other three microtissues, while high expression levels of *COL2* and *ACAN* were observed only in OC‐C (Figure 3b). OCN and ALP proteins were predominantly observed in OC‐O, and GAG and COL2 were retained in OC‐C (Figure 3c,d; and Figure S5, Supporting Information). Alizarin Red staining of the OC unit showed calcium deposition primarily in OC‐O (Figure 3e,f). No GAG deposition was observed in the SFT (Figure S11, Supporting Information). In addition, the levels of collagen type X (COL10) and Indian hedgehog (IHH), two hypertrophy markersobserved in OC‐C microtissue on D28, were found to be significantly reduced after 4 weeks of miniJoint culture with SM supplemented with 0.5 ng mL^−1^ TGF*β*3 (Figure S12, Supporting Information). Immunostaining showed no expression of COL1 in OC‐C, while this fibrous marker protein was maintained at a high level in SFT (Figure S11, Supporting Information). AT and SFT were also able to maintain their respective phenotypes, as revealed by RT‐qPCR, histology and immunostaining (Figure 3b,g,h). Collectively, these results confirmed the successful establishment of a multi‐tissue human joint model in the miniJoint chip.

### 2.3 Simulating Inflammatory Features in miniJoint

**Figure 4:**
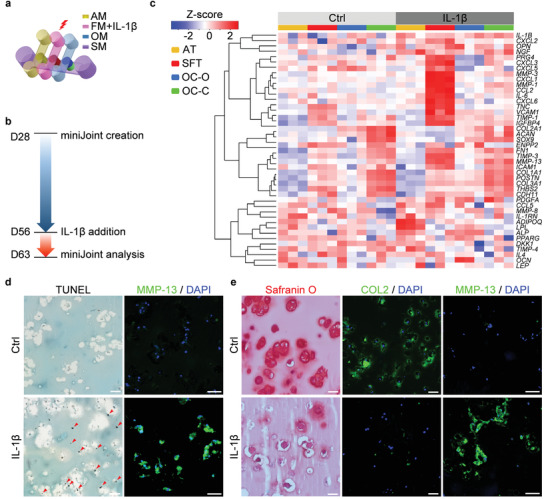
Generation and characterization of an inflamed joint model in the miniJoint chip. a) The inflamed joint model was created by challenging the SFT via the addition of IL‐1*β* to the fibrogenic medium (FM). AM, adipogenic medium; OM, osteogenic medium; SM, shared medium. b) Timeline of generating and analyzing the inflamed miniJoint. c) Heat map generated from RNA‐Seq showing the relative expression levels of selected genes in healthy (Ctrl) and inflamed (IL‐1*β*) miniJoint. AT: adipose tissue; SFT: synovial‐like fibrous tissue; OC‐O, bone component; OC‐C, cartilage component. *N* = 3 biological replicates. d) TUNEL assay and MMP‐13 immunostaining images confirming the generation of an inflamed SFT. Scale bar = 50 µm. Red arrowheads indicate DNA fragmentation. e) Histological staining and immunostaining of OC‐C showing cartilage degeneration. Scale bar = 50 µm.

With the successful generation of the normal, healthy miniJoint, we then tested its utility to model diseases of the joint. The unique multiflow design of the miniJoint allowed for the introduction of selected stimuli and therapeutics to individual tissues or to all tissues at once. By creating a disease state in one tissue, we would be able to determine its role(s) in disease pathologies by testing whether and how other surrounding tissues would be altered. Abundant evidence indicates that inflammation of the synovium (synovitis) is critical in the pathogenesis of joint diseases.^[^ ^12^ ^]^ We therefore used the proinflammatory cytokine IL‐1*β* to create “synovitis” and simulate joint inflammation in the miniJoint chip (**Figure** **4**a). Simulation results showed that IL‐1*β* could not significantly diffuse through the SFT into the SM (Figure S13, Supporting Information), which was also validated with an enzyme‐linked immunosorbent assay (ELISA with a sensitivity of 6.5 pg mL^−1^, data not shown here). As shown in Figure S14a (Supporting Information), 10 ng mL^−1^ IL‐1*β* treated fibroblasts displayed an inflammatory phenotype. Also, the conditioned medium collected from IL‐1*β* treated fibroblasts was able to induce the degeneration of engineered cartilage (Figure S14b, Supporting Information).

After validating the function of fibroblasts in responding to IL‐1*β*, we next created “synovitis” in the miniJoint and examined the influence of inflamed SFT on other tissues. As shown in Figure 4a, IL‐1*β* was introduced to the FM stream in the miniJoint to only stimulate SFT, while other tissues were not directly exposed to IL‐1*β* treatment. After 7 days, the viability of cells in SFT was evaluated by LIVE/DEAD and alamarBlue assays (Figure S15, Supporting Information). Although the superficial layer at the top showed more dead cells in the IL‐1*β*‐treated SFT when compared to the control group, the total metabolic activities remained comparable between the two groups. The health of different tissues was assessed on day 63 (Figure 4b). RNA‐Seq data showed that the number of differentially expressed genes (DEGs) was 790, 1839, 1376, and 568 for AT, SFT, OC‐O, and OC‐C, respectively (Table S4, Supporting Information). We then analyzed the expression levels of selected tissue‐specific marker genes, proinflammatory cytokines, matrix metalloproteinases (MMPs), and other degenerative markers in all four tissues that have been well studied in joint disease‐related investigations (Figure 4c, with the list of genes, *p*‐values, and adjusted *p*‐values provided in Table S5, Supporting Information).^[^ ^12^, ^13^ ^]^ Upregulated expression of major catabolic genes was detected within the SFT in the IL‐1*β*‐treated, inflamed miniJoint. In addition, OC‐C displayed reduced *ACAN* and *COL2* expression, and significantly upregulated *IL‐1B* as well as *MMP‐3* & *13* (Figure 4c), signifying inflammation and cartilage matrix degradation. Along with inflammation and degradation in OC‐C, altered expression of genes associated with pathologic changes was also observed in OC‐O and AT microtissues. Compared to the healthy control, the inflamed miniJoint chip showed increased expression of *ADIPOQ* and lipoprotein lipase (*LPL*) in AD. Interestingly, enhanced expression of *MMP‐13* was concomitant with the upregulation of expression of osteopontin (*OPN*) and *ALP* in OC‐O in the inflamed miniJoint, suggesting a possible remodeling process. The tissues in the inflamed miniJoint showed consistently upregulated expression of nerve growth factor (NGF), a key mediator of the sensation of joint pain.^[^ ^14^ ^]^

Next, we examined tissue phenotypes by histology and immunostaining and analyzed how other tissues responded to the inflamed SFT. Terminal deoxynucleotidyl transferase dUTP nick end labeling (TUNEL) assay revealed a considerably higher level of DNA fragmentation in IL‐1*β* treated SFT, indicating an increase in the number of apoptotic cells (Figure 4d). The IL‐1*β* challenged SFT also generated a considerably higher level of MMP‐13 than the control (Figure 4d; and Figure S5, Supporting Information). Cartilage degeneration was observed in the inflamed miniJoint, as confirmed by reduced GAG retention, lowered COL2 protein level, and higher MMP‐13 expression via histological staining and immunofluorescence (Figure 4e; and Figure S5, Supporting Information).

**Figure 5:**
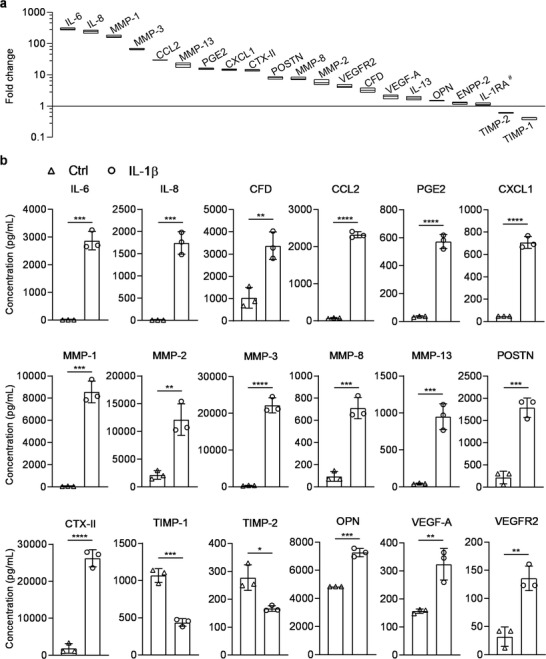
Levels of selected inflammation and degeneration markers in the SM collected from untreated (Ctrl) or inflamed (IL‐1*β*) miniJoint. a) Fold change in the concentrations of selected markers present in SM from the inflamed (IL‐1*β*) miniJoint, normalized to the levels of corresponding markers in SM from the Ctrl group (i.e., values for the Ctrl group were set at 1). Markers with # showed no statistical difference in concentration between the inflamed miniJoint and the untreated control (*p* ≥ 0.05). All others without # indicated statistical difference (*p* < 0.05). Data were analyzed by the Student's *t*‐test (*N* = 3 biological replicates). The box limits indicate the minimum and maximum values, with the line inside denoting the median. b) The concentrations of selected molecules in the SM collected from Ctrl and inflamed miniJoint chips. Data were analyzed by the Student's *t*‐test (*N* = 3 biological replicates). *, *p* < 0.05; **, *p* < 0.01; ***, *p* < 0.001; ****, *p* < 0.0001. SM, shared medium.

In the design of the miniJoint, the crosstalk between SFT and other tissues was mediated primarily through the SM; we therefore measured the levels of selected biomarkers in SM using Luminex immunoassay and ELISA (for targets not available in Luminex) (**Figure** **5**a,b). The SM collected from the inflamed miniJoint showed significantly higher concentrations of inflammatory markers, such as prostaglandin E2 (PGE2), interleukin 6 (IL‐6), IL‐8, and IL‐13, and degenerative enzymes, MMP‐1, MMP‐3, MMP‐8, and MMP‐13, compared to the control group. The levels of inflammation‐modulatory cytokines, including chemokine (C‐X‐C motif) ligand 1 (CXCL1), and C‐C motif chemokine ligand 2 (CCL2), also noticeably increased in the SM of inflamed miniJoint. Interestingly, the cartilage degradation product C‐telopeptide of collagen type II (CTX‐II), osteoblast‐related markers periostin (POSTN) and OPN, and the adipokine adipsin (complement factor D; CFD) were all significantly higher in SFF collected from the inflamed miniJoint than the control (Figure 5b). Vascular endothelial growth factor‐alpha (VEGFA) and VEGF receptor 2 (VEGFR2) were also present at much higher concentrations in the SM of inflamed miniJoint. Furthermore, we observed decreased concentrations of tissue inhibitor of metalloproteinase (TIMP)‐1 and TIMP‐2 in the SM of inflamed miniJoint.

Taken together, these data supported the successful generation of inflammatory and degenerative features of joint diseases in the miniJoint. The results also demonstrated that OC and AT microtissues were responding to the pathological changes of SFT, implying the active crosstalk within miniJoint.

**Figure 6:**
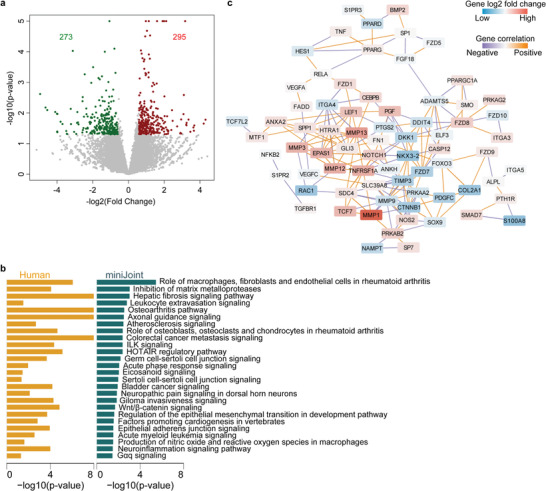
Transcriptome analysis of the cartilage component in the inflamed miniJoint and comparison with the transcriptome of cartilage collected from human OA patients.^[^ ^15^ ^]^ a) Volcano plot showing 273 downregualted (in green) and 295 upregulated (in red) genes expressed in the cartilage tissue component (OC‐C) from control (Ctrl) and inflamed (IL‐1*β*) miniJoint. b) Common, significantly enriched pathways between OC‐C of the inflamed miniJoint and cartilage tissue from human OA patients. c) Network plot for DEGs in Osteoarthritis pathway in OC‐C of IL‐1*β* treated miniJoint.

To further assess the fidelity of miniJoint in modeling human joint diseases, we employed RNA‐Seq to examine whether the inflamed miniJoint was able to recapitulate the changes observed in human OA samples. Since RNA‐Seq has primarily been conducted on human cartilage samples collected from healthy or OA patients, we compared our RNA‐Seq data generated from OC‐C in miniJoint to those reported in a most recent study GSE114007.^[^ ^15^ ^]^ As shown in Figure**6a** **;** and Table S4 (Supporting Information), 295 genes were found to be upregulated and 273 genes were downregulated in OC‐C from the inflamed miniJoint. The recent differential expression analysis performed on articular cartilage isolated from OA patients independently showed that expression levels of 2121 genes were decreased and 1790 were increased.^[^ ^15^ ^]^ We next performed pathway enrichment analysis based on the DEGs in both miniJoint (Figure S16 and Table S6, Supporting Information) and human studies, and determined that 26 pathways were found to be common in both systems (Figure 6b). In particular, the OA pathway was identified in OC‐C of the inflamed miniJoint and in articular cartilage from OA donors. Interestingly, OC‐C in the inflamed miniJoint seemed to share more significantly enriched pathways with human OA cartilage than the cartilage tissue of a mouse model (Figure S17, Supporting Information). Furthermore, when genes that are included in the OA pathways were examined (Figure 6c), it was found that the inflamed miniJoint system could recapitulate many changes in gene expression that have been observed in human knee joint diseases, such as increased expression of MMPs and decreased expression of chondrogenic genes.

To further verify the critical role of the inflamed SFT in OC‐C degradation observed, miniJoint chips with normal SFT or acellular inserts were assembled and insulted with IL‐1*β* (10 ng mL^−1^) introduced in the FM stream. Comparative evaluations of OC‐C microtissues harvested from the challenged chips and the healthy (no IL‐1*β* insult) control were carried out by RNA‐Seq (Figure S18, Supporting Information). The Principal Component Analysis (PCA) plot and heatmap of sample‐to‐sample distances (Figure S18a,b, Supporting Information) show that after IL‐1*β* treatment, the chips with acellular SFT showed high similarity to the healthy control, while those with cell‐laden SFT showed obvious differences than the other two groups. Furthermore, replacing a cell‐laden SFT with an acellular insert seemed to abolish IL‐1*β* induced OC‐C degeneration (Figure S18c, Supporting Information), causing a 3.8‐fold and 6.2‐fold decrease in the number of downregulated and upregulated genes, respectively, in the OC‐C microtissue under IL‐1*β* insult (Figure S18d and Table S7, Supporting Information). Taken together, these data supports the key role of SFT inflammation in inducing OC‐C degeneration in the established disease model and rule out IL‐1*β* leakage as a driver of OC‐C degeneration.

### 2.4 Testing the “Systemic Therapeutic Effect” of Naproxen in miniJoint

**Figure 7:**
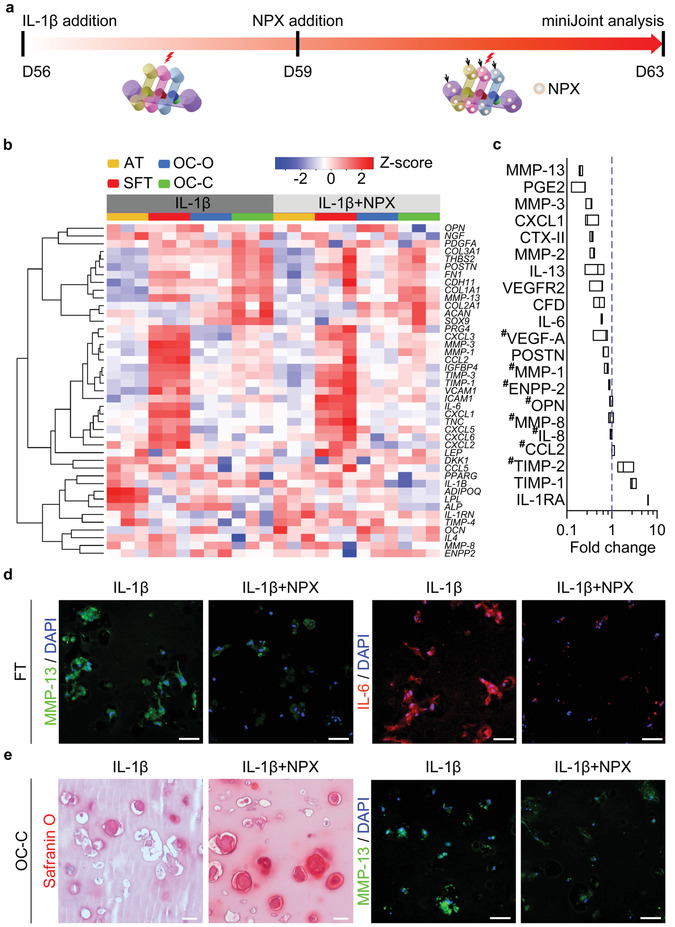
Testing the “therapeutic efficacy” of naproxen (NPX) in the inflamed miniJoint. a) Schematic and timeline of “systemic” administration of NPX in miniJoint (analogous to enteral/parenteral administration in vivo). NPX was added to all the medium streams (indicated by black arrows) after 3 days of IL‐1*β* treatment of the SFT tissue. b) Heat map generated from RNA‐Seq showing the relative expression of selected marker genes in all four tissues. c) Levels of selected biomarkers in SM, collected from inflamed miniJoint without (IL‐1*β*) or with NPX treatment (IL‐1*β*+NPX). The concentration of each marker was normalized to that in the non‐NPX‐treated IL‐1*β* group, with # indicating no statistical difference between the two groups (*p* ≥ 0.05). Data were analyzed by the Student's *t*‐test (*N* = 3 biological replicates). The box limits indicate the minimum and maximum values, with the line inside denoting the median. d) Immunostaining images showing reduced levels of MMP‐13 and IL‐6 in the SFT microtissue after NPX treatment. Scale bar = 50 µm. e) Safranin O staining and immunostaining showing more GAG retention and lower MMP‐13 level in OC‐C, respectively, after NPX treatment. Scale bar = 50 µm.

To validate the utility of the miniJoint chip as a platform for drug screening, it is important to assess the impact of agents with known clinical efficacy. Since there are no DMOADs approved by regulatory bodies, naproxen (NPX), a nonsteroidal anti‐inflammatory drug (NSAID) commonly prescribed to OA patients, was selected for the study. NPX was administered into all streams of the inflammatory miniJoint at 5 × 10^−6^ m for four days to test its “therapeutic effect” (Figure 7a). RNA‐Seq results revealed 1436, 799, 945, and 746 DEGs in AT, SFT, OC‐O, and OC‐C, respectively, of the NPX‐treated miniJoint (Table S8, Supporting Information). The changes in expression of selected marker genes in all four tissues were shown in Figure 7b and Table S9 (with *p*‐values and adjusted *p*‐values included). NPX treatment was found to effectively mitigate the inflammation induced by IL‐1*β* treated SFT. For example, the expression levels of *MMP‐1*, *3* and *13* in SFT were found to be downregulated after NPX treatment (Figure 7b), which were further confirmed by immunofluorescence staining (Figure 7d and Figure S5, Supporting Information). At the same time, safranin O staining showed more GAG retention in the NPX‐treated OC‐C than in the OC‐C in the inflamed miniJoint (Figure 7e), indicating the efficacy of NPX in suppressing cartilage degeneration. A markedly reduced presence of MMP‐13 was observed in the NPX‐treated OC‐C microtissue, as revealed by immunofluorescence (Figure 7e and Figure S5, Supporting Information), although the expression level of tested genes in OC‐C was not significantly changed (Figure 7b). NPX treatment also decreased the expression of *IL‐1B*, *OPN* and *NGF* in OC‐C. Interestingly, all microtissues showed upregulated expression of IL‐1 receptor antagonist (*IL‐1RN*), a gene that encodes IL‐1RA that can block the binding of IL‐1 to its receptor. NPX treatment also reduced the expression of *ADIPQ* and *LPL* in AD, as well as suppressed *ALP* expression in OC‐O.

Furthermore, analysis of the SM revealed that, upon NPX treatment, concentrations of MMP‐1, MMP‐2, MMP‐3, MMP‐13, IL‐6, IL‐13, PGE‐2, CTX‐II, POSTN, CXCL‐1, and VEGFR2 were lower, compared to the control inflamed miniJoint (Figure 7c). In addition, the NPX‐treated miniJoint displayed higher concentrations of TIMP‐1 and IL‐1RA in the SM than the control group. The addition of NPX to the inflamed miniJoint did not alter the concentrations of CCL2, MMP‐8, IL‐8, or OPN in the SM.

### 2.5 Evaluating the Efficacy of “Intraarticular Administration” of Potential DMOADs in miniJoint

**Figure 8:**
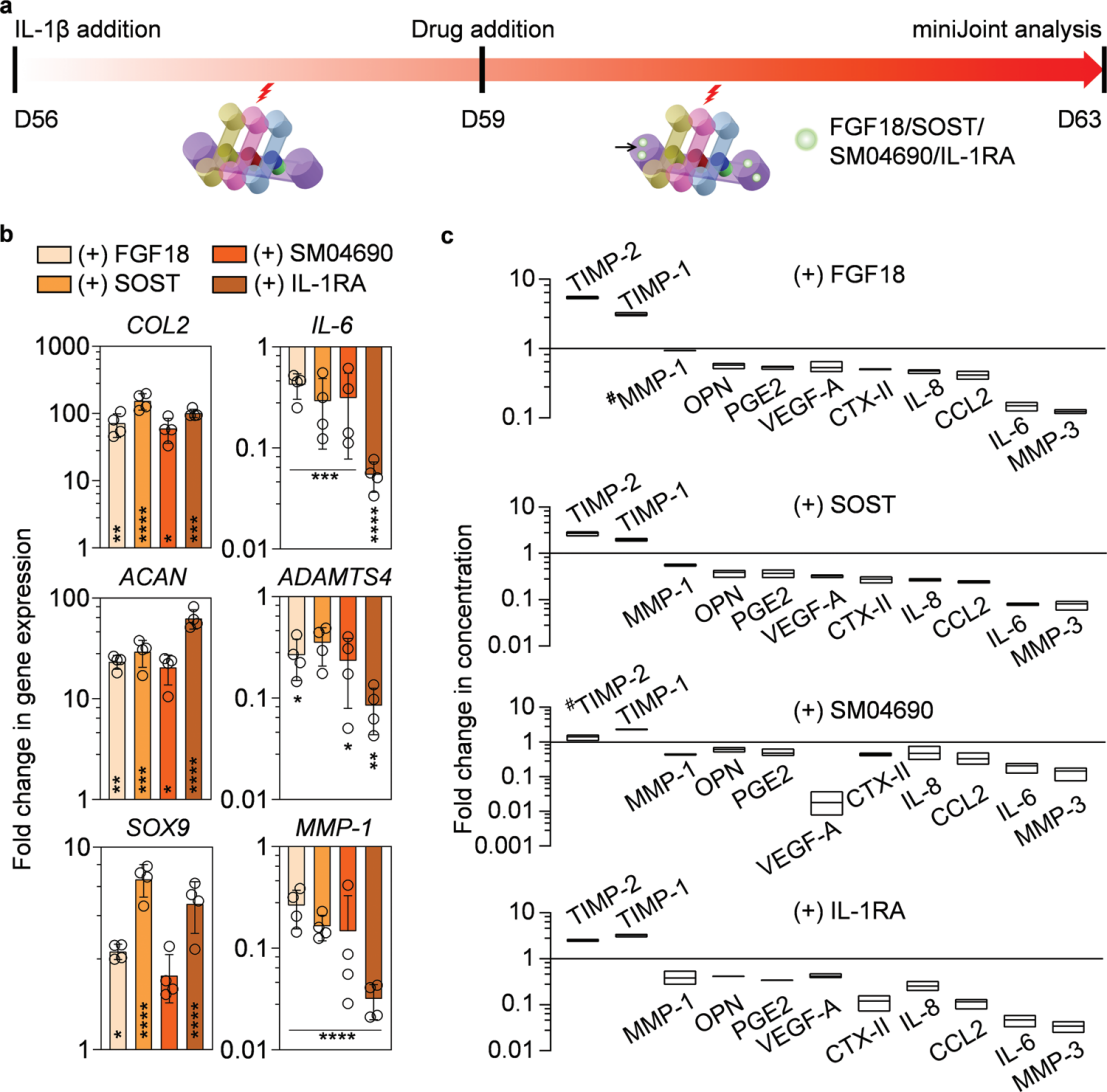
Evaluating four potential DMOADs via “intraarticular administration” in the inflamed miniJoint. a) Schematic and timeline of intraarticular administration of drugs. Each drug was added to the shared medium stream (indicted by black arrow) after 3 days of IL‐1*β* treatment of the SFT tissue. b) Gene expression in OC‐C after 4 days of drug treatment. Gene expression data were normalized to those of the nondrug‐treated group. Data were analyzed by the Student's *t*‐test (*N* = 4 biological replicates). Statistical differences between each drug‐treated group and the nondrug‐treated group are indicated by *, *p* < 0.05; **, *p* < 0.01; ***, *p* < 0.001; and ****, *p* < 0.0001. *N* = 4 biological replicates. SOX9: SRY‐Box Transcription Factor 9. c) Levels of selected biomarkers in SM, collected from inflamed miniJoint without or with 4 days of FGF18, SOST, SM04690, or IL‐1RA treatment. The concentration of each marker was normalized to that in the nondrug‐treated group, with # indicating no statistical difference in marker concentration between the two groups (*p* ≥ 0.05). All others without # indicated statistical difference (*p* < 0.05). Data were analyzed by the Student's *t*‐test (*N* = 3 biological replicates). The box limits indicate the minimum and maximum values, with the line inside denoting the median.

We further assessed the utility of miniJoint for pharmacological evaluation by testing four representative DMOADs that are under development, including fibroblast growth factor 18 (FGF18 or Sprifermin; 100 ng mL^−1^), SM04690 (Lorecivivint; 1 × 10^−8^ m), sclerostin (SOST; 250 ng mL^−1^), and IL‐1RA (250 ng mL^−1^). To simulate the primarily intra‐articular application of these drugs in clinical trials, they were added only to the SM **(Figure** **8a** **)**. It was found that all the drugs tested resulted in statistically significant upregulation of chondrogenic genes, including *COL2* and *ACAN*. In addition, these drugs also induced significant downregulation of gene expression of the inflammatory marker *IL‐6* as well as the degenerative marker a disintegrin and metalloproteinase with thrombospondin motifs 4 (*ADAMTS4*), a gene that encodes aggrecan‐degrading enzyme (Figure 8b).

We then measured the concentrations of selected SM biomarkers associated with joint health and degeneration. Compared with the SM from the non‐drug‐treated, inflamed miniJoint, those from the drug‐treated groups generally displayed higher concentrations of TIMP‐1 and TIMP‐2, as well as lower concentrations of a number of degenerative and inflammatory markers (Figure 8c). Also, it was noted that FGF18 was not able to reduce the level of MMP‐1 in SM.

## 3 Discussion

Diseases of knee joints such as OA significantly limit mobility and reduce the quality of life for patients. Anti‐inflammatory drugs such as NSAIDs can alleviate inflammation and ease the pain for some patients, but they eventually lose their efficacy.^[^ ^16^ ^]^ Drugs capable of modifying disease progression are therefore critically needed. However, a recent study by Malfait et al. concluded that preclinical OA models were poor predictors of the outcomes of clinical trials.^[^ ^17^ ^]^ Therefore, we aimed to produce a new miniature in vitro human knee joint model as an alternative system that effectively stimulates key joint disease features, and thus also aids in drug treatment advancement. While the “knee joint‐on‐a‐chip” concept has been coined,^[^ ^5^, ^7^ ^]^ the successful generation of such systems has not yet been reported.

Like the development of other tissue/organ chips,^[^ ^9^, ^18^ ^]^ engineering a knee joint identical to the native counterpart is impractical and unnecessary. The key is to recapitulate select essential aspects of the organ physiology and diseases, and then use this model to inform the screening for drug toxicity and efficacy in humans.^[^ ^18b^ ^]^ In 2014, we constructed, for the first time, an osteochondral tissue chip derived from hBMSCs, using a similar device to that described in Figure 2b. Although concomitant hypertrophy is a major disadvantage of hBMSC‐derived cartilage,^[^ ^19^ ^]^ this hypertrophic state, however, is transient and highly dependent on the presence of a high TGF level (typically 10 ng mL^−1^ in the chondrogenic medium). Therefore, when cultured in the miniJoint chip (day 28–56), the OC‐C was maintained in a medium with a low level of TGF (0.5 ng mL^−1^), which significantly decreased the level of hypertrophy in OC‐C, as supported by the immunostaining results in Figure S12 (Supporting Information). The utility of our original chip was recently expanded by using induced pluripotent stem cells (iPSCs) as the constituent cell source.^[^ ^8b^ ^]^ Following the osteochondral tissue chip's success, the next phase was to incorporate additional joint tissues found within the knee joint. To determine which tissues would be included in our study, two primary joint diseases were considered, OA and rheumatoid arthritis (RA). In RA, synovium inflammation, also called synovitis, is well known as a significant disease contributor.^[^ ^12c^ ^]^ Recently, synovitis has been found to be an active component in OA pathology as well.^[^ ^12a^ ^]^ Therefore, we first elected to include the synovium in addition to the osteochondral tissue. The selection of adipose tissue as the fourth miniJoint element was inspired by the association between obesity and OA, and the contribution of adipose tissue to joint diseases was further shown in several recent animal studies.^[^ ^20^ ^]^ Adipose tissue produces adipokines as well as major inflammatory cytokines such as IL‐1*β* and IL‐6.^[^ ^21^ ^]^ Serum levels of these cytokines are elevated in OA patients, suggesting that metabolic disruption of adipose tissue may play a role in OA severity and progression.^[^ ^22^ ^]^ The infrapatellar fat pad (IPFP), an anatomically unique adipose tissue located in the knee joint, has been shown to undergo changes during the pathogenesis of OA.^[^ ^23^ ^]^ However, the exact role of adipose tissue in OA disease progression requires further investigation. While synovium and adipose tissue represent promising additions to our study, other joint tissues, including muscle, meniscus, and intraarticular ligament, also contribute to normal joint function and physiology. These tissues have major biomechanical functions and their injuries caused by abnormal mechanical loading can eventually impact cartilage and bone and lead to joint diseases.^[^ ^24^ ^]^ These tissues may be included in the future development of joint organ chips.

Although primary cells (osteoblasts, chondrocytes, adipocytes, and fibroblasts) can be employed to create a joint‐mimicking chip, this approach requires several procedures to collect different tissues to isolate these cells. Also, the relatively low availability and limited expansion potential of primary cells are not compatible with the miniJoint system since it requires many cells. In comparison, MSCs can be extensively expanded and differentiated into four tissue types, which has been shown in the current study. Moreover, another advantage of using MSCs is that the different tissue components are derived from the same starting cell source, whereas the use of primary cell sources or established primary cell lines will almost necessarily involve cells from different individuals, thus presenting potential incompatibilities. Immortal MSC lines, such as human telomerase immortalized cells,^[^ ^25^ ^]^ are also potential cell sources to create the miniJoint in the future.

In the synovium, there are two major types of cells: fibroblast‐like and macrophage‐like synoviocytes.^[^ ^26^ ^]^ Of note, there are no exclusive markers to identify fibroblast‐like synoviocytes. Therefore, we examined several molecules that have been previously shown to be expressed in synovial fibroblasts, including COL1, lubricin, CDH11, *β*1 integrin, and CD44.^[^ ^27^ ^]^ In this research, as well as a recent study on synovium‐cartilage chip,^[^ ^28^ ^]^ macrophage‐like synoviocytes were not included in the SFT; instead, their function was partially realized through IL‐1*β* treatment. Due to the challenges in 3D culture, especially the requirement of long‐term culturing, macrophages are rarely included in current organ‐on‐a‐chip models.^[^ ^29^ ^]^ In our previous studies, GelMA‐encapsulated macrophages that were cultured for 1, 7, and 14 days, were subjected to M1 or M2 polarization. We found that macrophage viability and bioactivity decreased with increased culture time.^[^ ^30^ ^]^ The short life expectancy of macrophages in 3D culture was also observed in another study.^[^ ^31^ ^]^ To overcome the lack of macrophages in SFT, we used IL‐1*β* to stimulate the inflammatory component (Figure 4). Nevertheless, it is technically feasible to engineer macrophage‐containing SFT which can be used for short‐term cultures in the miniJoint. Our preliminary data show that M1 macrophages co‐encapsulated with fibroblasts in SFT microtissues in a dual‐flow bioreactor can secret high levels of IL‐6 for at least 14 days (Figure S19, Supporting Information). Our ongoing studies include the development of a protocol that extends macrophage viability and potential in dynamic, 3D cultures.

With the successful generation of fibrous tissues, it is now possible to shift from an osteochondral chip to a multi‐component knee joint model that can interrogate molecular and cellular mechanisms involved in joint health and disease. The novel design of the miniJoint bioreactor allows the assembly of these four tissues in diverse arrangements. In this study, we first adopted an order observed in the native joint, in which the synovium is between the fat pad and OC. The SM was introduced to mediate the “local” crosstalk among OC‐C, AT, and SFT. It should be noted that by connecting the top three flows, we can conveniently mimic the “systemic fluid circulation.” To balance the rapid nutrient depletion in static culture and insufficient tissue crosstalk in dynamic culture, we selected a hybrid flow pattern in SM. A future option to increase SM‐mediated tissue crosstalk would use a recirculation model that perfuses downstream medium back to the upstream.

While IL‐1*β* has been the most commonly used agent to model inflammatory joint diseases in vitro, direct IL‐1*β* introduction into SM assaults the three tissues (OC‐C, bottom parts of AT and SFT) at the same time, which may mask the effects of potential tissue crosstalk. In addition, as previously described, synovitis is recognized as an important OA and RA feature. In particular, prior synovitis, identified by magnetic resonance imaging, was significantly associated with subsequent OA development,^[^ ^32^ ^]^ suggesting that synovitis is likely a precursor to radiographic OA. The association between synovitis and further OA progression has also been demonstrated.^[^ ^12a^ ^]^ Therefore, we simulated a “synovitis” model in miniJoint by challenging only SFT with IL‐1*β* (10 ng/ml), without directly exposing other tissues to exogenous IL‐1*β*. In concordance with our results in Figure 4, increased expression of *MMP‐13* and *IL‐8* was also observed in the mechanical overloading‐induced OA model established in cartilage‐on‐a‐chip by Occhetta et al.^[^ ^9^ ^]^ Interestingly, we found that the changes in the expression of *COL2, ACAN, MMP‐1* and *MMP‐13* in OC‐C between the inflamed miniJoint and the healthy control observed here (Figure S20, Supporting Information) in fact more closely resemble reported changes between OA and normal human articular cartilage than our previously developed osteochondral tissue chip, which lacks AT and SFT components.^[^ ^8^, ^33^ ^]^ Specifically, the *COL2, ACAN, MMP‐1*, and *MMP‐13* expression levels showed 4.5‐fold decrease, 3.4‐fold decrease, 18.7‐fold increase, and 18.4‐fold increase, respectively, for the OC‐C of the inflamed miniJoint (Figure S20, Supporting Information). These fold change values were found to be much closer to those reported for human OA cartilage,^[^ ^33^ ^]^ as compared to the values obtained from the osteochondral tissue chip.^[^ ^8a^ ^]^ To further assess the miniJoint's potential in modeling human OA, we analyzed the transcriptomic changes in OC‐C from the control and inflamed miniJoint, and then compared the alterations to those recently reported in a study using human samples.^[^ ^15^ ^]^ As shown in Figure 6b, 26 common pathways were found between OC‐C from the inflamed miniJoint and human OA cartilage. We also analyzed the RNA‐Seq data from another human study GSE57218,^[^ ^34^ ^]^ and an animal study GSE143447,^[^ ^35^ ^]^ in which the OA model was created through destabilization of the medial meniscus (DMM). As shown in Figure S17 (Supporting Information), miniJoint seemed to share more common pathways with human samples than animal models and displayed more changes that are associated with OA. It should be noted that currently no single in vitro or in vivo models can mimic all features of joint diseases in humans;^[^ ^5^ ^]^ rather, each model recapitulates certain aspect(s) of the pathological changes. The miniJoint developed here can be used to increase the efficiency in selecting drug candidates for large animal studies or clinical trials.

In order to predict the appropriate drug interventions and treatments needed in humans, specific and sensitive biomarkers are critical. In the native joint, synovial fluid bathes all intrinsic synovial joint structures, and provides a wealth of information on potential diagnostic and prognostic OA markers.^[^ ^20^, ^36^ ^]^ The Luminex immunoassay employed here allowed us to analyze the known potential biomarkers in a medium‐throughput manner. The SM from the inflammatory miniJoint contained many biomarkers well recognized in the synovial fluid of OA patients,^[^ ^37^ ^]^ including proinflammatory cytokines, angiogenic factors, and MMPs. In addition, we have found increased CCL2,^[^ ^38^ ^]^ OPN,^[^ ^36a^ ^]^ CFD,^[^ ^39^ ^]^ and POSTN levels,^[^ ^36b^ ^]^ which have been newly recognized as OA biomarkers in synovial fluid. The broad array of molecules detected in the SM signifies active involvement of all microtissues, as a result of their crosstalk, in the pathological changes in the inflamed miniJoint. By comparatively analyzing the biomarkers identified by methods such as Luminex and mass spectrometry^[^ ^40^ ^]^ for miniJoint SM and human synovial fluid, valuable information can be obtained to evaluate the clinical relevance of our miniJoint system. In the current study, we only measured the concentrations of biomarkers in the SM, not the other three streams. The concern was that the use of fetal bovine serum in these media (Table S1, Supporting Information) might complicate the interpretation of results or mask the changes of some markers. However, analyzing these media in the future provides the opportunity of identifying new disease biomarkers.

To further validate the miniJoint as a model system to predict the efficacy of novel therapeutic interventions in humans, it is essential to assess the impact of agents with known clinical efficacy on the miniJoint. NPX is one of the most commonly used NSAIDs to reduce synovitis and pain in patients with knee OA.^[^ ^41^ ^]^ The results in Figure 7 indicated and confirmed the anti‐inflammatory function of NPX. Of particular interest is the upregulation of *IL‐1RN* after NPX treatment in all microtissues (Figure 7b). Previous studies have identified *IL‐1RN* as a potential predictive biomarker for OA development;^[^ ^42^ ^]^ intraarticular delivery of IL‐1RA was found to be effective in decreasing cartilage degeneration and reducing severity of synovitis in a mouse model.^[^ ^43^ ^]^

The effect of NPX on human cartilage and chondrocyte phenotypes in the context of knee joint remains unclear. In the study by Mastbergen et al.,^[^ ^44^ ^]^ human OA cartilage was exposed to NPX treatment, and no changes were found in any measured parameters, such as cartilage proteoglycan turnover and PGE2 production. While NPX may inhibit inflammation, as confirmed by Dingle et al.,^[^ ^45^ ^]^ it does not have a beneficial effect on chondrocyte synthetic activity. In our study, we observed chondroprotective effects of NPX treatment using histological staining (Figure 7e), which agrees with a recent study where NPX treatment was found to reduce the loss of articular cartilage in the rat DMM model.^[^ ^46^ ^]^ However, the transcriptome in chondrocytes was not significantly influenced by NPX, supporting the findings by Mastbergen et al.^[^ ^44^ ^]^ Instead of playing a direct role on chondrocytes, naproxen may function through suppressing the inflammation of “synovial tissue” in the miniJoint and reducing the release of pro‐inflammatory cytokines and degradative enzymes to the SM (Figure 7c). Consequently, less cartilage degradation was observed. In fact, this is another advantage of using miniJoint in studying tissue crosstalk in drug testing. It is also possible that, instead of influencing transcription, the beneficial effects of NPX may be mediated by alterations in the degradation profile of the cartilage matrix, as supported by the reduced MMP‐13 protein level (Figure 7e; and Figure S5, Supporting Information).

The four potential DMOADs tested in this study have garnered much research interest and three of them (FGF18, SM04690 and IL‐1RA) are being tested in human clinical trials for OA treatment.^[^ ^47^ ^]^ It was found in previous studies that FGF18 could stimulate the production of cartilage extracellular matrix by articular chondrocytes in vitro and facilitate cartilage repair in a rat OA model.^[^ ^48^ ^]^ In this study, we found that FGF18 may also suppress the expression of catabolic genes in cartilage (Figure 8b,c). The small molecule SM04690 and SOST protein are both inhibitors of the Wnt signaling pathway, the inhibition of which has been found to ameliorate OA in a mouse model.^[^ ^49^ ^]^ IL‐1RA, the receptor antagonist of IL‐1, inhibits IL‐1 signaling and suppresses IL‐1‐induced tissue catabolism. Interestingly, it was reported that the beneficial effects of IL‐1RA were enhanced in co‐cultured cartilage and synovium than in cartilage monoculture.^[^ ^50^ ^]^ The drug testing results in Figure 8 agree with a number of previous studies and support the efficacy of these potential DMOADs in treating inflammatory, degenerative joint disorders. We believe that the novel multi‐tissue, human cell‐derived in vitro system developed here can be readily applied in studying the mechanisms underlying the therapeutic effects of potential drugs that target joint diseases. Of note, although all compounds were tested only in the inflamed miniJoint in the current study, they can also be administered in normal miniJoint not insulted by IL‐1*β* to understand their effects on healthy joint tissues in future studies.

## 4 Conclusion

We described the engineering of the miniJoint, the first human cell‐derived, multi‐tissue chip with the capacity to mimic both healthy and inflamed knee joints. The miniJoint chip incorporates osteochondral, fibrous, and adipose microtissues, and exhibited physiologically relevant pathological changes when the fibrous tissue experienced IL‐1*β* simulated inflammation. Furthermore, the potential of miniJoint in assessing and predicting in vivo efficacy of drug treatment was demonstrated by simulating the systemic or intraarticular administration of five drugs that are clinically used or under development for treating joint disorders.

The clinical relevance of the miniJoint can be further enhanced in the future. First, a mechanically active mechanism may be included in the miniJoint to simulate the physiological weight‐bearing function of the knee joint more effectively. In addition to the direct application of force, mechanical loading can also be partially simulated by directly modulating key mechanotransduction pathways, such as the transient receptor potential cation channel subfamily V member‐4 (TRPV4) pathway, a known mechanosensitive downstream signaling pathway.^[^ ^51^ ^]^ Second, in this study we have used the same hydrogel scaffold to derive all microtissues. However, GelMA‐based scaffold with the same stiffness may not be the best option to engineer all miniJoint tissues with desired phenotypes. To further optimize the microtissues, we will explore hydrogels with different mechanical and microstructural properties in the future. The degradability of the scaffold material will also be optimized to facilitate the deposition of cell‐secreted nascent matrix and the replacement of the original, synthetic matrix. Third, although inflammation in the miniJoint was realized or simulated by adding IL‐1*β* into FM, immune cells such as macrophages have not been included in the miniJoint. As described above, the immune cells can be introduced through including an additional module. Finally, we will create different types of joint disease models in the context of the miniJoint by introducing different stimuli, and test a broad range of drugs to validate the miniJoint's clinical relevance in predicting drug toxicity and efficacy in humans.

## 5 Experimental Section

### Manufacturing miniJoint Chip Components

To design the miniJoint bioreactor 3D models, SolidWorks 2018 software (Dassault Systèmes SE, Vélizy‐Villacoublay, France) was used. Each bioreactor part, including the chambers, caps, and inserts, was then additively manufactured by stereolithography using the E‐Shell 450 photopolymer ink and a Vida desktop 3D Printer (EnvisionTec, Dearborn, MI). The hollow inserts, with a height of 4.8 mm and inner diameter of 3.5 mm, housed the cell‐laden hydrogels, generating modular microtissues (Figure 1b). Surface grooves on the inserts and lids were designed to retain silicone O‐rings (McMaster‐Carr, Elmhurst, IL) that ensure tight seal in the miniJoint. Dual‐flow osteochondral (OC) chips, used to generate osteochondral microtissues in the first 28 days, were similarly designed and manufactured (Figure 2c). All 3D printed parts were sterilized with ethylene oxide gas and rinsed a few times with sterile phosphate‐buffered saline (PBS; Gibco, Grand Island, NY) before cell culture use.

### Engineering Microtissue Modules

Different miniJoint tissue components, including OC, AT, and SFT, were generated by differentiating human bone marrow‐derived stem cells (hBMSCs). hBMSCs were harvested from total joint arthroplasty surgical waste with IRB approval (University of Pittsburgh and University of Washington). To minimize donor‐to‐donor difference and obtain sufficient cells, hBMSCs were collected and combined from 20 donors aged 20–87 years old (Figure S1a, Supporting Information). The pooled MSCs were characterized using CFU assay (Figure S1b,c, Supporting Information), trilineage differentiation (Figure S1d, with compositions of induction media provided in Table S10, Supporting Information), and flow cytometry (Figure S1e, Supporting Information). The GelMA hydrogel and photoinitiator, lithium phenyl‐2,4,6‐trimethylbenzoylphosphinate (LAP), were synthesized as described in our previous study.^[^ ^52^ ^]^

hBMSCs at passage 5 (P5) were resuspended in 15% w/v GelMA at 20 million mL^−1^. To form the 3D microgel within the inserts, the cell suspension was pipetted into sterile inserts and cured in situ for 2 min with 395 nm visible light illumination. The formed 3D cell‐laden scaffolds bound tightly to the inner wall of the inserts, which together with the O‐ring, separate the top and bottom medium streams in miniJoint.

To generate OC microtissues, the inserts containing hBMSCs‐laden hydrogel were assembled in a dual‐flow OC chip, and osteogenic medium (OM) and chondrogenic medium (CM) were perfused through the top and bottom streams, respectively (OM and CM composition given in Table S1, Supporting Information).^[^ ^8^, ^53^ ^]^ A pulse flow pattern, consisting of 20s fast flow at 12 µL s^−1^ and 3580 s slow flow at 1/60 µL s^−1^, was provided by a programmable syringe pump (Lagato210P, KD Scientific, Holliston, MA). The OC chip culture was maintained for 4 weeks.

To create AT microtissue, inserts with hBMSCs‐laden hydrogel were cultured in AM (AM composition give in Table S1, Supporting Information) under static conditions for 4 weeks (Figure 1f).

To generate SFT microtissues, hBMSCs were expanded in growth medium (GM) over ≈5 days, trypsinized and plated in T150 flasks at a 2000 cells cm^−2^ density, and cultured in fibrogenic medium (FM) (FM composition give in Table S1, Supporting Information) for ≈3 weeks (Figure 1f). This incubation induced higher expression levels of fibrogenic genes, such as *COL1*, *TNC*, and *VCAN*, than 3D induction in the same medium (Figure S7b, Supporting Information). The fibroblast‐like cells derived from hBMSCs were then trypsinized (trypsin‐0.25% ethylenediaminetetraacetic acid; Gibco, Grand Island, NY), suspended in 15% w/v GelMA solution, and photocrosslinked to generate SFT microtissues. Tissue phenotype was analyzed by RT‐qPCR, histology, and immunostaining. Due to the lack of unique markers, the phenotypes of engineered SFT were assessed by examining several highly relevant molecules that were found in native synovium, including lubricin, CDH11, COL1, CD44, and β1 integrins.^[^ ^54^ ^]^

The reproducibility of the tissue phenotypes was confirmed by repeating each of the experiments two times.

### Real‐Time Polymerase Chain Reaction (RT‐qPCR)

Reverse transcription (Invitrogen, Carlsbad, CA) was first used to generate complementary DNA (cDNA) from the total RNA extracted from the microtissues (RNA extraction described in Supporting Information). The mixture of cDNA samples, forward and reverse primers (Integrated DNA Technologies, Inc., Coralville, Iowa; primer sequences provided in Table S11, Supporting Information), and SYBR Green (Bio‐Rad, Hercules, CA) was utilized to run RT‐qPCR on a CFX384 Touch RT‐PCR detection system (Bio‐Rad, Hercules, CA) or the QuantStudio 3 RT‐qPCR system (Applied Biosystems, Foster City, CA).

### Immunostaining

OC and SFT microtissues were formalin‐fixed, paraffin‐embedded (PPFE; Supporting Information), and 6 µm thick sections prepared. To assess tissue phenotypes, OC sections were incubated in antibodies against OCN (12.5 µg mL^−1^; MAB1419, R&D Systems, Minneapolis, MN), ALP (2 µg mL^−1^; ab224335, Abcam, Cambridge, MA), COL2 (1:200 dilution; ab34712, Abcam), and SFT sections in antibodies against lubricin (1:500 dilution; MABT401, MilliporeSigma, Burlington, MA) and CDH11 (2.5 µg mL^−1^; 71–7600, Invitrogen, Waltham, MA), overnight at 4 °C. Antibodies against MMP‐13 (1:200 dilution; ab39012, Abcam) and IL‐6 (1:200 dilution; PA1‐26811, Invitrogen) were used to examine inflammatory responses by the microtissues. Antigen retrieval was carried out using a 30 min incubation in a combined chondroitinase (9.6 µg mL^−1^; Sigma, St. Louis, MO) and hyaluronidase (6.25 mg mL^−1^; Sigma, St. Louis, MO) solution at 37 °C for COL2 and MMP‐13, and by heating the sections bathed in sodium citrate solution (eBioscience, San Diego, CA) at 90 °C for the other target proteins. The tissue sections were counter‐stained with Vector Hematoxylin QS (for immunohistochemistry) or 4′,6‐diamidino‐2‐phenylindole (DAPI; for immunofluorescence) supplied by Vector Laboratories (Burlingame, CA). The stained samples were imaged on an Olympus IX81 inverted microscope (Olympus, Waltham, MA) or a Nikon Eclipse E800 upright microscope (Nikon, Melville, NY).

### Establishing Normal miniJoint Chip

The AT, SFT, and OC microtissues were integrated into the miniJoint chamber, where AM, FM, and OM flowed on the tops of the corresponding microtissues, and SM flowed in the bottom stream shared by all three microtissues. Therefore, SM was conditioned by AT, SFT, and OC‐C through mediated crosstalk. The miniJoint culture medium components are provided in Table S1 (Supporting Information). Since the native synovial fluid is chondrosupportive,^[^ ^55^ ^]^ 0.5 ng mL^−1^ TGF*β*3 was added to the SM to maintain the cartilaginous phenotype of OC‐C without causing noticeable AT and SFT chondrogenesis. In addition, given its complex function on cartilage and other tissues, dexamethasone, often used in MSC induction medium, was excluded in the SM.^[^ ^56^ ^]^

The pulse flow pattern described earlier was utilized. After 4 weeks (Day 28‐Day 56, Figure 1f), miniJoint tissue phenotypes were analyzed using RT‐qPCR, and histological and immunohistochemical staining.

### Generating Inflammatory miniJoint Chip

After confirming tissue phenotypes in the healthy miniJoint chips, the proinflammatory cytokine IL‐1*β* (10 ng mL^−1^; PeproTech, Rocky Hill, NJ) was introduced in the FM stream to challenge the SFT for 1 week (day 56–Day 63, Figure 1f), during which TGF*β*3, vitamin C and L‐proline were removed from the SM. It was noted that the IL‐1*β* concentration in the synovial fluid of OA patients was 8 ± 16 pg mL^−1^; we thus tested whether such low concentrations would be sufficient to induce miniJoint degeneration.^[^ ^57^ ^]^ After insulting SFT with 10 pg mL^−1^ IL‐1*β*, the SFT‐conditioned medium could not cause any catabolic changes in the engineered cartilage (Figure S21, Supporting Information). Compared with corresponding tissues challenged by 10 ng mL^−1^ IL‐1*β*, the engineered SFT and cartilage treated with 10 ng mL^‐1^ IL‐1*β* both displayed much lower levels of inflammation (Figure S22, Supporting Information). Therefore, an IL‐1*β* concentration of 10 ng mL^−1^ was used in this study. While other tissues were not directly exposed to IL‐1*β*, they had the potential to be affected by the SFT secretome diffused into the shared SM stream. All microtissues were conditioned in rinsing medium for 18 h and were harvested for phenotype analysis using the methods described above. The non‐IL‐1*β* treated (normal) miniJoint served as the control group.

One concern was cartilage degeneration caused by the diffusion of IL‐1*β* through the SFT microtissue to the SM stream. To examine this potential issue and further confirm the role of SFT inflammation in inducing OC‐C degeneration, normal or acellular SFT (GelMA scaffold only) inserts were used in the miniJoint and subjected to IL‐1*β* treatment. After 1 week of culture in the chip, IL‐1*β* (10 ng mL^−1^) was introduced in the FM of chips with cell‐laden or cell‐free SFT and lasted 7 days. Chips with cell‐laden SFT receiving normal FM (without IL‐1*β* addition) served as the control. The OC‐C microtissues from the three groups were collected for transcriptomic analysis.

The establishment of healthy and inflammatory miniJoint models was repeated two times and similar results were obtained.

### RNA Sequencing (RNA‐Seq) and Bioinformatics Analysis

The transcriptome differences among the tissues from normal and inflamed miniJoint chips were examined by RNA‐Seq. Microtissues were lysed with the QIAzol reagent (Qiagen), and RNA was isolated from the lysate using an RNeasy Plus Universal Kit (Qiagen). Library preparation, sequencing, and bioinformatics analysis were performed according to Medgenome (CA, USA). Total mRNA was processed for next‐generation sequencing performed with the Illumina HiSeq 2500 system (San Diego, CA). Clontech SMARTer UltraTM Low Input RNA Kits (Mountain View, CA) and NexteraXT (San Diego, CA) kits were used for library preparation.

Quality control was first applied on raw RNA sequencing reads by tool FastQC.^[^ ^58^ ^]^ Low‐quality reads and adapter sequences were filtered out by tool Trimmomatic.^[^ ^59^ ^]^ Surviving reads were then aligned to human reference genome hg38 by Hisat2 aligner and gene counts were quantified by HTSeq.^[^ ^60^ ^]^ Differential expression analysis were performed based on gene counts by R package “DESeq2” and DEGs were selected by *p*‐value < = 0.05 and fold change > = 1.5.^[^ ^61^ ^]^ These DEGs were then applied to Ingenuity Pathway Analysis (IPA) to detect enriched pathways. This software includes databases of prebuilt pathways with known genes summarized from previous studies, and checks the overlap between the DEG list and known pathways and performs statistical tests to determine the enrichment. Significant pathways were defined by *p*‐value < = 0.05. Statistically stringently, FDR = 5% cutoff should be applied to control the false discovery rate. In order to encourage more gene candidates, this study went by *p*‐value< = 0.05 and fold‐change> = 1.5 cutoff. The numbers of DEGs defined by *p*‐value cutoff are shown in the corresponding Supporting Information Tables. All the tools were run by default parameter settings.

### Public Human/Animal Study Data Mining

Gene expression profiles for two human OA patient studies and one mouse study were explored. For human study GSE114007,^[^ ^15^ ^]^ gene counts across 18 normal and 20 OA human knee cartilage tissues were downloaded and compared. For human study GSE57218,^[^ ^34^ ^]^ genome‐wide gene expression profiles were collected across 7 healthy donors and 33 OA tissues. For the mouse study GSE143447,^[^ ^35^ ^]^ transcriptome expressions were quantified from 3 mice in the OA group that underwent surgical DMM and 3 mice in the sham‐operated control group. Within each study, similar differential expression analyses were performed as the miniJoint RNA‐Seq data. Gene counts were analyzed by R package “DESeq2” and bead‐chip data were analyzed by R package “limma.”^[^ ^61^, ^62^ ^]^ Functional pathways were identified based on the DEGs, and common significant pathways were compared across the four studies. Network plot for DEGs in a selected pathway was drawn by the software Cytoscape.^[^ ^63^ ^]^

### Luminex Multiplex Assays

Components of the SM, including tissue‐specific secretome, proinflammatory cytokines, and degenerative enzymes, were measured using the Luminex multiplex immunoassay. Eighteen hours before harvesting the microtissues, all medium streams were carefully flushed with ≈30 mL rinsing medium [phenol‐free Dulbecco's modified Eagle's medium supplemented with 1% v/v Insulin‐Transferrin‐Selenium‐Ethanolamine and 1% v/v antibiotic‐antimycotic, all supplied by Gibco, Grand Island, NY] to remove the residual miniJoint culture medium. The tissues were then incubated in the rinsing medium for 18 h. Afterward, the conditioned medium was centrifuged at 14 000 g for 10 min before flash freezing the supernatant in liquid nitrogen. Luminex assays were carried out using the Bio‐Plex 200 system (Bio‐Rad). The Bio‐Plex Manager 6.1 software was used for data collection and analysis. The analyte panels used in this study were either commercial or custom‐designed products. The specific analytes and vendor information of each panel are provided in Table S12 (Supporting Information).

### Drug Testing

After a 3‐day IL‐1*β* treatment of the SFT, naproxen sodium (Sigma, St. Louis, MO) was added at 5 × 10^−6^ m to all medium streams and remained for 4 days. IL‐1*β* treatment continued for another 4 days as well (7 days of treatment in total). The IL‐1*β* challenged miniJoint without naproxen treatment served as the control. The microtissues and SM were collected for the various analysis described above.

The utility of the miniJoint in drug screening was also assessed by introducing four potential DMOADs, including FGF18 (100 ng mL^−1^; Abcam), SOST protein (250 ng mL^−1^; R&D Systems), SM04690 (1 × 10^−8^ m; Seleck Chemicals, Houston, TX), and IL‐1RA (250 ng mL^−1^; Peprotech). To mimic the intraarticular use of these drugs, they were added only to SM and remained for 4 days in the inflamed miniJoint. IL‐1*β* treatment of the SFT continued for another 4 days as well (7 days of treatment in total).

### Assessing Donor Variability

The colony‐forming efficiency and trilineage differentiation capability of hBMSCs from each donor were evaluated and summarized in Figures S23 and S24 (Supporting Information), respectively. To understand donor variability in the engineering of 3D microtissues, 3D AT, SFT, and OC tissues with cells from four representative donors were also prepared. While donor‐to‐donor variation was observed, representative hBMSCs from all four donors possessed the capability to differentiate into the desired lineages when cultured with the protocols described above (Figures S25, S26, and S27, Supporting Information).

### Statistical Analysis

Each experiment was carried out with at least 3 biological replicates. Data is presented as mean ± standard deviation unless otherwise specified. Detailed information of sample size, pre‐processing, and statistical methods were specified in each figure legend. Prism 9 (GraphPad, San Diego, CA) was used for statistical analysis. Significance level was set at 0.05 and indicated by * (*p* < 0.05), ** (*p* < 0.01), *** (*p* < 0.001), and **** (*p* < 0.0001).

## Conflict of Interest

The authors declare no conflict of interest.

## Author Contributions

Z.L., R.S.T., and H.L. conceived and designed the experiments; Z.L., Z.L., H.Y., X.Z., L.Y., M.R.‐L., C.R., M.J.M, I.Y., E.N.L., M.R.F, Q.G., K.B.G., B.O., T.H., and B.M. carried out the experiments and analyzed the data; S.L., P.G.A., J.P.F., S.B.G., B.A.B., R.S.T., and H.L. performed the data analysis; Z.L. and H.L. wrote the first draft of manuscript; all authors edited and approved the manuscript; R.S.T. and H.L. obtained the funding.

## Supporting information

Supporting information

Supplemental Video 1

Supplemental Table 1

## Data Availability

The data that support the findings of this study are available from the corresponding author upon reasonable request.
