# Supplementary material for: Human Mesenchymal Stem Cell‐Derived Miniature Joint System for Disease Modeling and Drug Testing
Source: Adv Sci (Weinh). 2022 Apr 18;9(21):2105909. doi: 10.1002/advs.202105909 (PMC9313499; doi:10.1002/advs.202105909)
Supplement: Supplementary file 1 — Supporting information [file ADVS-9-2105909-s001.pdf]

ADVANCED SCIENCE
Open Access

Supporting Information

for *Adv. Sci.*, DOI: 10.1002/advs.202105909

Human Mesenchymal Stem Cell-Derived Miniature Joint System for Disease Modeling and Drug Testing

Zhong Li, Zixuan Lin, Silvia Liu, Haruyo Yagi, Xiurui Zhang, Lauren Yocum, Monica Romero-Lopez, Claire Rhee, Meagan J. Makarcyzk, Ilhan Yu, Eileen N. Li, Madalyn R. Fritch, Qi Gao, Kek Boon Goh, Benjamen O'Donnell, Tingjun Hao, Peter G. Alexander, Bhushan Mahadik, John P. Fisher, Stuart B. Goodman, Bruce A. Bunnell, Rocky S. Tuan, and Hang Lin**

Supporting Information

Human Mesenchymal Stem Cell-derived Knee Joint-on-a-Chip for Disease Modeling and Drug Testing

Zhong Li, Zixuan Lin, Silvia Liu, Haruyo Yagi, Xiurui Zhang, Lauren Yocum, Monica Romero-Lopez, Claire Rhee, Meagan J. Makarcyzk, Ilhan Yu, Eileen N. Li, Madalyn R. Fritch, Qi Gao, Kek Boon Goh, Benjamen O'Donnell, Tingjun Hao, Peter G. Alexander, Bhushan Mahadik, John P. Fisher, Stuart B. Goodman, Bruce A. Bunnell, Rocky S. Tuan and Hang Lin**

Methods

Isolation and expansion of human bone marrow-derived stem cells (hBMSCs). hBMSCs were isolated from the bone marrow flushed out from the trabecular bone as well as femoral head and neck of patients who had undergone total joint arthroplasty (Institutional Review Board approval by University of Washington and University of Pittsburgh). In this study, hBMSCs isolated from 20 donors (10 female and 10 male) were pooled before use (Figure S1a). hBMSCs were expanded in growth medium [GM; Dulbecco's Modified Eagle Medium (DMEM, high glucose; Gibco, Grand Island, NY) with 10% (v/v) fetal bovine serum (FBS; Gemini Bio-Products, West Sacramento, CA), 1X antibiotic-antimycotic (Gibco, Grand Island, NY)] supplemented with 1 ng/mL basic fibroblast growth factor (bFGF; RayBiotech, Norcross, GA). Once 70%–80% confluence was reached, hBMSCs were trypsinized (trypsin-0.25% ethylenediaminetetraacetic acid; Thermo Fisher, Waltham, MA) and passaged.

Colony-forming unit (CFU) assay. One hundred passage 1 (P1) hBMSCs, from each patient donor or the combined cell pool, were seeded on a 10 cm tissue culture dish (Thermo Fisher, Waltham, MA) and cultured for 14 days. The cells were washed twice with phosphate-buffered saline (PBS; Gibco, Grand Island, NY) and stained with 0.5% Crystal Violet (Sigma, St. Louis, MO) in methanol (Thermo Fisher, Waltham, MA) for 8 min. The purple colonies were counted after removing the excessive dye by repetitive washing with PBS.

Trilineage differentiation assay. hBMSCs at P5, from each patient donor or the combined cell pool, were seeded in six-well plates at a density of 20,000 cells/cm^2. Three types of chemically defined induction medium (Table S10) were used to induce chondrogenic, osteogenic and adipogenic differentiation of the cells. After 21 days of culture, the cells were fixed in 4% paraformaldehyde (Thermo Fisher Scientific, Waltham, MA) and stained for the presence of calcium minerals, sulfated glycosaminoglycans (GAGs), and lipid droplets with Alizarin Red (Rowley Biochemical, Danvers, MA), Alcian Blue (EKI, Joliet, IL), and Oil Red O (Sigma, St. Louis, MO) dyes, respectively. hBMSCs cultured in control medium (Table S10) for 21 days were also included for comparison purposes.

Engineering microtissues using MSCs from four individual patients. After confirming the trilineage differentiation capability of MSCs from all 20 donors, cells from 4 representative donors were used to engineer 3D microtissues. The age and gender combinations for the 4 donors are: 42M, 43F, 73M, and 79F, representing young male, young female, old male, and old female patients. The 3D microtissues were derived from cell-laden GelMA scaffolds, using the protocols described in the Experimental Section. The differentiation and tissue formation abilities of the MSCs from these 4 patients were evaluated by histology or immunostaining.

Flow cytometry. Flow cytometry (BD FACS Aria™ II cell sorter; BD Biosciences) was employed to assess the expression of the following surface epitopes by the MSCs: cluster of differentiation (CD) 31, CD34, CD45, CD73 and CD90[1], using FITC-conjugated mouse anti-human antibodies (BD Pharmingen™, San Jose, CA).

Optimization of the osteogenic medium. hBMSCs were encapsulated within GelMA and then cultured in different media for up to 28 days (Table S1, Figure S2). Expression of osteogenic marker genes was assessed by RT-qPCR.

RNA extraction. Tissue inserts were removed from the chips using forceps. Cylindrical microtissues were then biopsy-punched (Electron Microscopy Sciences, Hatfield, PA) from the tissue inserts. Because of their biphasic nature, osteochondral microtissues were cut into three equal sections under an Olympus dissecting microscope (Olympus, Waltham, MA) and the top and bottom sections were used to extract "bone" and "cartilage" RNA, respectively. The microtissues were crushed in QIAzol lysis buffer (Qiagen, Germantown, MD), and RNA was extracted using an RNeasy Plus Universal Kit (Qiagen, Germantown, MD) following the manufacturer's protocol.

Tissue fixation and embedding. The microtissues collected from the tissue inserts were first rinsed with PBS. The FT and OC tissues were fixed in 10% buffered formalin (Fisher Scientific, Hampton, NH) overnight at 4°C, dehydrated in ethanol with ascending concentrations (30–100%), and embedded in paraffin. The AT samples were fixed in 4% paraformaldehyde (Electron Microscopy Sciences, Hatfield, PA) overnight at 4 °C, dehydrated sequentially in 10%, 20% and 30% (w/v) sucrose (Sigma, St. Louis, MO), and embedded in frozen cryo-gel (Leica Biosystems, Buffalo Grove, IL). The formalin-fixed paraffin-embedded (FFPE) samples were sectioned to 6 μm thick slices, and the frozen AT sections were typically 15 μm thick.

Histology. The OC sections were stained with Safranin O/Fast Green (Sigma, St. Louis, MO) and Alcian Blue to visualize GAGs in the cartilage matrix, with cell nuclei stained by hematoxylin (Sigma, St. Louis, MO) and nuclear fast red (Electron Microscopy Sciences, Hatfield, PA), respectively. To stain mineralized section in the OC unit, the fixed microtissue was rinsed thoroughly with PBS and stained with 40 mM Alizarin Red solution (Sigma-Aldrich). The stained tissue was sliced for imaging on an Olympus dissecting microscope. In addition to Oil Red O, BODIPY® fluorophore (Molecular Probes, Eugene, OR) was also utilized for lipid droplet staining in the AT microtissues. The fixed AT tissues were stained in 20 μg/mL BODIPY® solution at 37C° for 30 min and imaged using an Olympus Fluoview 1000 I confocal microscope (Center Valley, PA).

Chondrocyte pellet culture and treatment with fibroblast-conditioned media. With approval by the University of Pittsburgh Committee for Oversight of Research and Clinical Training Involving Decedents (CORID), human chondrocytes were harvested by digesting minced articular cartilage

with collagenase type II (10 mg/gram cartilage) (Worthington Biochemical Corporation, Lakewood, NJ) at 37 °C for 16 h. The dissociated chondrocytes were filtered through a cell strainer with 70 μm mesh size. The cells were then expanded in GM and passaged at 70–80% confluency. To make pellets, 0.2 million P2 chondrocytes were centrifuged for 10 minutes at 300× g and then cultured in CM for 28 days.

Fibroblasts were derived from hBMSCs following the protocol described in the Experimental Section. Fibroblasts cultured in 6-well plates were treated with 10 ng/mL interleukin (IL)-1β for 24 hours. Afterwards, IL-1β was withdrawn and the cells were rinsed thoroughly with PBS. Subsequently, 1 mL of culture medium containing no IL-1β was added to each well to condition the fibroblasts for 24 hours. The conditioned medium was collected and used to treat the chondrocyte pellets.

Each chondrocyte pellet was treated with 200 μL conditioned medium collected from IL-1β challenged fibroblasts for 3 days, with daily medium changes. For the control group, the pellets were cultured in medium conditioned by fibroblasts that had not been exposed to IL-1β. The fibroblasts and chondrocyte pellets were both collected for RT-qPCR.

Enzyme-linked immunosorbent assay (ELISA). The effluents of healthy and diseased joint models were tested by ELISA to measure the concentration of PGE2, MMP-13, and CTX-II (Table S12) following the manufactures' protocols.

Computational fluid dynamics (CFD). To calculate the shear stress on the surface of the hydrogel scaffold in the miniJoint, CFD analysis was carried out using Fluent 2020 R1 software (Fluent-Ansys Inc, Canonsburg, PA). Top and bottom fluid flows in their respective chambers were separated, assuming there is no leakage. The K-epsilon (k-ε) model parameters with transient flow for 5 seconds were applied for the analysis. The number of elements for the CFD analysis was optimized with a mesh sensitivity study. Five different meshes, with 10,953, 20,898, 77,648, 601,652 and 1,098,326 elements, respectively, were tested. An element number of 1,098,326 was found sufficient as further increases in element number caused no significant changes in the simulated velocity field of flow. Therefore, the mesh with 1,098,326 elements was utilized for the CFD analysis (Figure S9a, b).

To validate the CFD analysis, the experimental velocities in the middle of the bottom chamber were measured and compared with the simulation results. First, a construct with the same geometry of the bottom chamber (without base plate) of the miniJoint was 3D printed and attached to a glass coverslip. A syringe pump (Lagato210P, KD Scientific, Holliston, MA) was used to withdraw liquid from a reservoir of fluorescent particle-containing DI water (Fluoro-Max Green and Red Dry Fluorescent Particles, Thermo Fisher, Waltham, MA) into the construct (Figure S9c), using the same pulse flow pattern employed for miniJoint cultures. Three videos of the fluid flow were recorded using a Nikon Eclipse E800 upright microscope (Nikon, Melville,

NY) during the fast flow stage. The videos were then analyzed by an opensource program, PIVlab, to obtain fluid velocities[2]. A representative velocity field of flow derived from the video is shown in Figure S9d. The maximum velocities reported by PIVlab for the three videos were averaged to obtain the average experimental maximum velocity and compared with the simulation results.

Figure S9e shows the velocity field of flow obtained from the CFD analysis. It was determined that the fields of flow for the CFD analysis and 3D printed construct were characteristically similar. The average maximum fluid velocity in the fast flow stage was measured to be 0.917 mm/s in the 3D printed construct and the corresponding value from CFD analysis was 0.919 mm/s. With a small difference (0.218%) between the experimental and simulation data, the CFD analysis was validated.

Subsequently, the CFD analysis was used to calculate and generate heat maps of flow-induced shear stress (Figure S9f). The top and bottom surfaces of the hydrogel scaffold during fast flow ranged from 2,896–4,959 µPa and 1,158–2,835 µPa, respectively (Figure S9g). In the slow flow stage, the fluid flow-induced shear stress on the top and bottom surfaces of the hydrogel scaffold ranged from 38.7–67.4 µPa and 16.5–33.6 µPa, respectively.

Modeling of the concentration of growth factors in GelMA.

Transforming growth factor beta-3 (TGFβ3). The molecular weight of TGFβ3 is 25 kDa (value provided by supplier). The minimal radius of a sphere that can contain a TGFβ3 molecule is 1.93 nm[3]. Their distribution in the hydrogel is determined by: (i) diffusion caused by a concentration gradient, and (ii) externally applied forces, in this case gravity. The TGFβ3 molecules in the hydrogel move upward due to their high concentration at the bottom (x=0). There is an opposing flux due to gravity, forcing the molecules to move downward. To study these opposing fluxes, we can modify Fick's law by adding a convection flux term $c_i v_i$, namely (the subscript i refers to TGFβ3)

$$J_i = -D_i\left(\frac{\partial c_i}{\partial x}\right) + \frac{c_i f_i}{\xi_i} \tag{1}$$

where c_i, D_i, f_i, ξ_i are the concentration, diffusion coefficient, applied force, and the friction coefficient, respectively. Here, we assume a linear relationship between gravity and its associated velocity, since nanoparticles normally diffuse with a constant velocity (the acceleration occurs in a very

small timeframe of nanoseconds). As such, we can denote v_i as f_i/ξ_i.

At equilibrium, the two fluxes are equal, namely $J_i = 0$, thus we can write the following

$$\ln \frac{c_i(x)}{c_i(x=0)} = \frac{1}{D_i \xi_i} \int f_i dx \tag{2}$$

where $\int f_i dx = -m_i g x$ and m_i is the mass of the TGFβ3. The negative sign implies the applied force, f_i, is downward acting, while a positive x indicates the direction is upward. In addition, eq. (1) is a function of partition ratio, K_i, which will enter the equation implicitly

$$K_i = \frac{c_i(x=0)}{c_i^*(x=0)} \tag{3}$$

where $c_i^*(x=0)$ is concentration at the outside boundary of the hydrogel. The K_i value typically ranges from 0.01 to 10, depending on the interactions between the TGFβ3 and the hydrogel, and a conservative estimation for K_i is 0.01-0.5 for a negative partitioning system, as in this case.
Next, we can use the free volume theory to describe the diffusion coefficient as follows:

$$\frac{D_i}{\overline{\overline{D_i}}} = \exp\left(-A_i \frac{\varphi}{1-\varphi}\right) \tag{4}$$

where A_i and φ are the sieving factor and polymer volume fraction, respectively. A describes the type of interaction between the TGFβ3 molecules and the hydrogel (which is approximated to be 60). In addition, Stokes–Einstein equation is used to describe the bulk diffusivity of the TGFβ3.
The last parameter, the friction coefficient, ξ_i, depends on the size and shape of TGFβ3. Here, we can use Stokes's law to describe ξ_i

$$\xi_i = 6\pi\eta a_i \tag{5}$$

where η is the viscosity of the buffer solution (1 mPa·s for diluted PBS), and a_i is the radius of TGFβ3. Finally, by coupling eqs. (2)-(5), we can obtain the distribution profile of the TGFβ3 in the hydrogel.

Bone morphogenetic protein 7 (BMP7). The molecular weight of BMP7 is 26.4 kDa (value provided by supplier). The minimal radius of a sphere that can contain a BMP7 molecule is 1.97 nm[3]. As described in the previous section, the movement of BMP7 molecules is also driven by (i) diffusion and (ii) gravity. We can write the mass conservation equation as follows (the subscript j refers to BMP7)

$$\frac{\partial c_j}{\partial t} = D_j\left(\frac{\partial^2 c_j}{\partial x^2}\right) + \frac{f_j}{\xi_i}\left(\frac{\partial c_j}{\partial x}\right) \tag{6}$$

where at steady state

$$\frac{dc_j}{dx^2} = -\frac{f_j}{D_j \xi_i}\left(\frac{dc_j}{dx}\right) \tag{7}$$

By applying two boundary conditions on eq. (7), (i) $c_j(x = 0) = 0$ and (ii) $c_j(x = L) = \mathrm{K}_j c_j^*(x = L)$, we can obtain

$$\frac{c_j(x)}{c_j^*(x = L)} = \mathrm{K}_j \frac{1 - \exp\left(-\frac{f_j x}{D_j \xi_j}\right)}{1 - \exp\left(-\frac{f_j L}{D_j \xi_j}\right)} \tag{8}$$

Therefore, the distribution profile of the BMP7 in the hydrogel is dependent on the partition ratio, K_j, and height of the hydrogel, L. We can assume that the diffusion coefficient, applied force, and the friction coefficient of BMP7 coincide with its counterpart TGFβ3.

Diffusion test within a dual-flow bioreactor. To validate the simulation results for the diffusion of BMP7 and TGFβ3, fluorescein isothiocyanate–dextran (FD20S-100MG, Sigma-Aldrich), with a similar molecular weight (20 kDa) to that of BMP7 and TGFβ3, was added at 10 mg/mL to either the top or bottom stream of a dual-flow osteochondral bioreactor. After 1 day, the hydrogels were collected and sliced to image the cross-section under an Olympus dissecting microscope. The fluorescence intensity, correlating directly with diffused dextran, was measured at nine different depths with NIH imageJ and normalized to the values recorded at the top and bottom of the sections.

Modeling of IL-1β concentration in GelMA. Like BMP7, IL-1β was also added in a top medium stream of the chip. Therefore, the concentration

profile of IL-1β was derived from that of BMP7 considering the steric (size) effect and assuming negligible influences by other minuscule inter- and intra-molecular forces of the diffusive species[4]. An in-depth investigation of this idealization requires further studies and is beyond the scope of the current study.

The difference in radial size between the two diffusive species, i.e., IL-1β and BMP7, is ~13 % (1.71 nm for IL-1β and 1.97 nm for BMP7). Considering only the steric interaction, the partition ratio, K_i, is a function of the radial size of the diffusive species[5]:

$$K_i \sim (1-\varphi)^{(1+r_i/a_f)^2}$$

where r_i and a_f are the size of the diffusive species and the hydrated fiber radius of the polymer chains, respectively. Here, we used $a_f = 2$ nm, an acceptable value previously reported[6]. The polymer volume fraction, φ, was taken as 0.1 in the highly swollen hydrogel. Therefore, the partition ratio K_i changes by ~5% when r_i is decreased from 1.97 nm to 1.71 nm.

When normalizing each species with its respective concentration in the medium, the species with a smaller size, IL-1β, thus relaxes at a higher normalized concentration than the larger species, as shown in Figure S13. It is worth mentioning that BMP7 and the IL-1β relax in the hydrogel in a similar manner qualitatively, but not quantitatively, due to the idealization mentioned above.

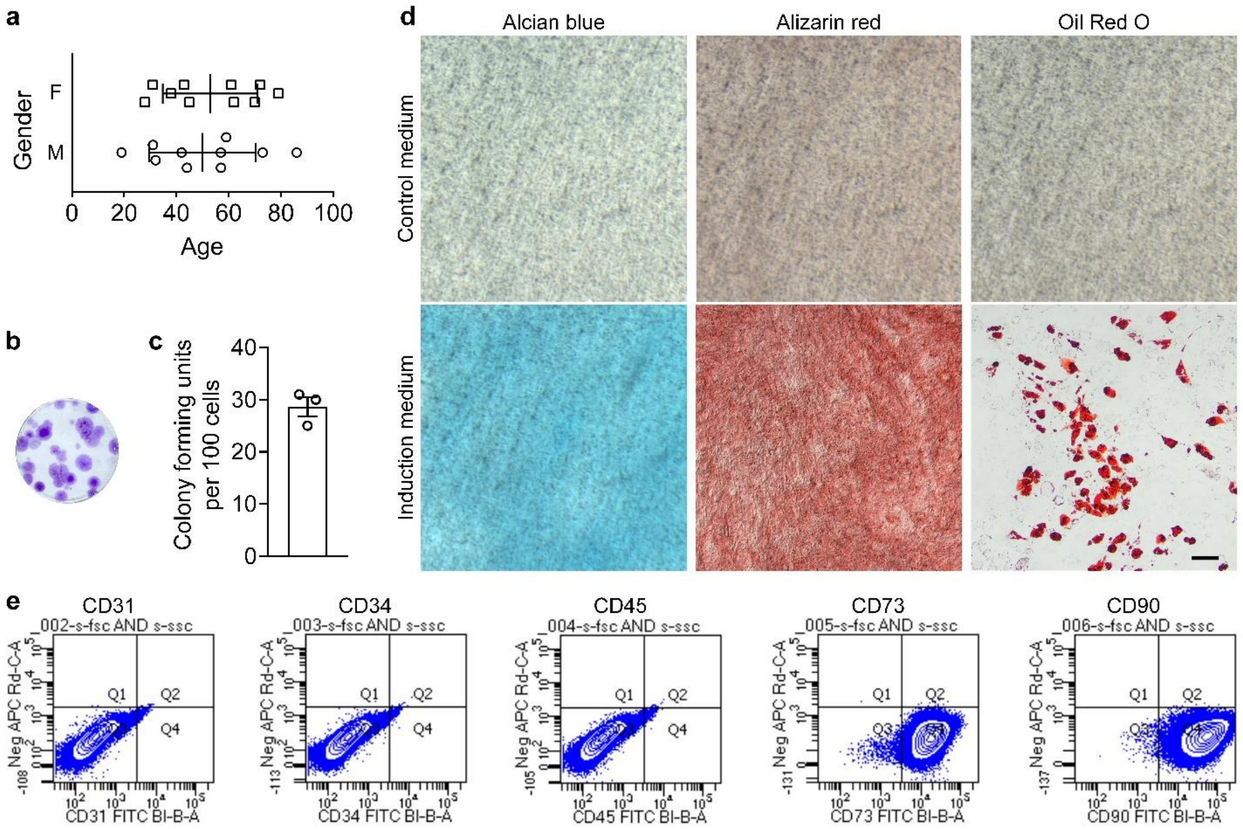

Figure S1. **Characterization of hBMSCs used in this study.** (a) Age and gender information of the 20 deidentified donors. (b, c) CFU assay. A representative image of the colonies formed from 100 initially seeded hBMSCs is given in (b), and the number of colonies per dish is given in (c). CFU assay was conducted in triplicates. (d) Trilineage differentiation assay. Positive staining was observed upon Alcian Blue, Alizarin Red and Oil Red O staining after 21 days of culture in chondro-induction, osteo-induction, and adipo-induction medium, respectively, while no positive staining was observed in the respective control cultures. Scale bar = 100 μm. (e) Flow cytometric analysis revealed that the hBMSCs were negative for CD31 (99.75±0.35%), CD34 (99.7±0.42%) and CD45 (99.7±0.42%) and positive for CD73 (99.45±0.64%) and CD90 (99.6±0%). The percentage values are presented as mean ± standard deviation (N = 2 independent runs carried out 6 months apart).

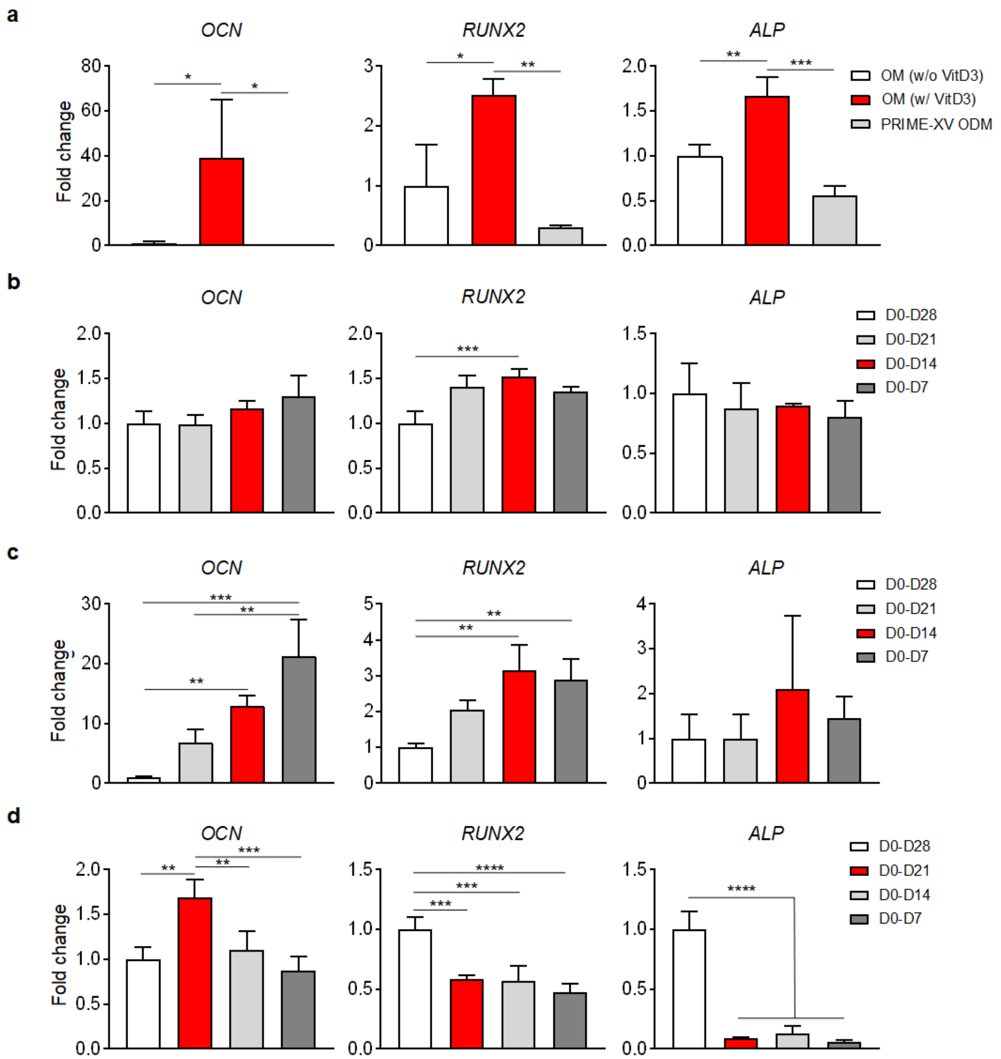

Figure S2. Optimization of induction medium for optimal osteogenesis by hBMSCs in 3D GelMA scaffolds. (a) Expression of selected osteogenic genes by hBMSCs seeded in 15% GelMA hydrogel after 28 days of 3D culture in three types of induction medium, OM without the supplement of 1,25-Dihydroxyvitamin D3 [OM (w/o VitD3); 1,25-Dihydroxyvitamin D3 (VitD3) supplied by Sigma, St. Louis, MO], OM with 10 nM VitD3 [OM (w/ VitD3)], and PRIME-XV ODM: commercially available osteogenic differentiation medium purchased from FUJIFILM Irvine Scientific (Cat# 91132). Statistical analysis by one-way ANOVA (N=3). (b, c) Effect of dexamethasone (Sigma, St. Louis, MO) treatment duration on hBMSC osteogenesis in the absence (b) or presence (c) of BMP7 (Peprotech, Rocky Hill, NJ). Dexamethasone (0.1 μM) was included in OM from day 0-7 (D0-

D7), day 0- 14 (D0-D14), day 0-21 (D0-D21), or day 0-28 (D0-D28). Statistical analysis was conducted using one-way ANOVA (N=4). (d) Expression of selected osteogenic markers by hBMSCs cultured in OM with BMP7 added from D0-D7, D0-D14, D0-D21, or D0-D28. For data presented in all panels, statistical analysis was conducted using one-way ANOVA ($N \geq 3$ biological replicates). *, $p < 0.05$; **, $p < 0.01$; ***, $p < 0.001$; ****, $p < 0.0001$.

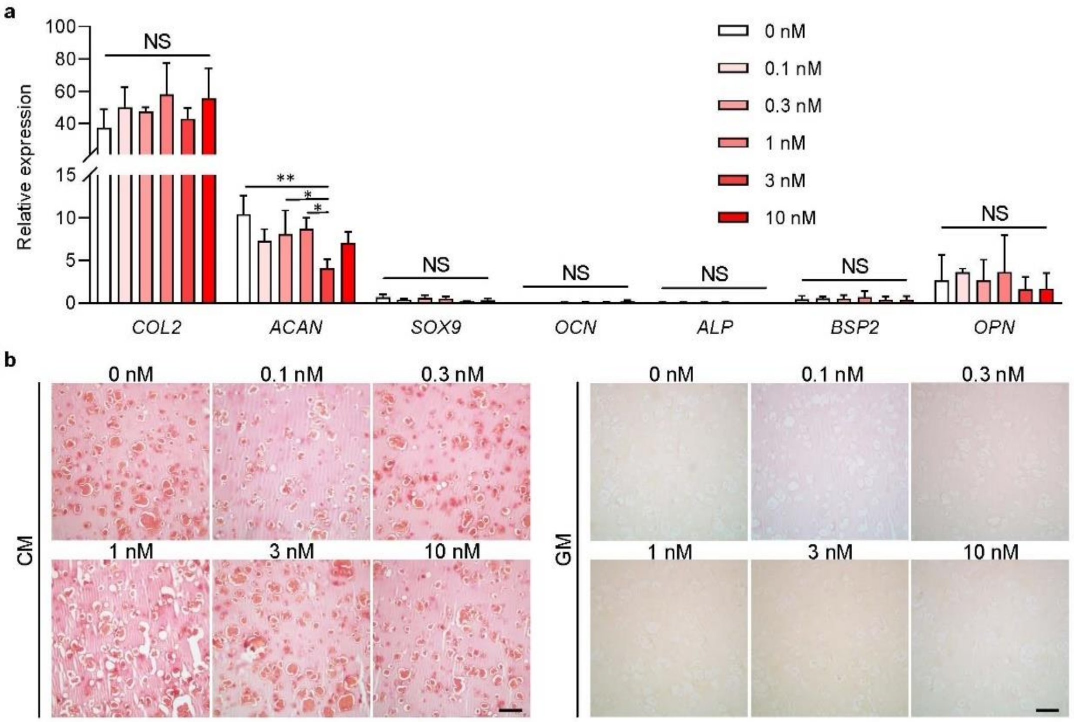

Figure S3. Effect of VitD3 on hBMSC chondrogenesis in 15% GelMA. Since VitD3 is a strong osteoinductive agent, the possible influence of the diffused VitD3 from OC-O on chondrogenesis in OC-C was assessed. (a) RT-qPCR results show that the addition of 0–10 nM of VitD3 in the culture medium over 28 days of chondro-induction had no major effect on the expression of tested chondrogenic or osteogenic marker genes. Experiments were run in quadruplicates. Statistical analysis was conducted using one-way ANOVA. $N \geq 3$ biological replicates. *, $p < 0.05$; **, $p < 0.01$. (b) Safranin O staining of the constructs after 28 days of chondro-induction in medium containing different concentrations of VitD3. Scale bar = 50 μm. Results indicated the supplement of VitD3 in OM did not cause a detrimental effect on the generation of OC-C.

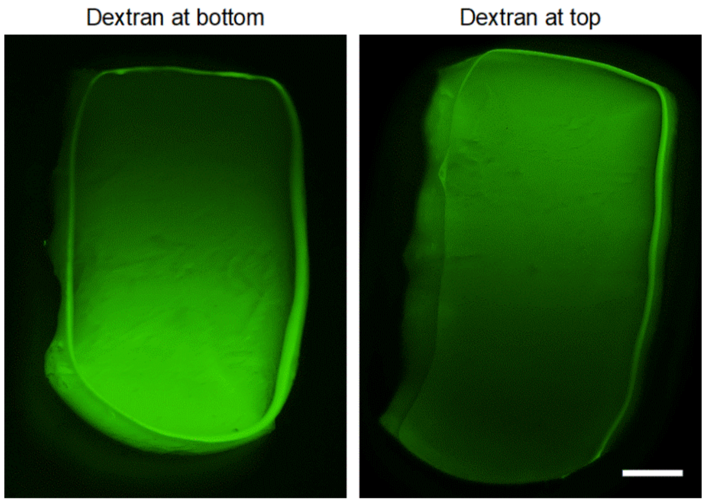

Figure S4 Representative fluorescence images of the gel cross sections after 21 hours of diffusion test in the dual-flow bioreactor. Fluorescein isothiocyanate-labeled dextran was added to either the bottom (left) or top (right) stream. Scale bar = 500 µm.

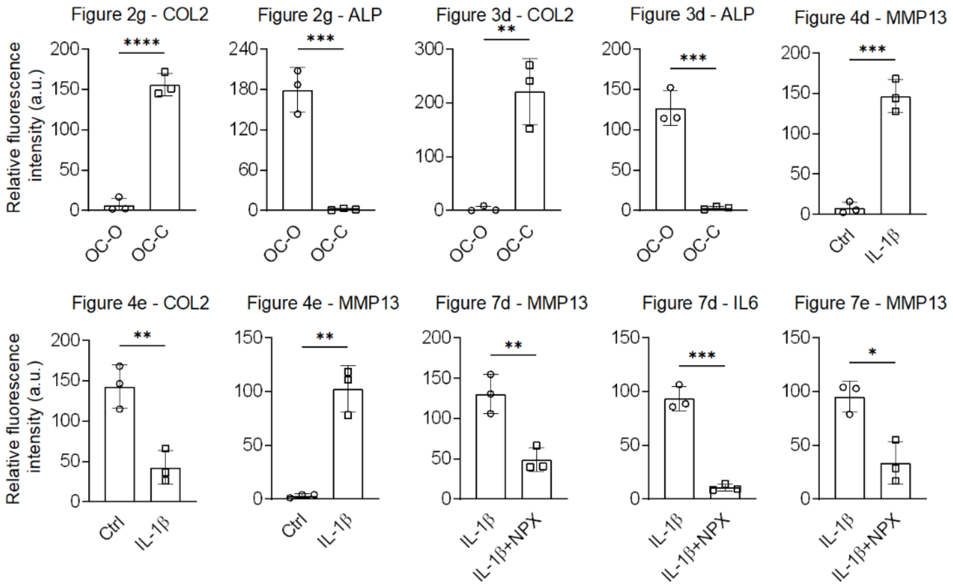

Figure S5 Quantification of the immunofluorescence staining images from different figures. Data were analyzed by the Student's t-test (N =3 fluorescence images taken from 3 biological replicates). *, p<0.05; **, p<0.01; ***, p<0.001; ****, p<0.0001.

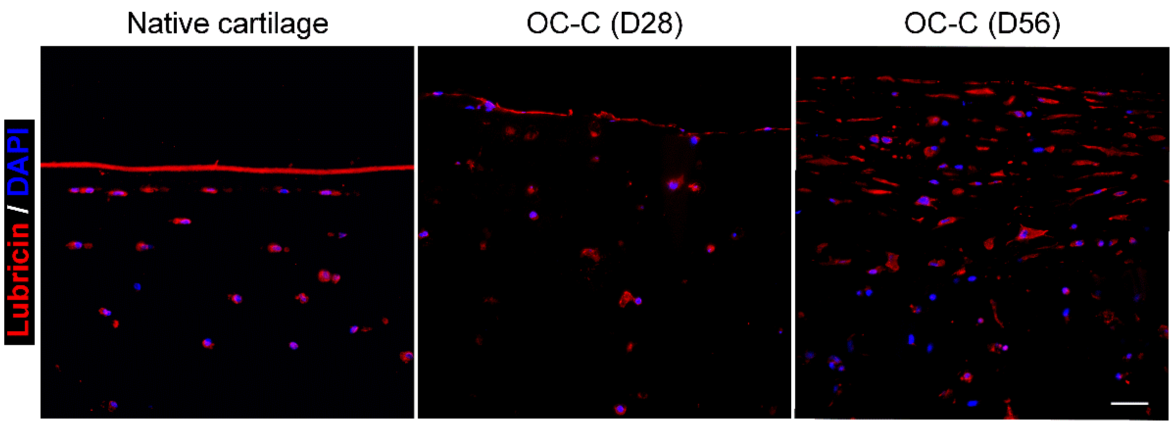

Figure S6. Immunofluorescence staining showing the presence of lubricin in native and engineered cartilage (OC-C) tissue. The native cartilage explant was from a 24-year-old, female donor. OC-C sample on D28 was harvested right after the 4-week differentiation in the dual-flow chip; D56 samples were collected after another 4 weeks of culture in the miniJoint chip. Lubricin staining, red; nuclei, blue (DAPI used as a nuclear counterstain). Scale bar = 50 µm.

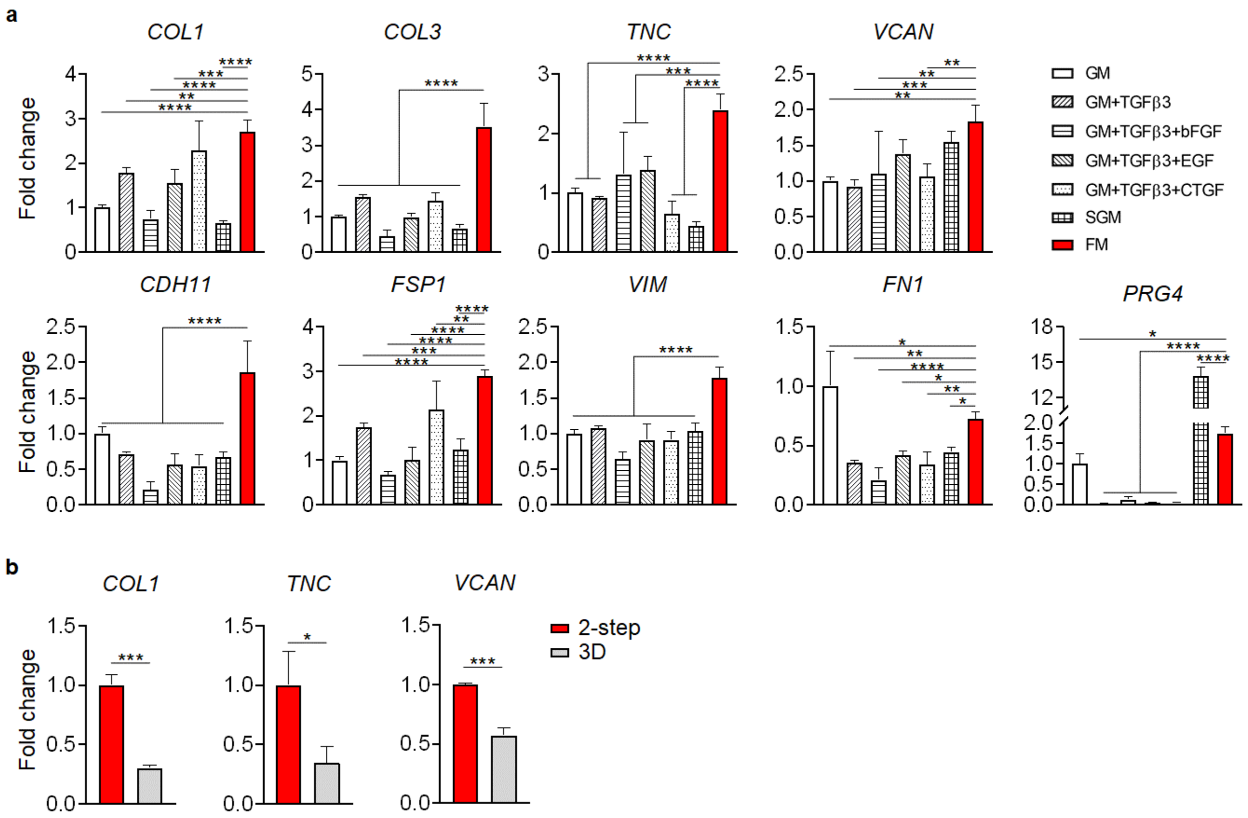

Figure S7. Optimization of protocol for engineering SFT microtissues. (a) hBMSCs were cultured in different media for 21 days. The expression levels of selected marker genes characteristic of fibrous tissue were examined by RT-qPCR. GM: DMEM + 10% (v/v) FBS + 1X antibiotic-antimycotic. TGFβ3 (Peprotech, Rocky Hill, NJ), bFGF, EGF, and CTGF were added at 10 ng/mL, 1 ng/mL, 10 ng/mL, and 100 ng/mL in GM to make different media. Synoviocyte growth medium (SGM) was commercially available (Sigma-Aldrich, cat#415-500). The composition of FM was listed in Supplementary Table 1. Statistical analysis conducted by one-way ANOVA (N = 4 biological replicates). **, $p < 0.01$; ***, $p < 0.001$; ****, $p < 0.0001$. (b) Expression of key genes characteristic of fibrous tissue by MSCs cultured on 2D tissue culture plastic was higher than by cells in 3D 15% GelMA in FM for 3 weeks. Statistical analysis by Student's *t*-test (N = 3 biological replicates). *, $p < 0.05$; **, $p < 0.01$; ***, $p < 0.001$; ****, $p < 0.0001$.

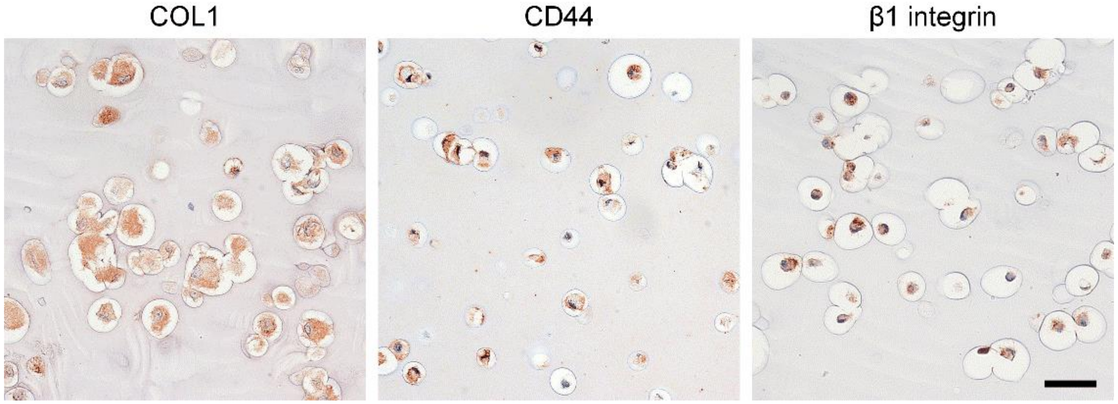

Figure S8. Immunohistochemical (IHC) staining of synovial-like fibrous (SFT) tissue. The IHC images show that the engineered SFT are positive for COL1, CD44 and β1 integrin. Scale bar = 50 μm.

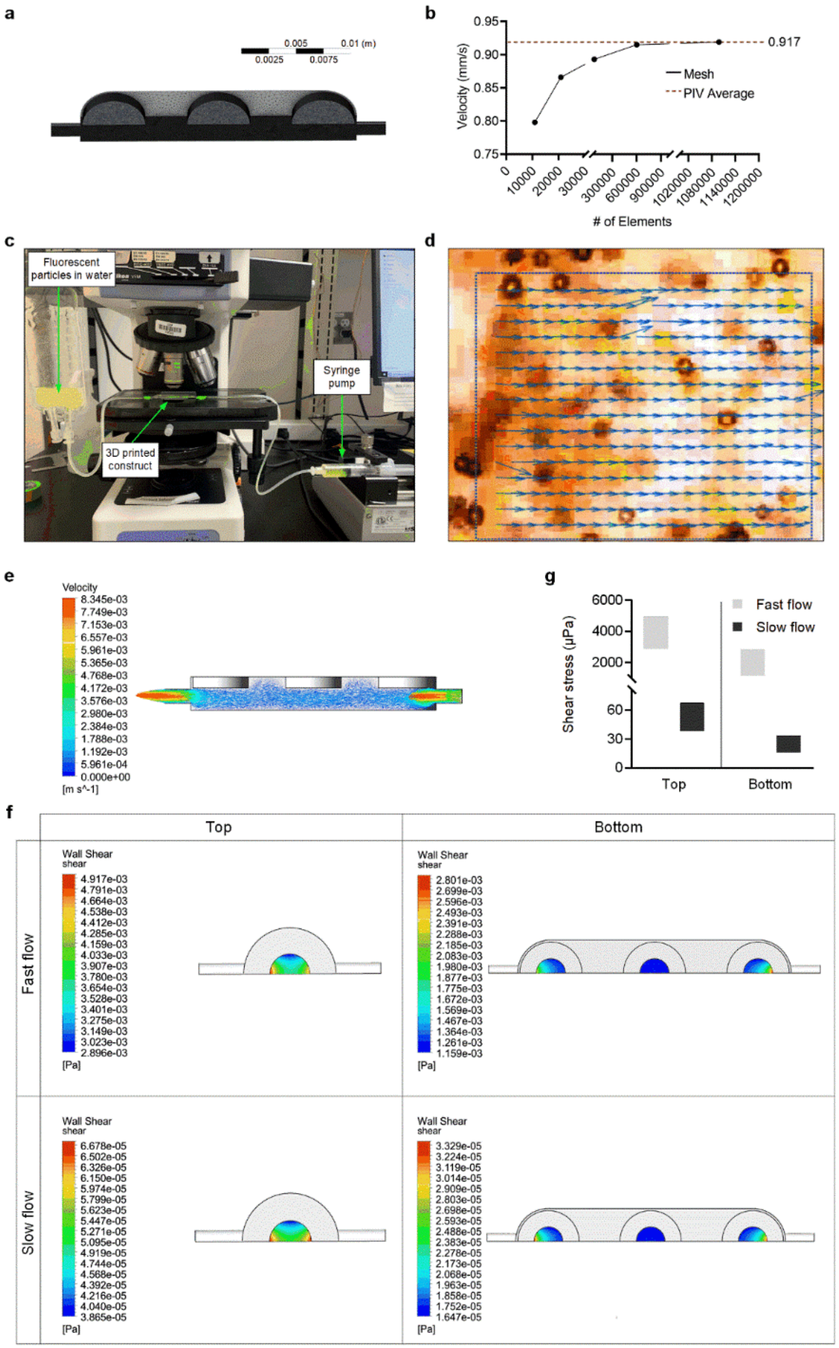
a
0.005
0.01 (m)
0.0025
0.0075
b
Velocity (mm/s)
0.917
Mesh
PIV Average
# of Elements
c
Fluorescent particles in water
Syringe pump
3D printed construct
d
e
Velocity
[m s^-1]
g
Fast flow
Slow flow
Shear stress (μPa)
Top
Bottom
f
Top
Bottom
Fast flow
Slow flow
Wall Shear
[Pa]

Figure S9. CFD analysis. (a) A 1,098,326-element mesh of half the miniJoint bottom chamber for CFD analysis. (b) Maximum fluid velocity calculated by CFD analysis versus number of mesh elements compared to experimentally obtained fluid velocity for bottom chamber. (c) The experimental set up for measuring fluid velocity in the 3D printed construct. (d) Divergence plot of fluid flow within the 3D printed construct. (e) Simulated velocity field of flow for miniJoint bottom chamber during fast flow. (f, g) Heat map (f) and bar chart (g) showing simulated shear stress on top and bottom surfaces of the hydrogel scaffolds.

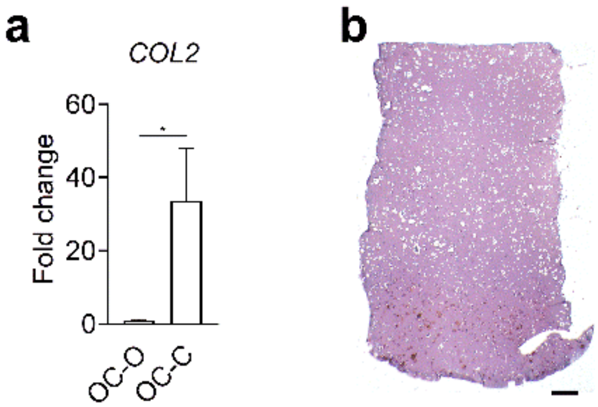

Figure S10. Deprivation of TGFβ3 in the SSF leads to a significant reduction of chondrogenic phenotype in OC-C after 28 days of culture in the miniJoint. (a) Expression of *COL2* by OC-O and OC-C. The expression level of *COL2* in OC-C was only ~34 times higher than OC-O without adding TGFβ3 in the shared medium, as compared to ~2851 times higher when 0.5 ng/mL TGFβ3 was used. Statistical analysis by Student's *t*-test (N = 3 biological replicates). *, $p < 0.05$. (b) Safranin O staining of the OC microtissue, indicating the significant loss of GAGs. Scale bar: 500 µm.

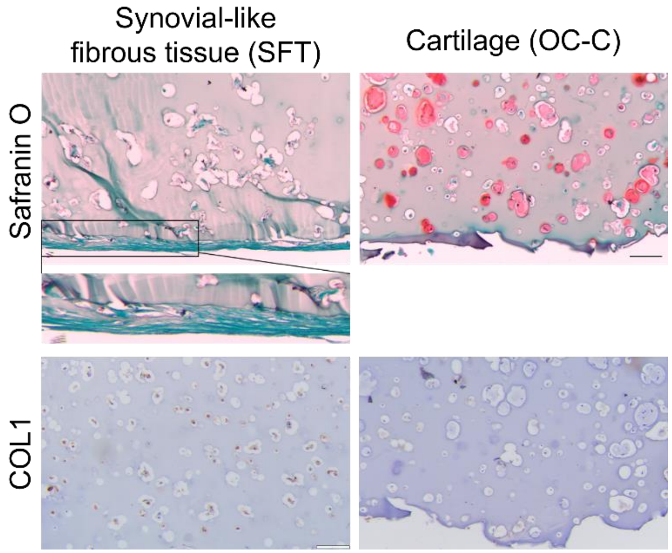

Figure S11 Safranin O staining and immunohistochemical staining of collagen type I (COL1) for SFT and OC-C after 4 weeks of miniJoint culture. Deposition of glycosaminoglycans (GAGs) was not seen in SFT, while robust staining was observed in the cartilage tissue. A fibrous structure was observed on the surface of SFT, but was not see in cartilage. Robust expression of COL1 was observed in SFT, while no positive staining was observed in OC-C. Scale bar = 100 μm.

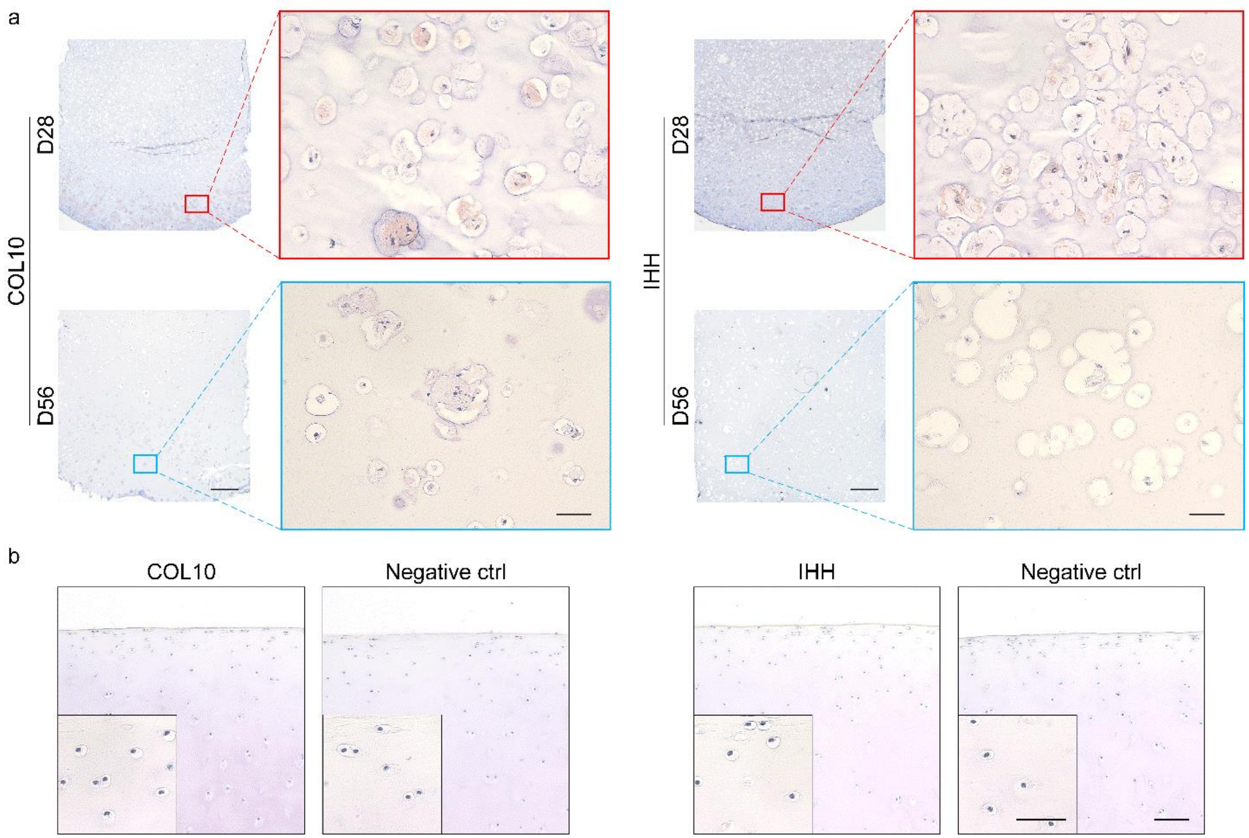

Figure S12. Immunohistochemical staining of collagen type X (COL10) and Indian hedgehog (IHH) in the cartilage (OC-C) tissue from the miniJoint chip and a human donor. (a) The staining intensity of both COL10 and IHH in OC-C decreased with increasing culture time. Scale bar = 500 µm for the low-magnification images & 50 µm for the high-magnification ones. (b) No COL10 or IHH was observed in the superficial/middle zone of articular cartilage from a healthy (without OA), 30-year-old male donor. Scale bar = 100 µm (low-magnification images) or 50 µm (insets).

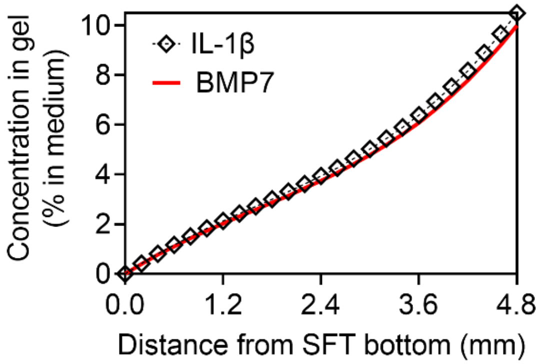

Figure S13. Comparison of simulated IL-1β and BMP7 concentration profiles in the hydrogel scaffold. SFT, synovial-like fibrous tissue.

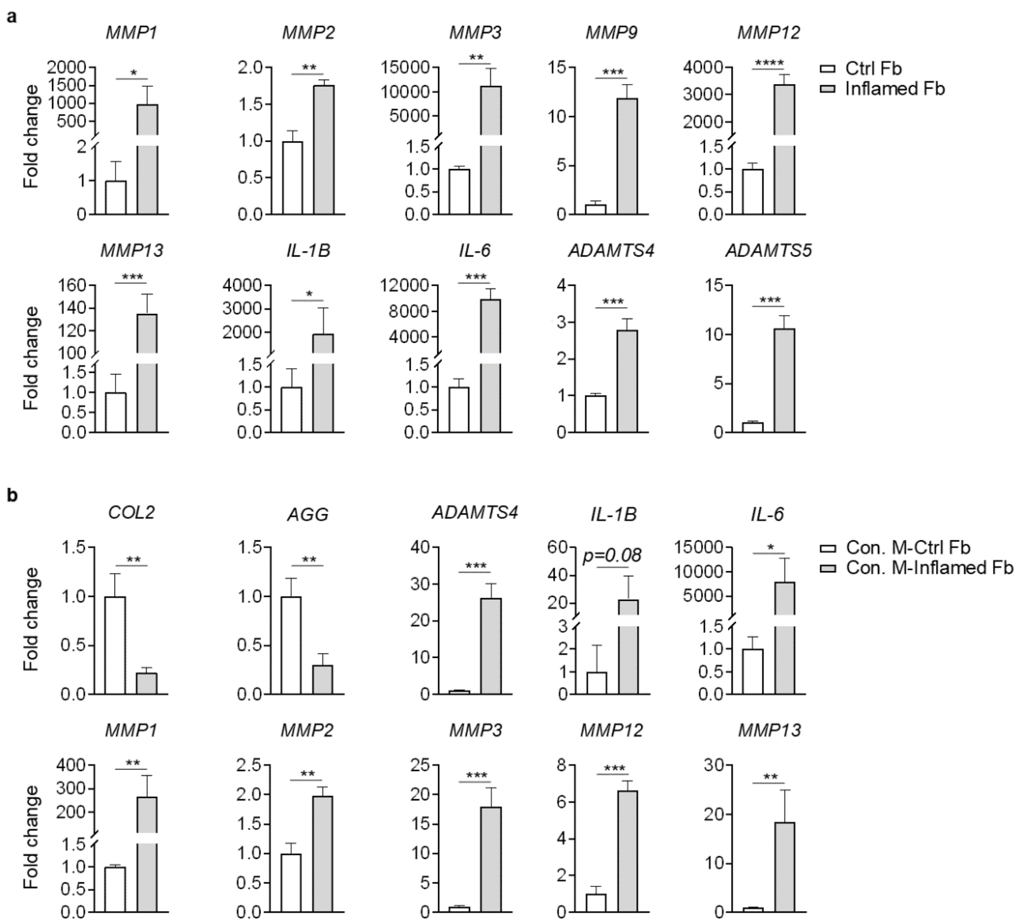

Figure S14. hBMSC-induced fibroblast-like cells (Fb) responded to IL-Iβ challenge and caused the degradation of engineered cartilage. (a) Upon IL-1β treatment (10 ng/mL for 24 hours), hBMSC-derived Fb expressed markedly increased expression levels of inflammatory and degenerative markers. *IL-1B* encodes the cytokine IL-1β. (b) Engineered cartilage derived from chondrocyte pellets were cultured in normal Fb-conditioned medium (Con. M-Ctrl Fb) or that conditioned by IL-1β treated Fb (Con. M-Inflamed Fb) for 3 days. Reduced expression of chondrogenic genes was observed in the Con. M-Inflamed Fb group, which was accompanied by concomitant upregulation of catabolic genes. Data presented in both panels were analyzed using Student's *t*-test (N=3 biological replicates). *, $p < 0.05$; **, $p < 0.01$; ***, $p < 0.001$; ****, $p < 0.0001$.

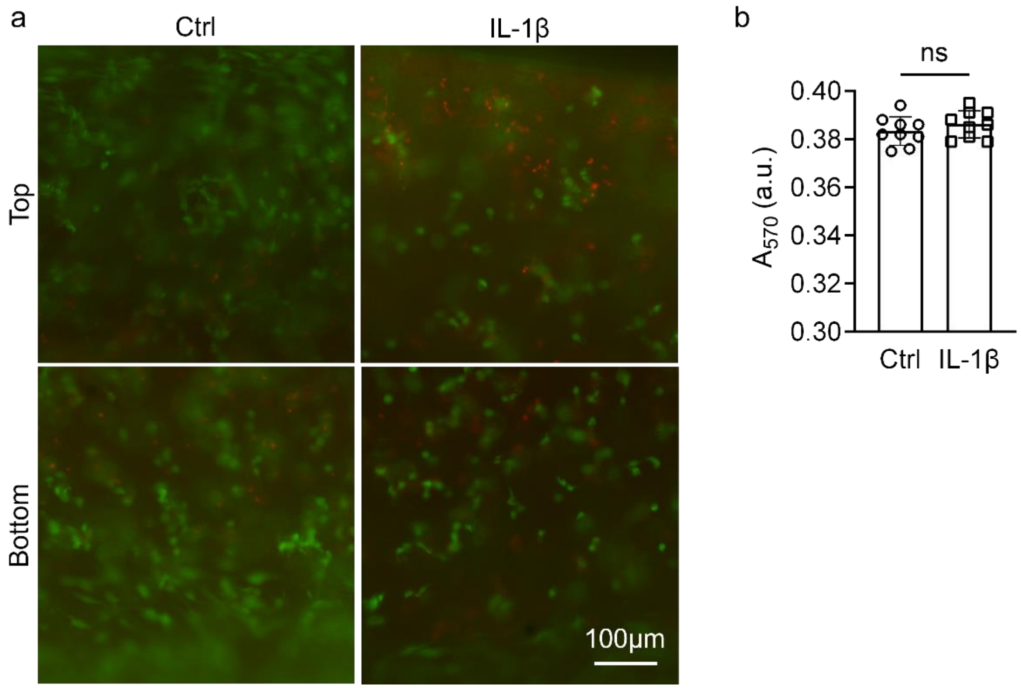

Figure S15. Viability of cells in SFT after 7 days of IL-1β (10 ng/mL) treatment. (a) LIVE/DEAD staining showing that most dead cells located in the superficial layer of the top section of the IL-1β-treated SFT (IL-β), when compared to untreated control (Ctrl). (b) The total metabolic activities of samples treated with and without IL-1β were not statistically different, as revealed by alamarBlue assay. Statistical analysis was conducted by the Student's *t*-test (unpaired). Both assays were performed following the manufacturer's protocols.

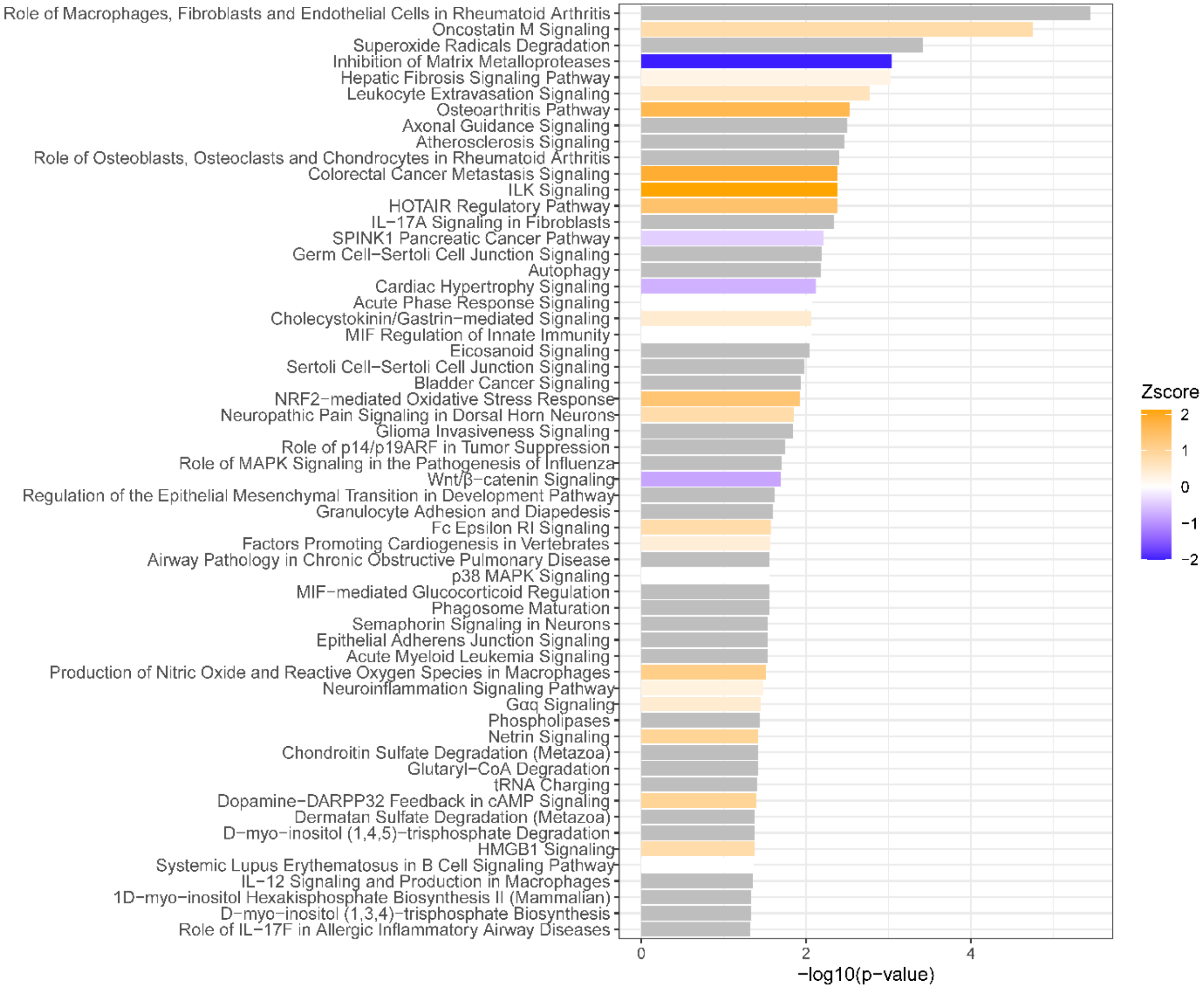

Figure S16. The 58 pathways that were differentially expressed in OC-C from normal miniJoint and that from IL-1β treated, inflamed miniJoint.

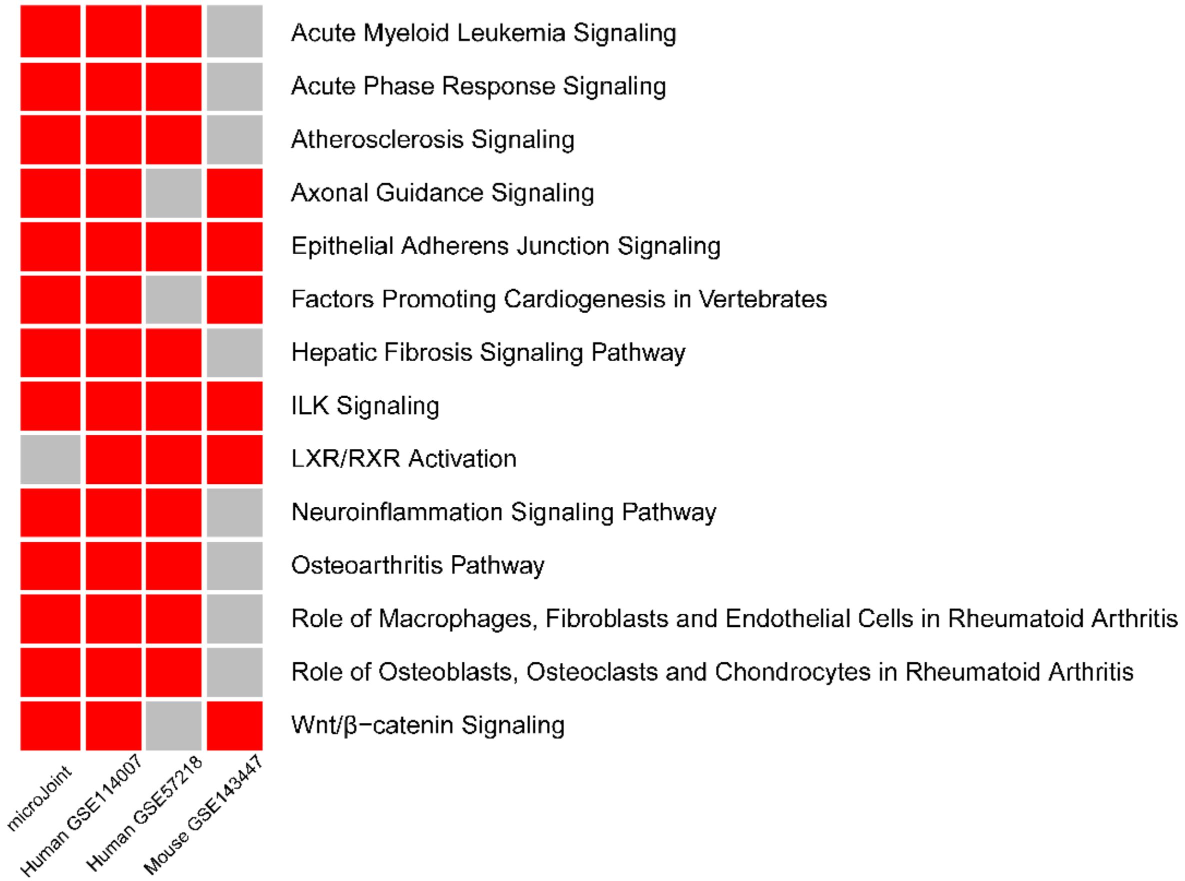

Figure S17. The significant pathways identified in at least 3 out of the 4 systems[7]. Significant pathways are defined as those with -log10(p-value) ≥ 1.3 and indicated by red color.

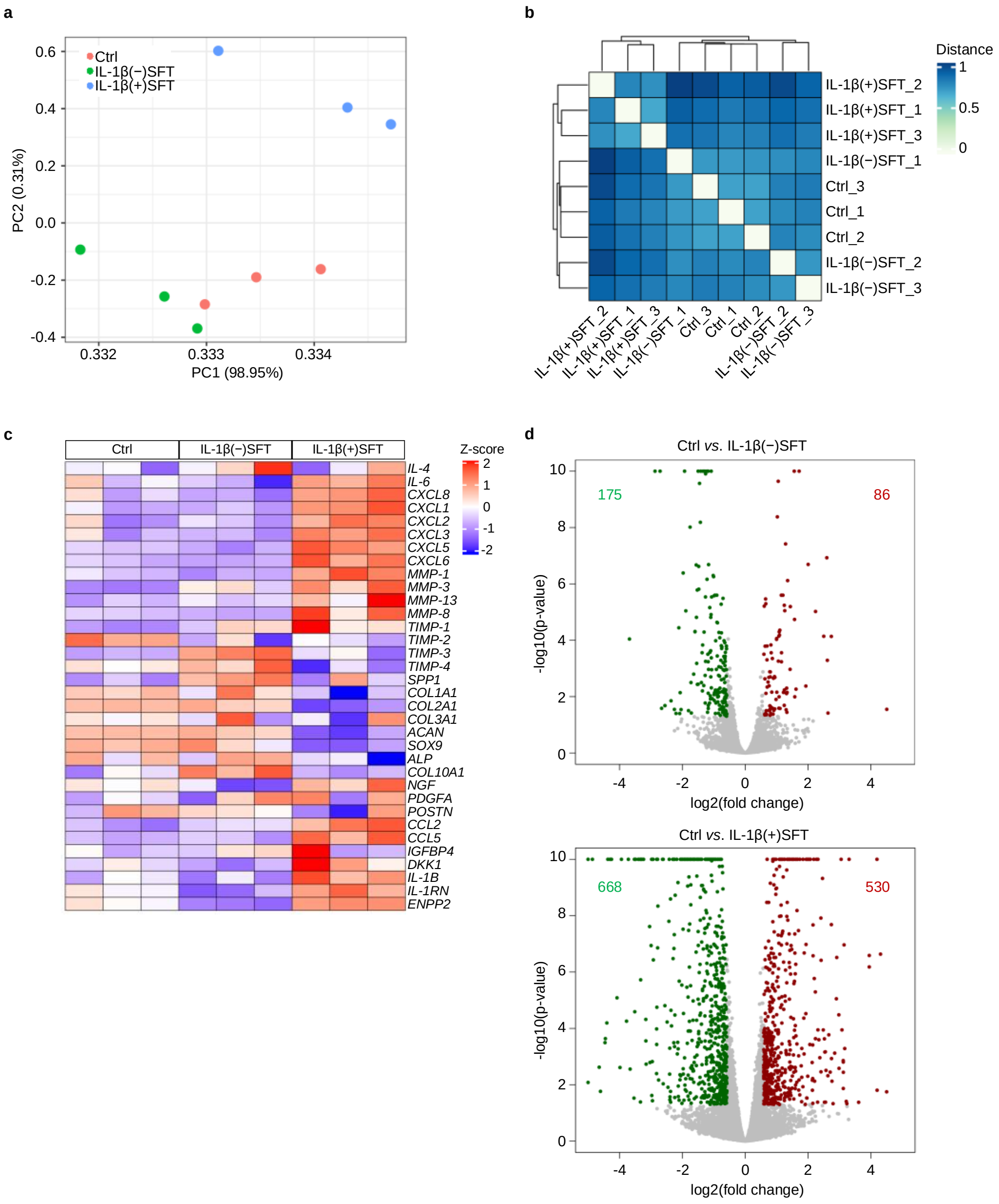

Figure S18. RNAseq data showing that the absence of cells in SFT failed to elicit inflammation-induced cartilage degeneration. (a) Principal component analysis (PCA) of OC-C transcriptome for different chips. Ctrl, healthy miniJoint; IL-1β(−)SFT, IL-1β challenged chip with cell-free SFT; IL-1β(+)SFT, IL-1β challenged chip with cell-laden SFT. N = 3 biological replicates. (b) Distance heatmap showing the relationships between any two samples. Each

entry is colored based on its dissimilarity to another sample, with Distance = 0 indicating smallest dissimilarity and Distance = 1 meaning largest difference. N = 3 biological replicates for each of the 3 sample groups. (c) Heat map generated from RNA-Seq showing the relative expression levels of selected genes in the OC-C of different groups. N = 3 biological replicates. (d) Volcano plot showing the differentially expressed genes (DEGs) in the OC-C of IL-1β challenged chips as compared to the control. Red and green dots denote upregulated and downregulated genes, respectively, with the numbers of genes illustrated in corresponding colors. Gray dots represent nonsignificant genes.

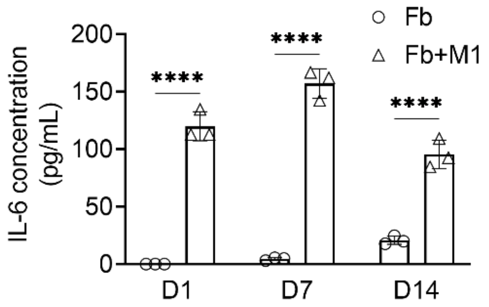

Figure S19. Secretion of interleukin 6 by three-dimensional, macrophage-containing fibrous tissues cultured in a dual-flow chip. Human monocytes were cultured in macrophage-induction medium [Roswell Park Memorial Institute 1640 Medium (RPMI 1640; Life Technologies) supplemented with 10% FBS, 1% antibiotic-antimycotic, and 100 ng/mL macrophage colony-stimulating factor (M-CSF; R&D Systems, Minneapolis, MN)] for 5 days to obtain monocyte-derived macrophages. M0 macrophages were then polarized to an M1 phenotype by adding 10 ng/mL lipopolysaccharide (Sigma, St. Louis, MO) and 20 ng/mL interferon-γ (VWR, Radnor, PA) in the macrophage-induction medium for 2 days. M1 macrophages (M1) were then co-encapsulated with MSC-derived fibroblasts (Fb) in 15% GelMA (20 M/mL, with a Fb to M1 macrophage ratio of 1:2) that was crosslinked in 3D printed inserts. The inserts were then integrated in dual-flow bioreactors (Fig. 2c), through which a mix of macrophage-induction medium and FM (Supplementary Table 1), with a 1:1 volume ratio, was perfused. The effluent from the bioreactors was collected on day 1, 7, and 14 (D1, D7 and D14) to measure the levels of IL-6 using enzyme-linked immunosorbent assay. Gel inserts with only fibroblasts encapsulated (no M1 macrophages) were used as the control. The data were analyzed by the Student's *t*-test. *, $p < 0.05$; **, $p < 0.01$. N = 3 biological replicates.

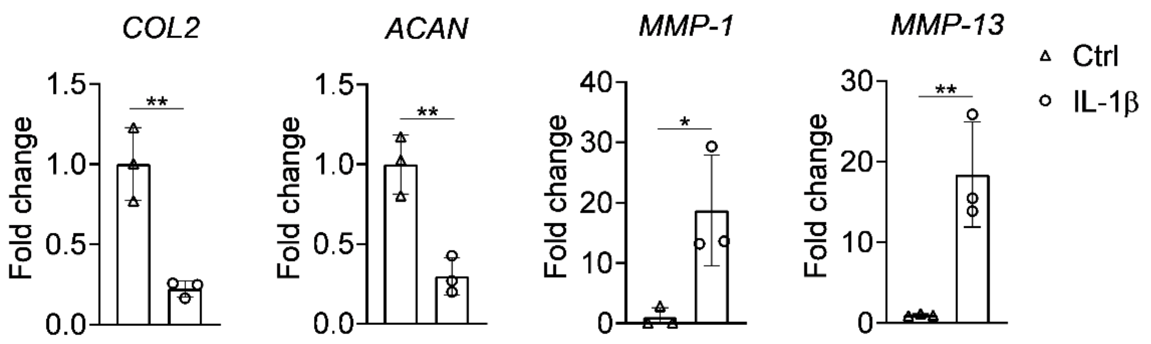

Figure S20. Expression of chondrogenic and degenerative markers in OC-C tissue samples harvested on D63, after 1 week of disease modeling with IL-1β addition to the fibrous tissue-specific medium stream in the miniJoint. The values for the inflamed (IL-1β) group were normalized to the non-IL-1β-treated control. The data were analyzed by the Student's *t*-test. *, $p < 0.05$; **, $p < 0.01$. N = 3 biological replicates.

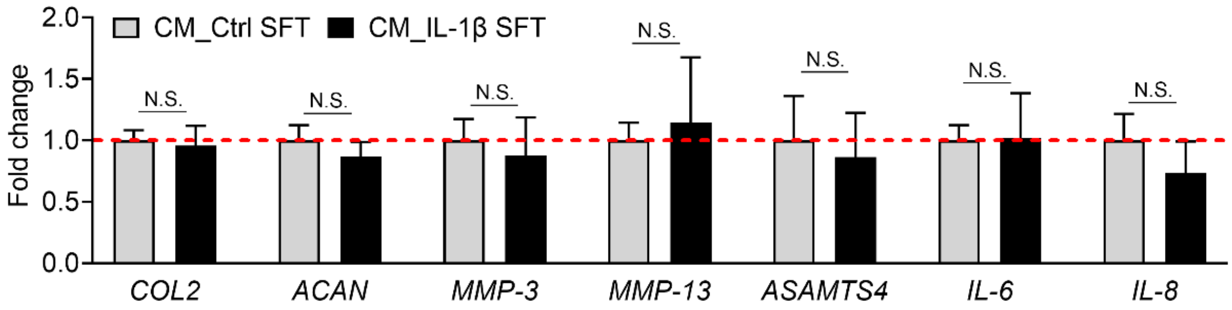

Figure S21. Conditioned media of synovial-like fibrous tissue (SFT) challenged by 10 pg/mL IL-1β did not cause degeneration of engineered cartilage. The SFT was insulted by 10 pg/mL IL-1β for 24 hours. Three-dimensional cartilage engineered from hBMSC-laden GelMA scaffolds was cultured in normal SFT-conditioned medium (CM_Ctrl SFT) or medium conditioned by IL-1β treated SFT (CM_IL-1β SFT) for 3 days. The data were analyzed by the Student's *t*-test. N.S., not significant. N = 3 biological replicates.

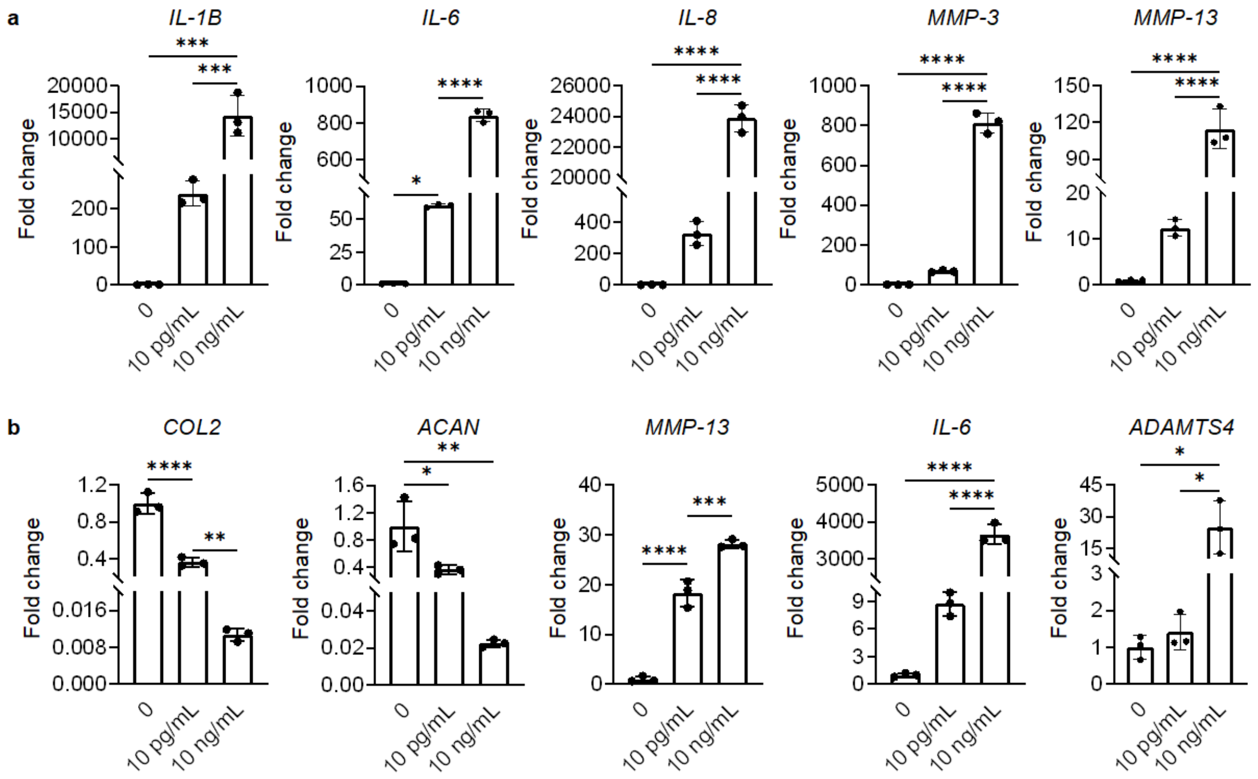

Figure S22. Treatment with IL-1β at 10 pg/mL concentration resulted in significantly lower catabolic changes in the engineered synovial-like fibrous tissue (SFT) and cartilage tissue. (a) Gene expression in engineered SFT challenged with 10 pg/mL or 10 ng/mL of IL-1β for 7 days. N = 3 biological replicates. (b) Gene expression in cartilage tissue engineered from hBMSC-laden GelMA scaffolds over 28 days and challenged with 10 pg/mL or 10 ng/mL of IL-1β for 7 days. The data in both panels were analyzed by one-way ANOVA. N = 3 biological replicates. *, $p < 0.05$; **, $p < 0.01$; ***, $p < 0.001$; ****, $p < 0.0001$.

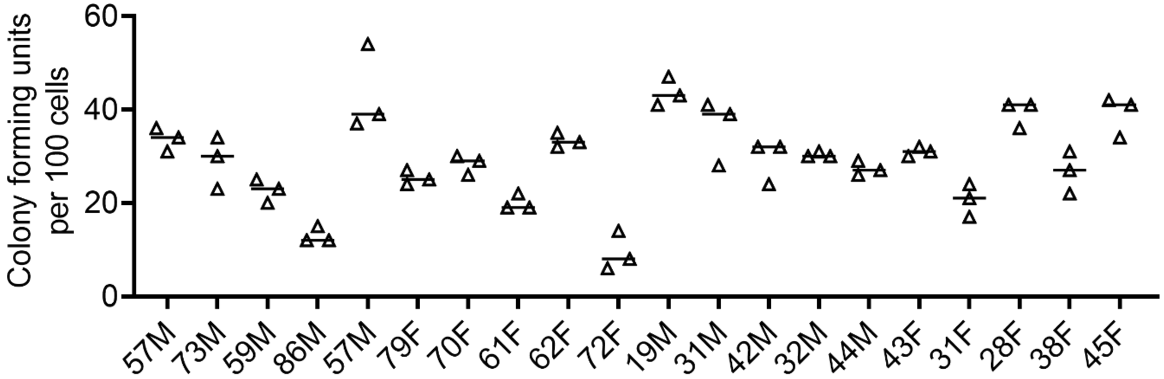

Figure S23. The numbers of colony-forming units from 100 initially seeded hBMSCs from 20 different donors. The deidentified donors are indicated by their age and gender (F, female; M, male). N = 3 biological replicates. The line indicates the median.

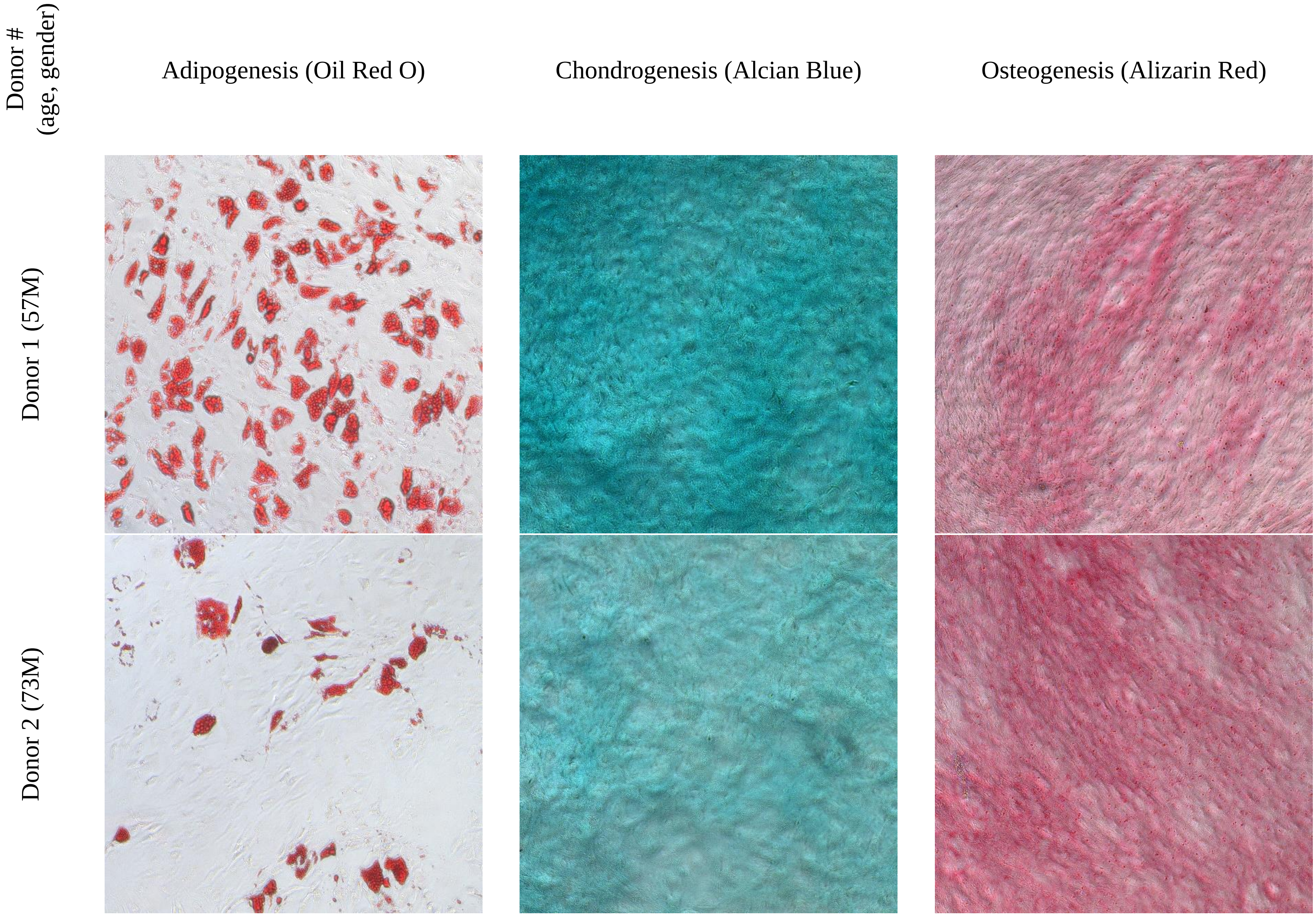

Continued on next page

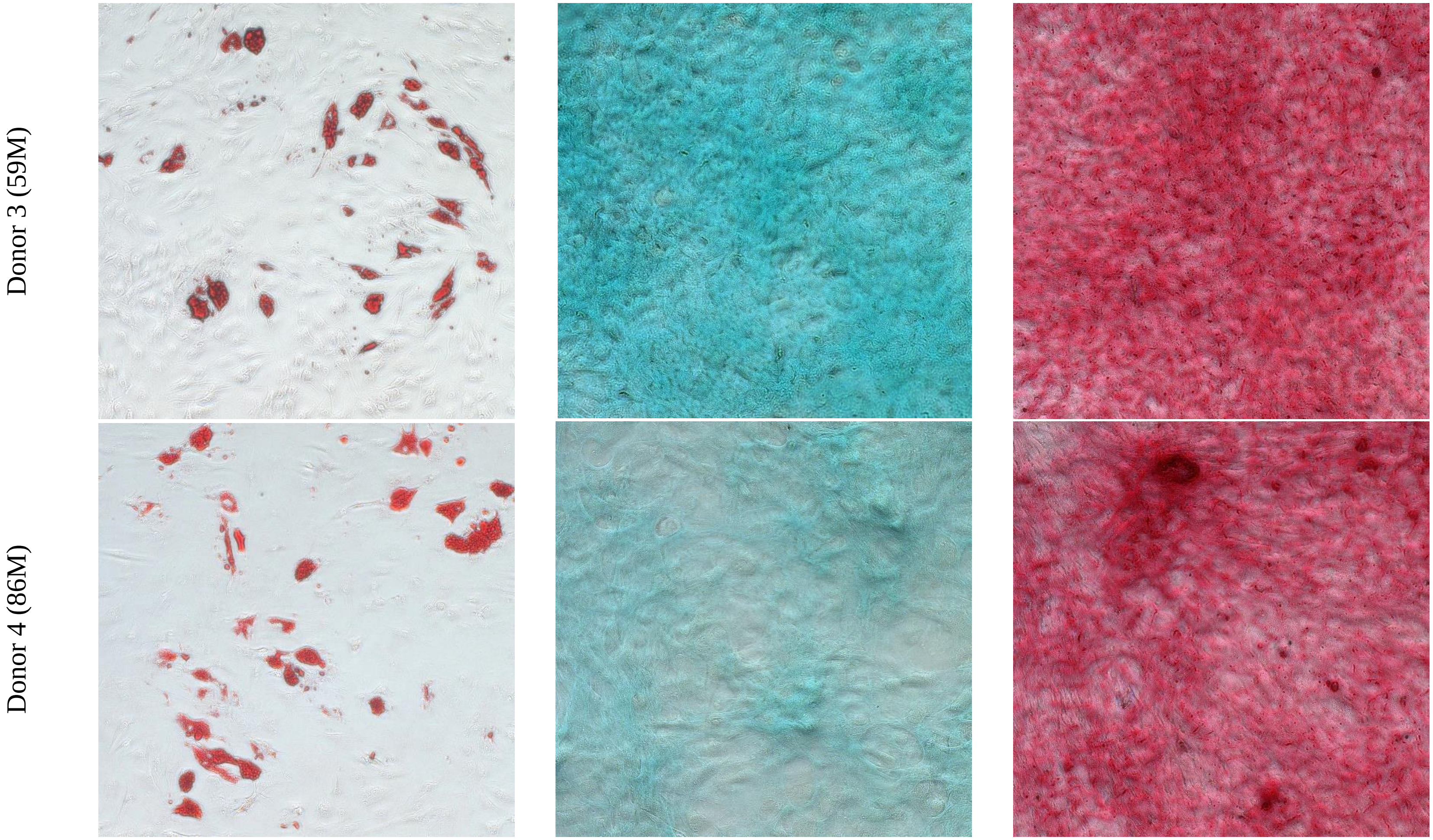

Continued on next page

Donor 5 (57M)

Donor 6 (79F)

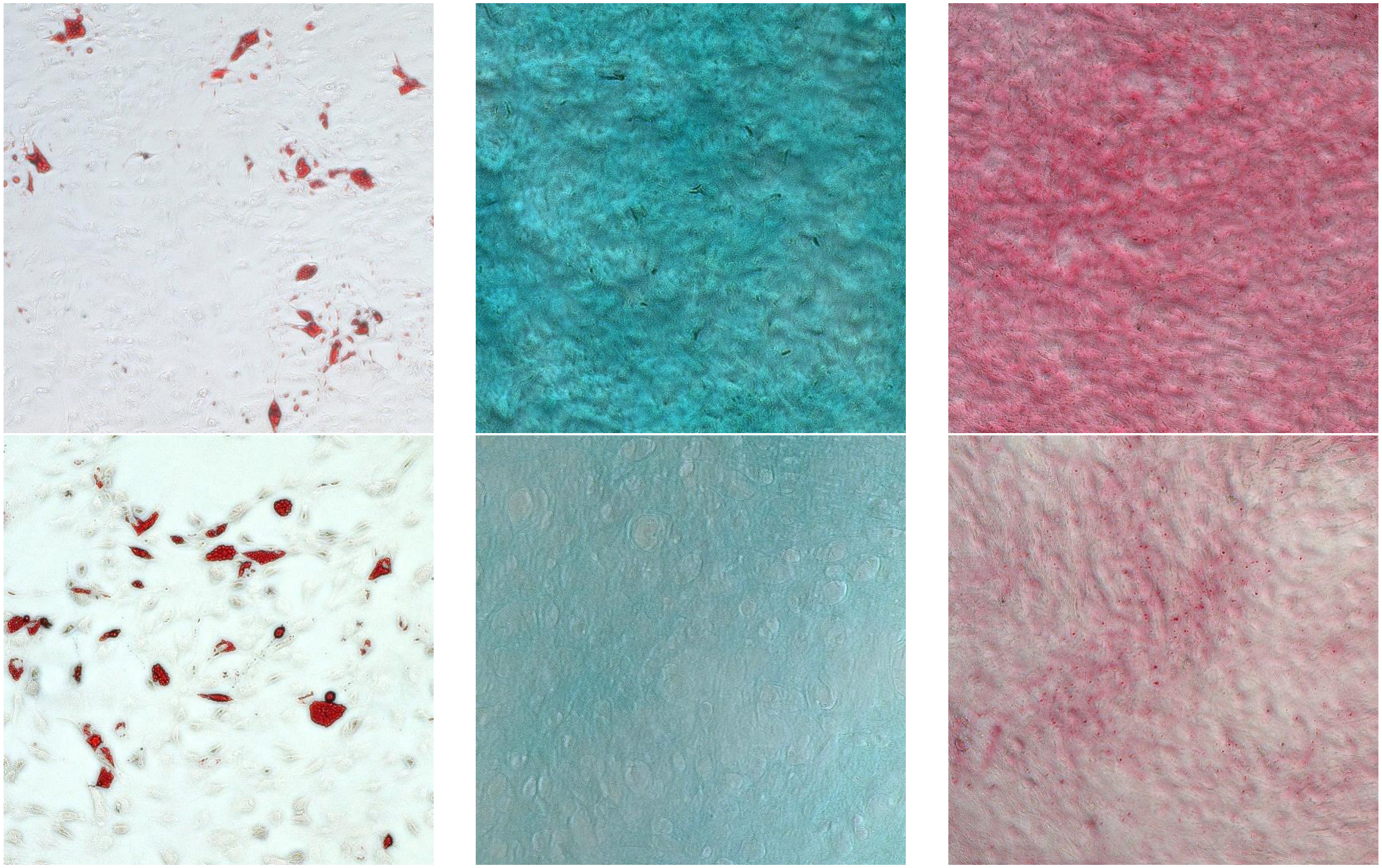

Continued on next page

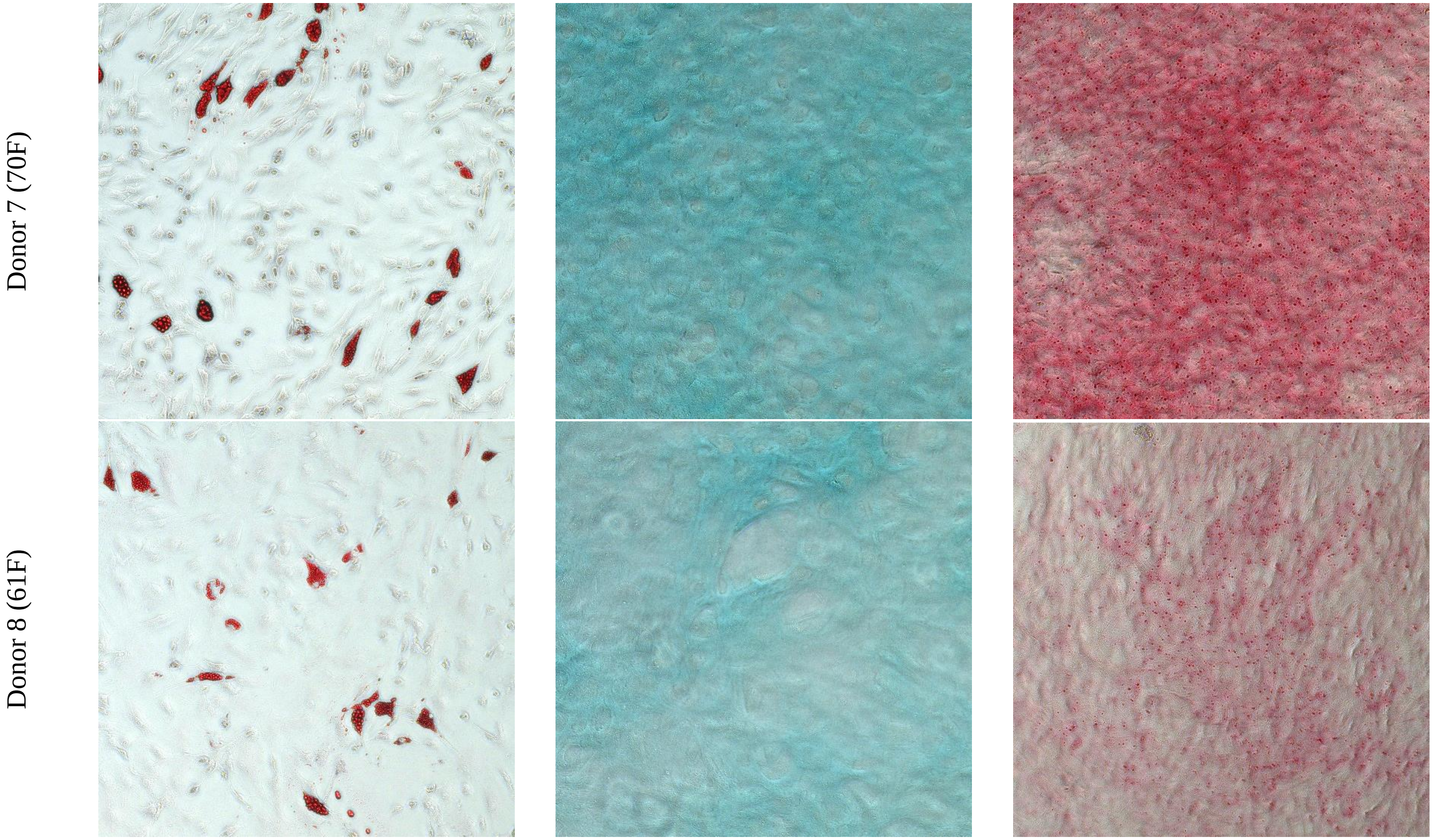

Continued on next page

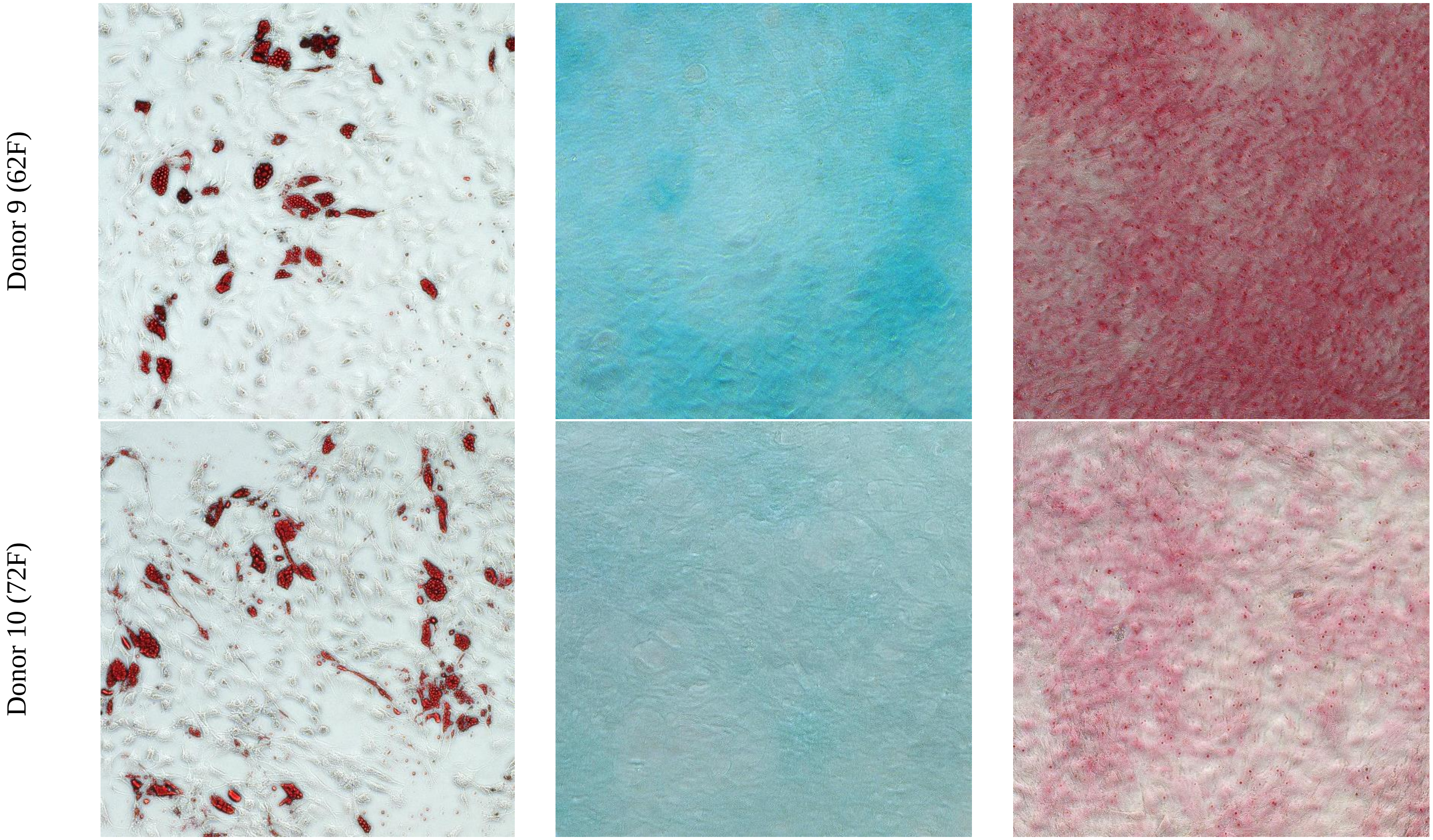

Continued on next page

Donor 11 (19M)

Donor 12 (31M)

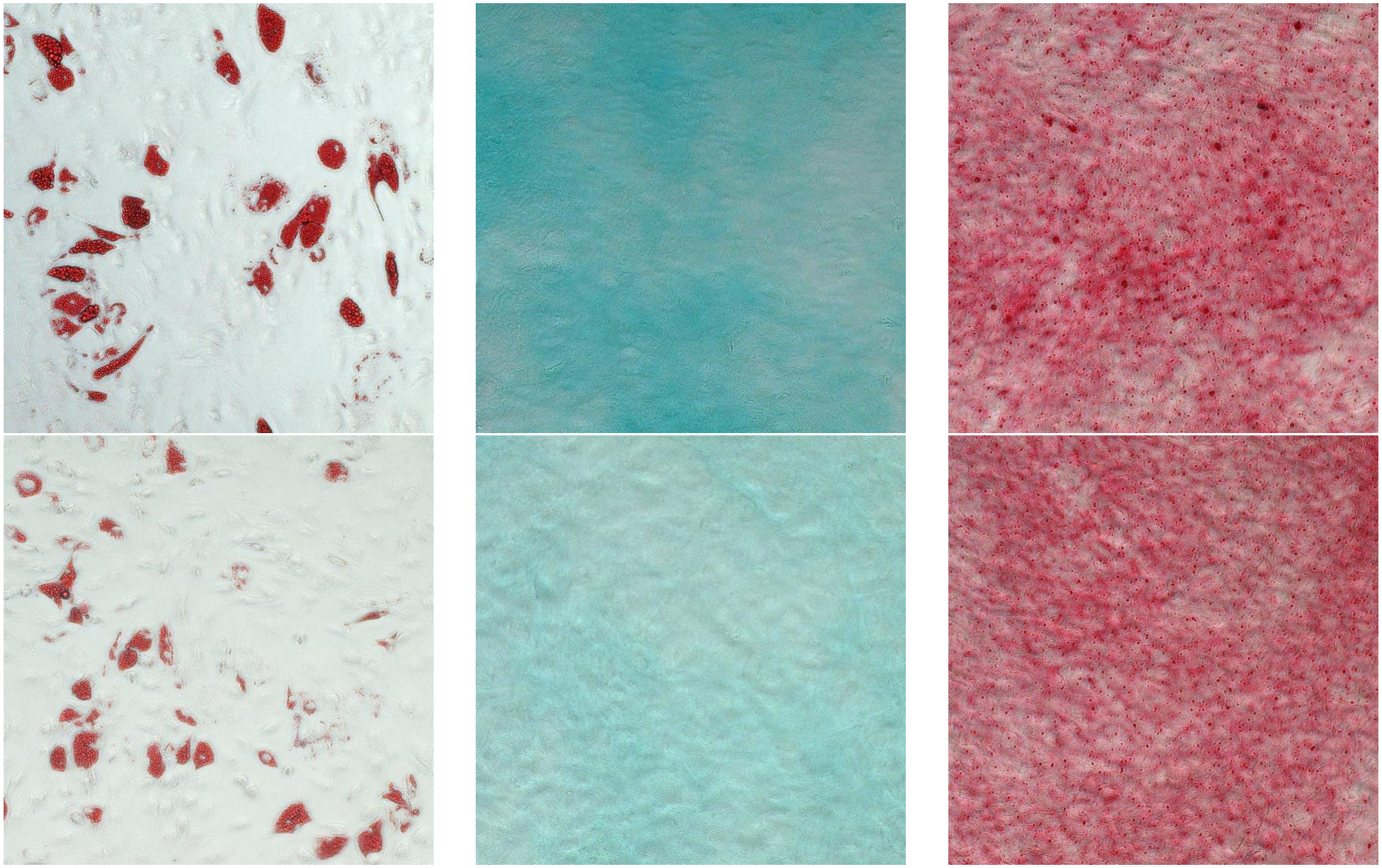

Continued on next page

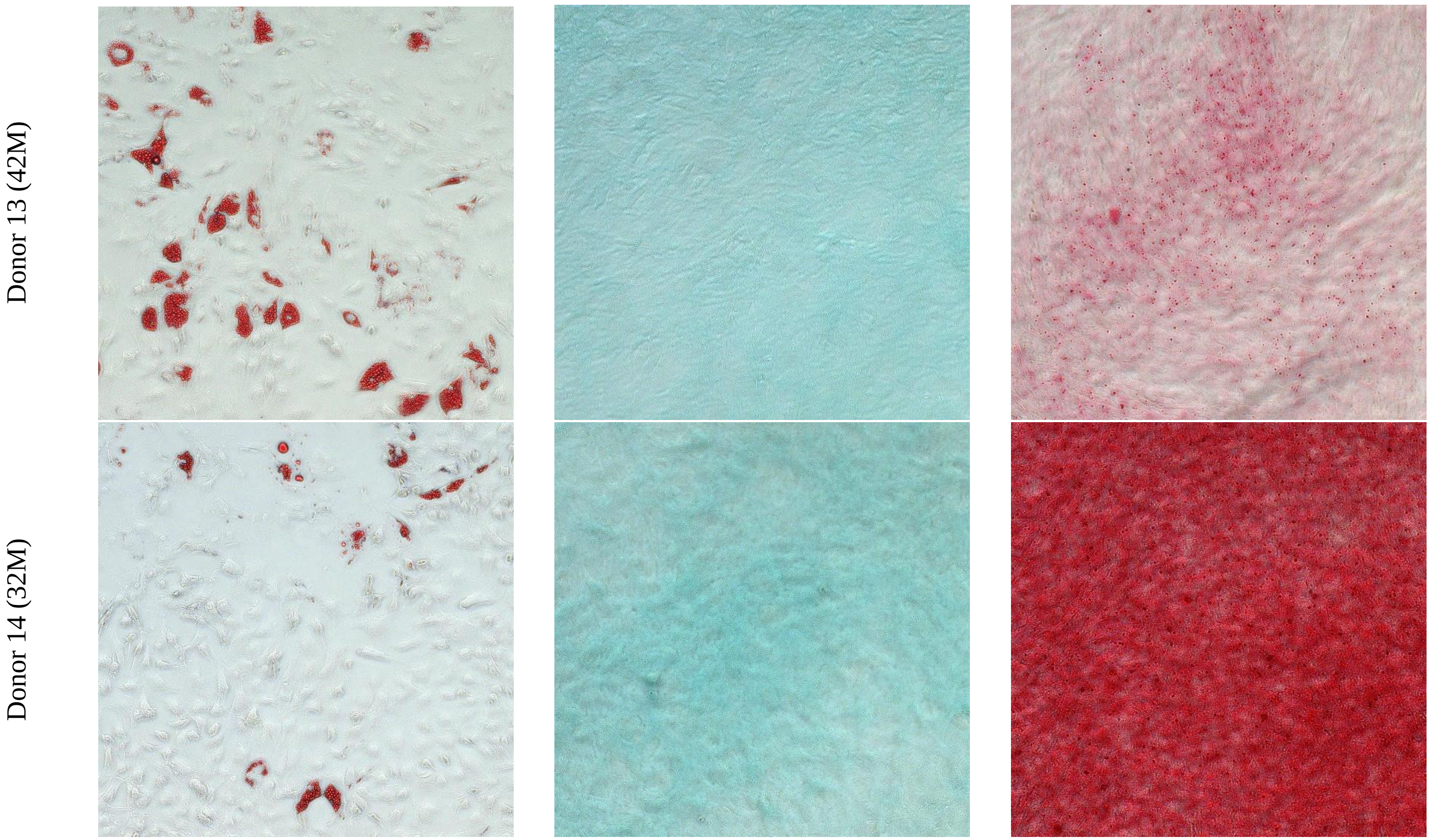

Continued on next page

Donor 15 (44M)

Donor 16 (43F)

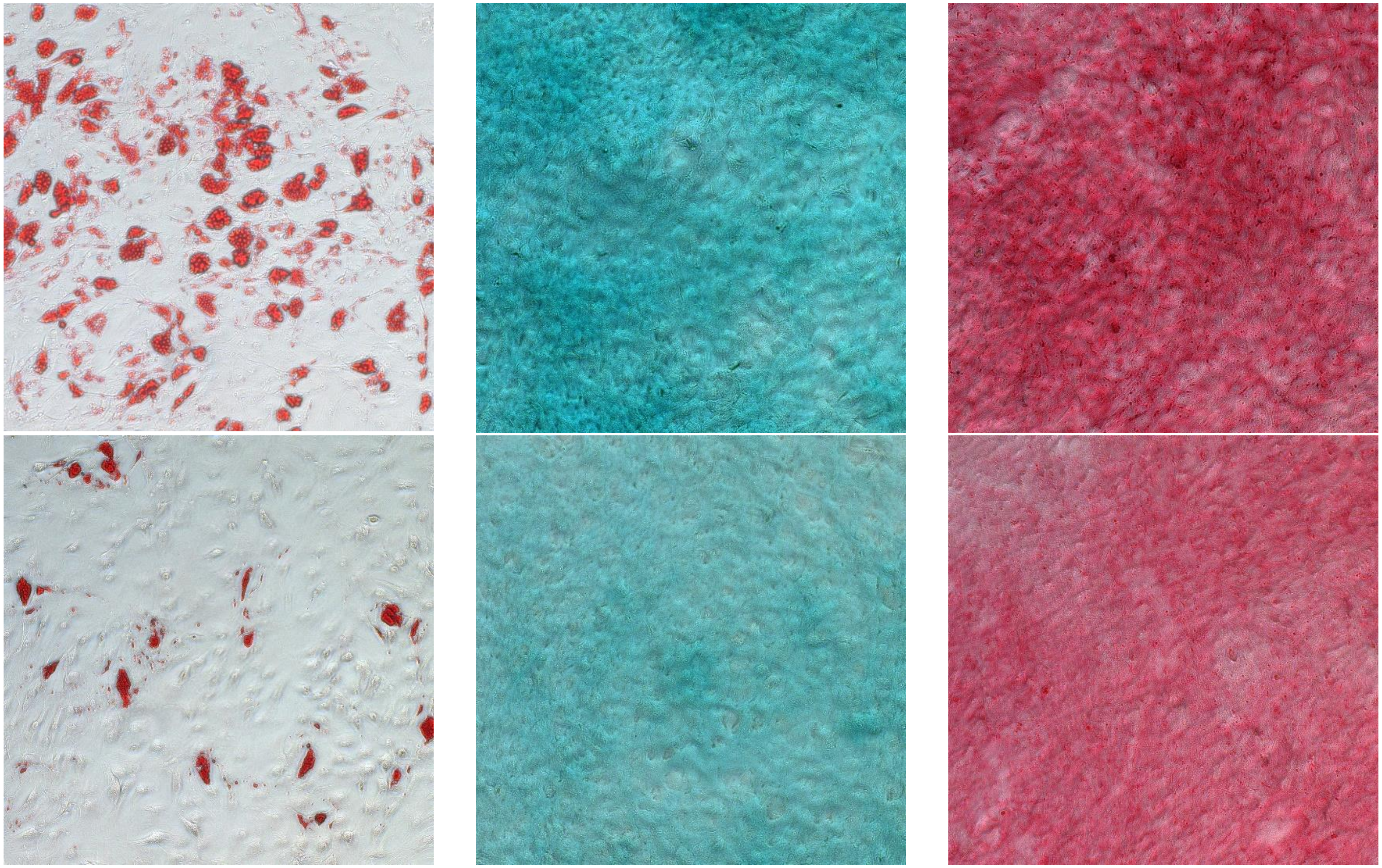

Continued on next page

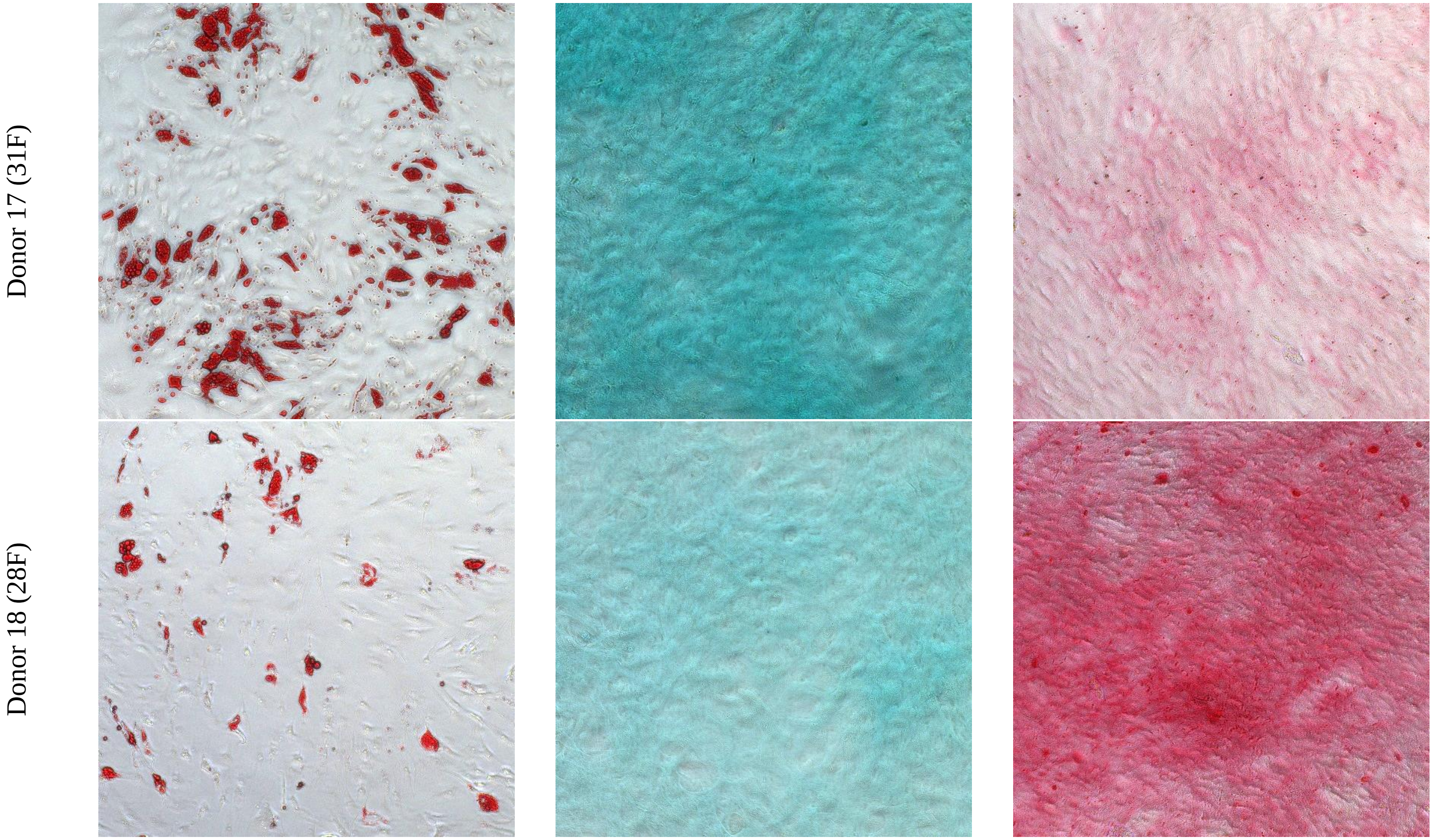

Continued on next page

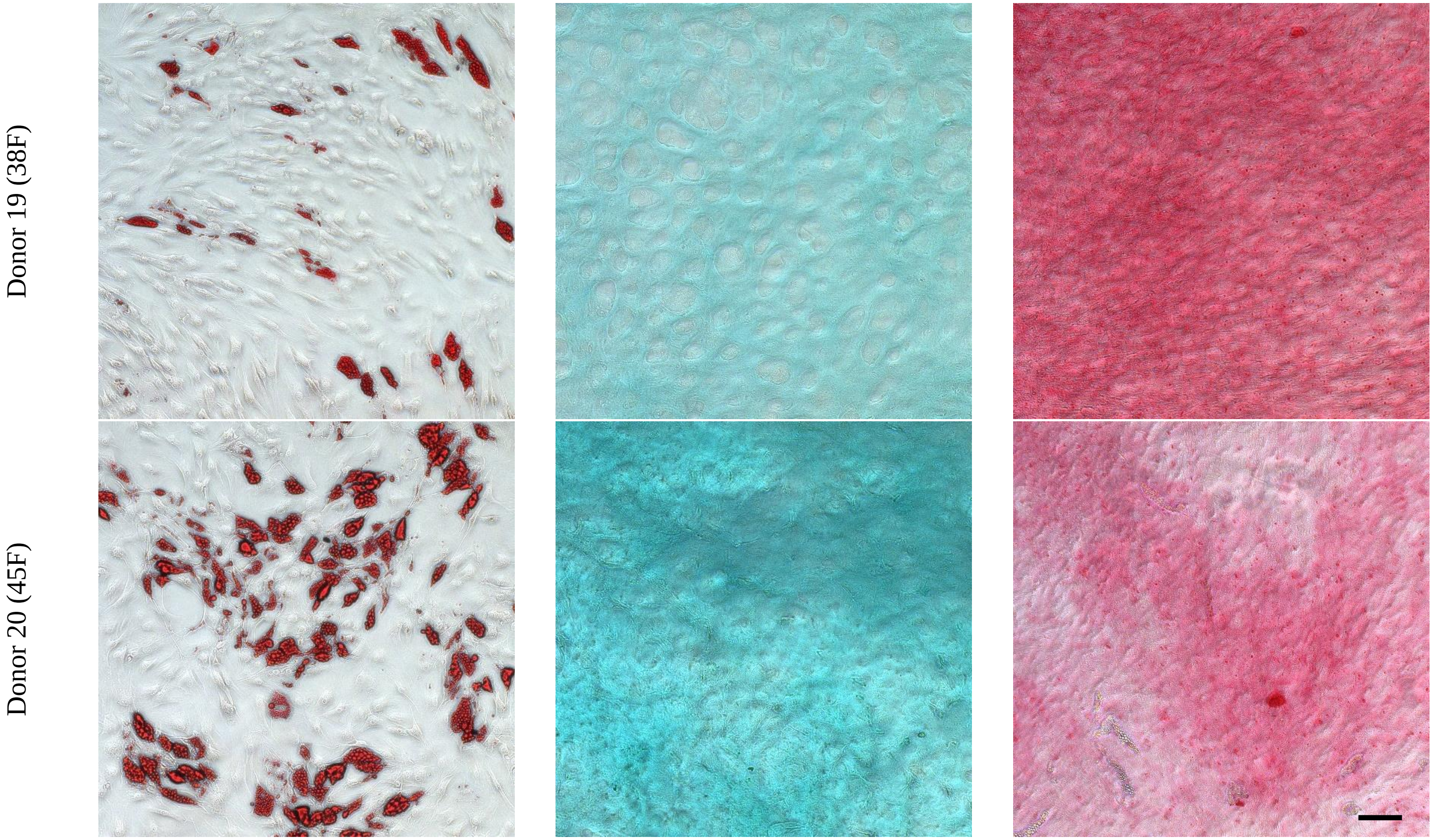

Figure S24. Representative Oil Red O, Alcian Blue and Alizarin Red staining images showing the trilineage differentiation of hBMSCs from 20 different donors. The deidentified patients are indicated by their age and gender (F, female; M, male). Scale bar = 100 μm.

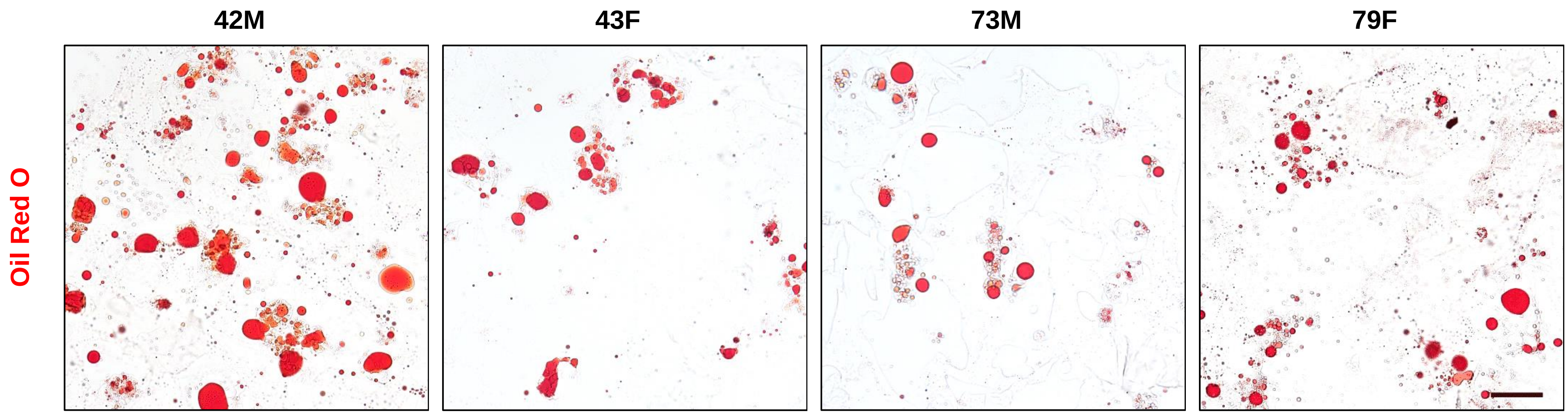

Figure S25. Human bone marrow-derived MSCs (hBMSCs) from four different donors were inducted to deposit lipid droplets in 3D methacrylated gelatin (GelMA) scaffolds. Representative Oil Red O staining images show the presence of oil droplets in the cryosections of engineered 3D adipose tissue (AT) after 4 weeks of culture in adipogenic medium (AM). The donors are denoted by their age and gender (F, female; M, male). Scale bar = 50 µm.

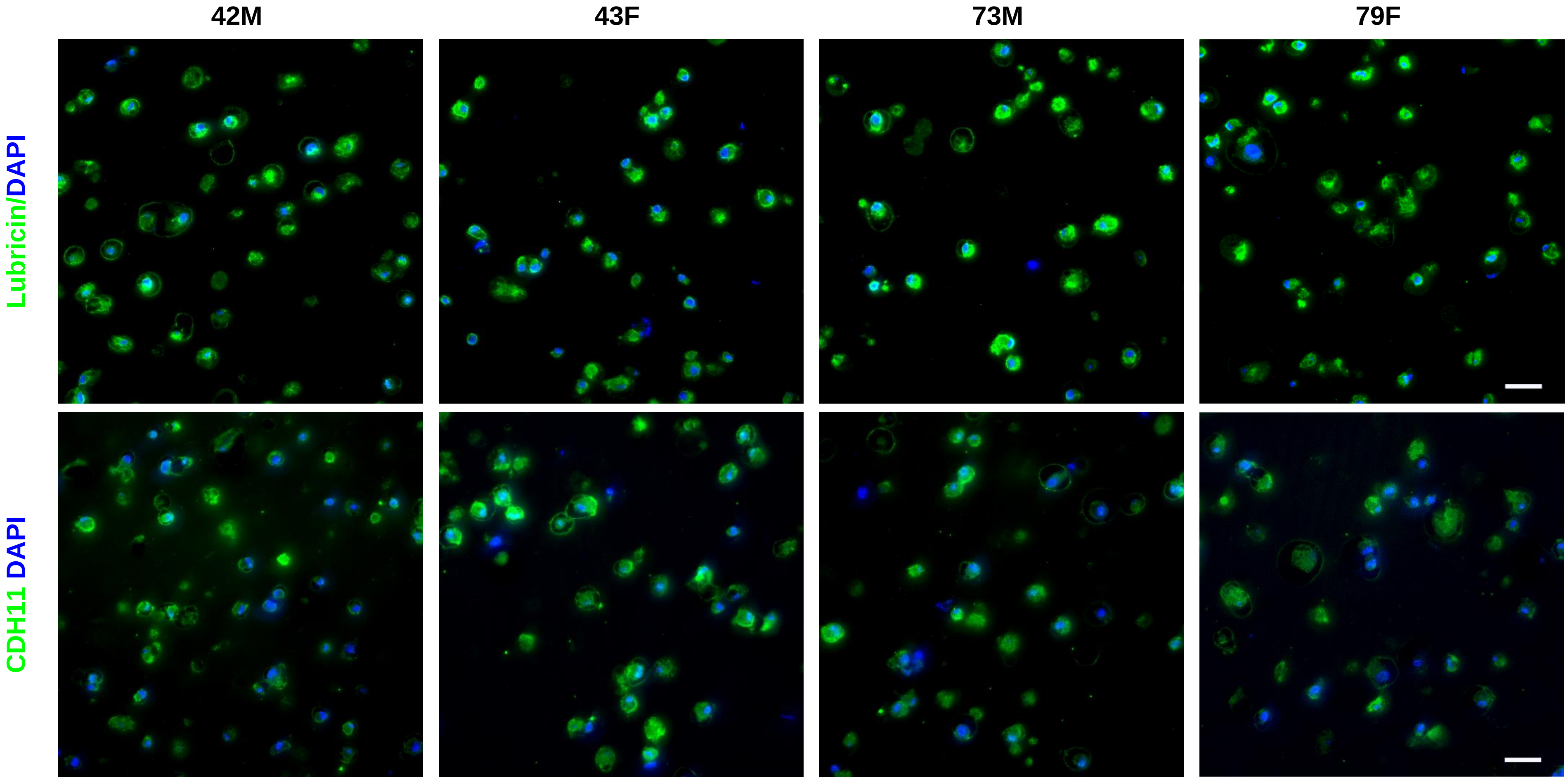

Continued on next page

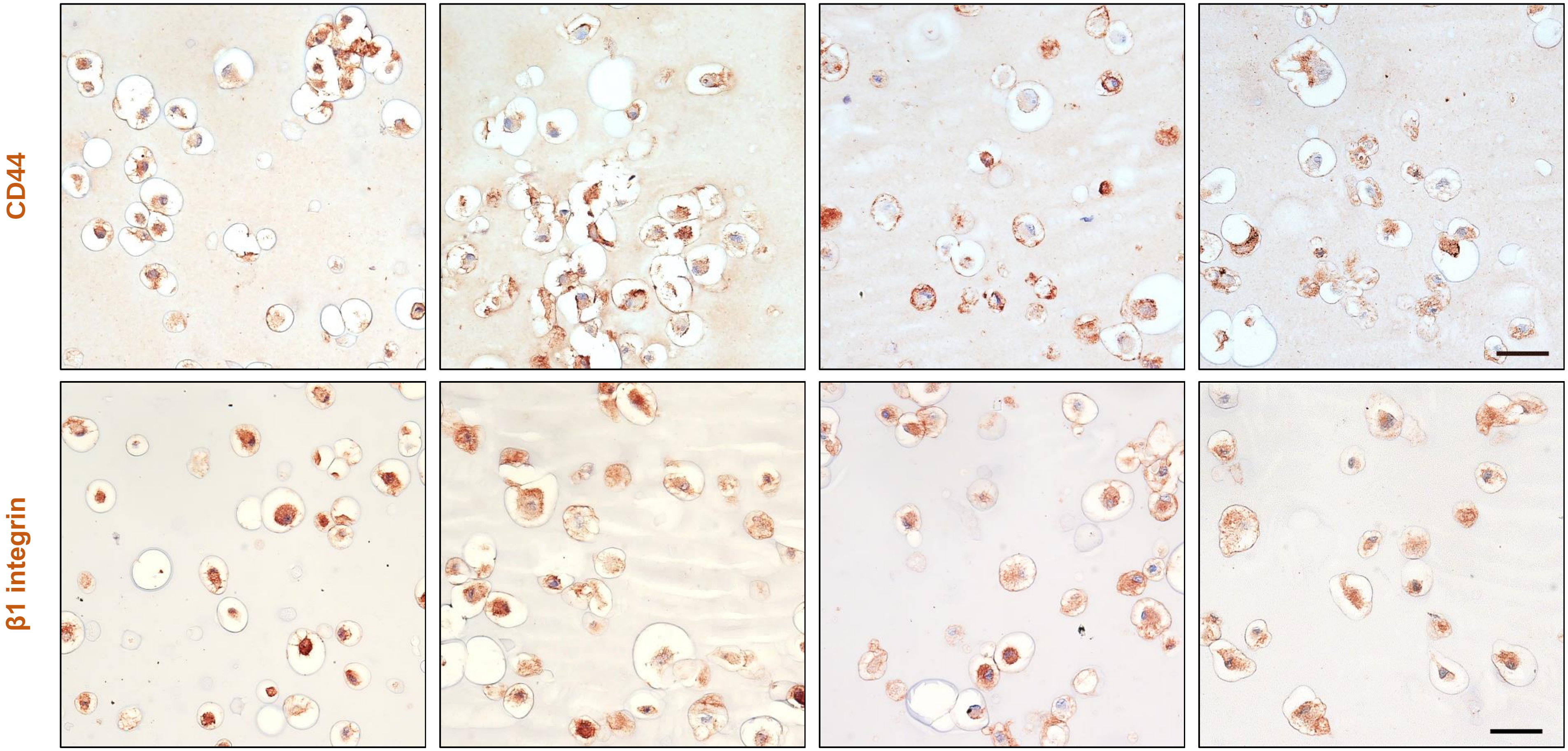

Figure S26. Synovial-like fibrous tissue (SFT) engineered with hBMSCs from four different donors expressed markers associated with synovial fibroblasts. The expression of lubricin and cadherin 11 (CDH11) was confirmed by immunofluorescence, and the presence of collagen type 1 (COL1), CD44, and β1 integrin are shown by immunohistochemical staining. The donors are denoted by their age and gender (F, female; M, male). Scale bar = 50 μm.

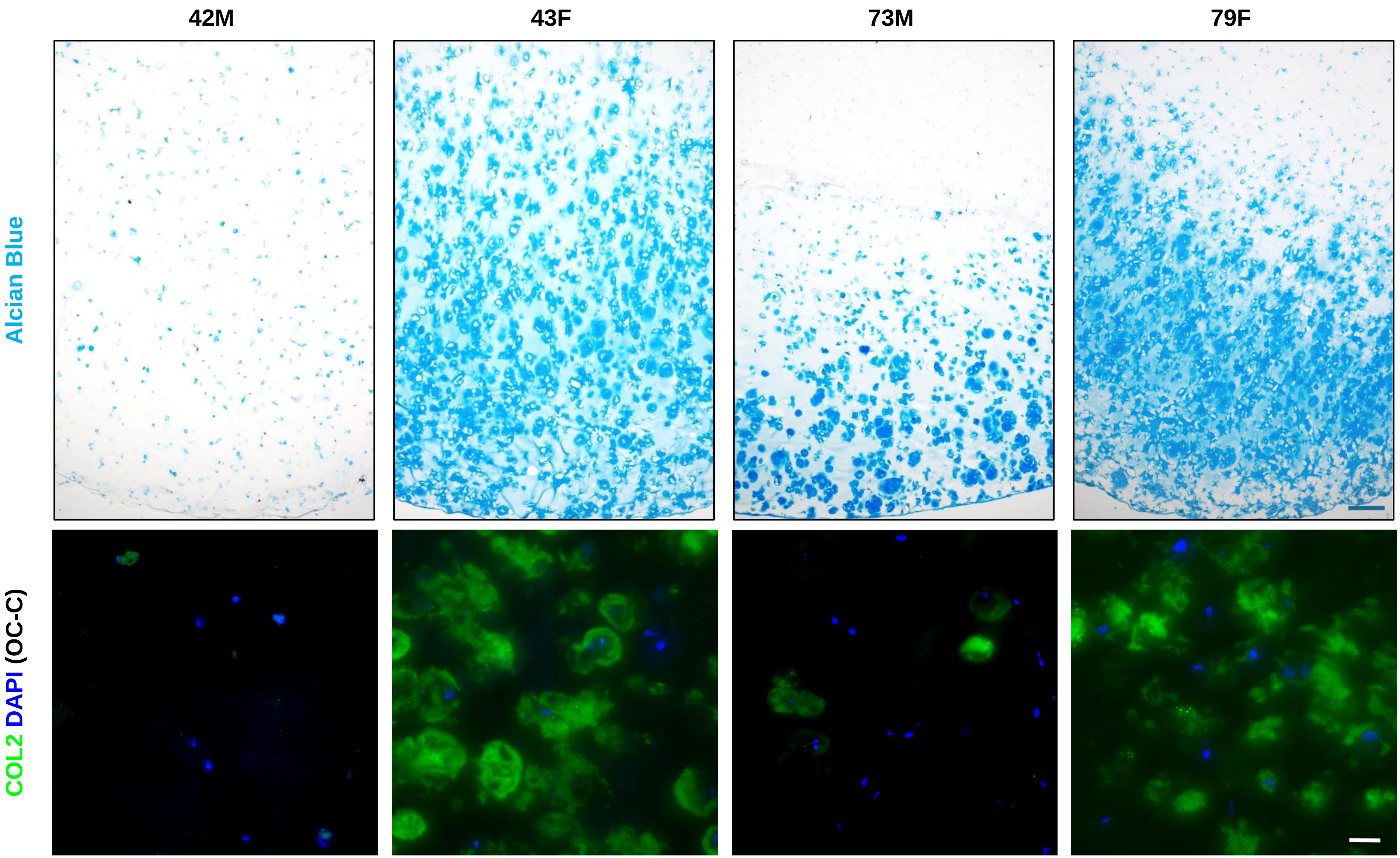

Continued on next page

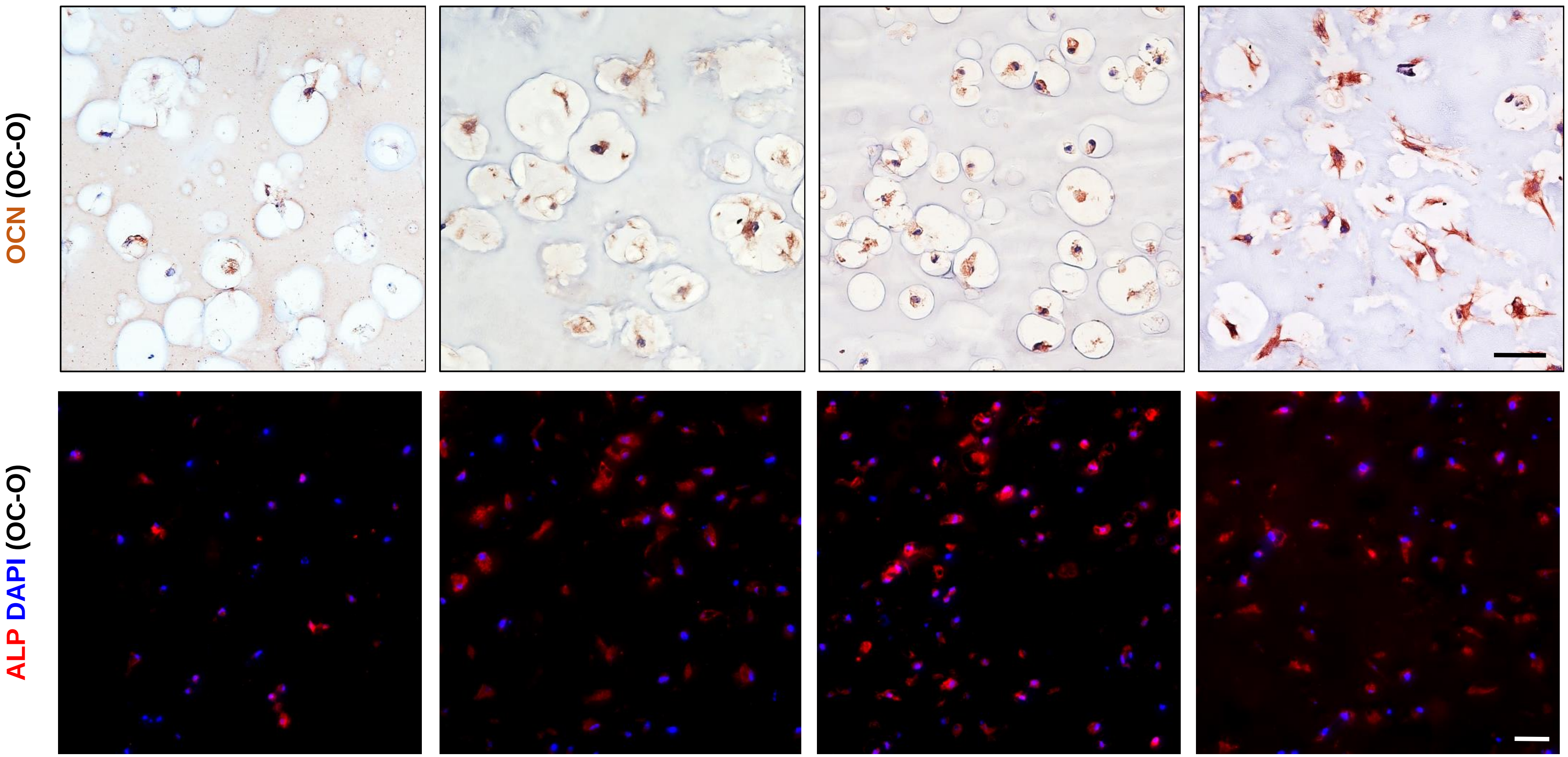

Figure S27. Histological and immunostaining confirmed the phenotypes of the chondral (OC-C) and osseous (OC-O) components of the osteochondral tissues engineered with hBMSCs from four different donors. The matrices of OC-C tissues contained sulfated glycosaminoglycans and collagen type II (COL2), and the OC-O tissues expressed osteocalcin (OCN) and alkaline phosphatase (ALP). The donors are denoted by their age and gender (F, female; M, male). Scale bar = 200 µm for Alcian Blue staining and 50 µm for others.

Supplemental Video

Supplemental Video 1. Medium streams flowing in a miniJoint chip.

References

[1] M. Dominici, K. Le Blanc, I. Mueller, I. Slaper-Cortenbach, F. C. Marini, D. S. Krause, R. J. Deans, A. Keating, D. J. Prockop, E. M. Horwitz, *Cytotherapy* **2006**, *8* (4), 315.

[2] a) W. Thielicke, E. Stamhuis, *Journal of Open Research Software* **2014**, *2* (1), e30; b) W. Thielicke, The flapping flight of birds - Analysis and application, PhD thesis, University of Groningen **2014**.

[3] H. P. Erickson, *Biol. Proced. Online* **2009**, *11* (1), 32.

[4] a) K. B. Goh, H. Li, K. Y. Lam, *Biosensors and Bioelectronics* **2017**, *91*, 673; b) K. B. Goh, H. Li, K. Y. Lam, *ACS Applied Bio Materials* **2018**, *1* (2), 318.

[5] J. C. Bosma, J. A. Wesselingh, *Journal of Chromatography B: Biomedical Sciences and Applications* **2000**, *743* (1), 169.

[6] C. Kotsmar, T. Sells, N. Taylor, D. E. Liu, J. M. Prausnitz, C. J. Radke, *Macromolecules* **2012**, *45* (22), 9177.

[7] a) P. Smeriglio, F. C. Grandi, S. Davala, V. Masarapu, P. F. Indelli, S. B. Goodman, N. Bhutani, *Sci. Transl. Med.* **2020**, *12* (539), eaax2332; b) W. Den Hollander, Y. Ramos, S. Bos, N. Bomer, R. van der Breggen, N. Lakenberg, W. de Dijcker, B. J. Duijnisveld, P. Slagboom, R. G. Nelissen, *Ann. Rheum. Dis.* **2014**, *73* (12), 2208; c) K. M. Fisch, R. Gamini, O. Alvarez-Garcia, R. Akagi, M. Saito, Y. Muramatsu, T. Sasho, J. A. Koziol, A. I. Su, M. K. Lotz, *Osteoarthritis Cartilage* **2018**, *26* (11), 1531; d) Y. F. Ramos, W. den Hollander, J. V. Bovée, N. Bomer, R. van der Breggen, N. Lakenberg, J. C. Keurentjes, J. J. Goeman, P. E. Slagboom, R. G. Nelissen, *PLoS One* **2014**, *9* (7), e103056.
